# Cellular dissection of psoriasis for transcriptome analyses and the post-GWAS era

**DOI:** 10.1186/1755-8794-7-27

**Published:** 2014-05-22

**Authors:** William R Swindell, Philip E Stuart, Mrinal K Sarkar, John J Voorhees, James T Elder, Andrew Johnston, Johann E Gudjonsson

**Affiliations:** 1 Department of Dermatology, University of Michigan School of Medicine, Ann Arbor, MI 48109-2200, USA

**Keywords:** AP-1, Fibroblast, GWAS, Keratinocyte, Microarray, Neutrophil, TNFRSF9, Transcription factor

## Abstract

**Background:**

Genome-scale studies of psoriasis have been used to identify genes of potential relevance to disease mechanisms. For many identified genes, however, the cell type mediating disease activity is uncertain, which has limited our ability to design gene functional studies based on genomic findings.

**Methods:**

We identified differentially expressed genes (DEGs) with altered expression in psoriasis lesions (*n* = 216 patients), as well as candidate genes near susceptibility loci from psoriasis GWAS studies. These gene sets were characterized based upon their expression across 10 cell types present in psoriasis lesions. Susceptibility-associated variation at intergenic (non-coding) loci was evaluated to identify sites of allele-specific transcription factor binding.

**Results:**

Half of DEGs showed highest expression in skin cells, although the dominant cell type differed between psoriasis-increased DEGs (keratinocytes, 35%) and psoriasis-decreased DEGs (fibroblasts, 33%). In contrast, psoriasis GWAS candidates tended to have highest expression in immune cells (71%), with a significant fraction showing maximal expression in neutrophils (24%, P < 0.001). By identifying candidate cell types for genes near susceptibility loci, we could identify and prioritize SNPs at which susceptibility variants are predicted to influence transcription factor binding. This led to the identification of potentially causal (non-coding) SNPs for which susceptibility variants influence binding of AP-1, NF-κB, IRF1, STAT3 and STAT4.

**Conclusions:**

These findings underscore the role of innate immunity in psoriasis and highlight neutrophils as a cell type linked with pathogenetic mechanisms. Assignment of candidate cell types to genes emerging from GWAS studies provides a first step towards functional analysis, and we have proposed an approach for generating hypotheses to explain GWAS hits at intergenic loci.

## Background

Psoriasis is a chronic disease in which interactions between immune cells and keratinocytes (KCs) lead to development of plaque-like lesions with adherent silvery scales. To better understand molecular mechanisms underlying this process, investigators have applied genome-scale technologies, such as expression profiling and genome-wide association studies (GWAS) [1-4]. This has provided a wealth of new data, although there remain many gaps between the individual genes uncovered from these approaches and our current understanding of psoriasis pathogenesis [5]. In this respect, a key challenge is that both approaches, expression profiling and GWAS, essentially provide pointers to individual genes, but do not necessarily provide indication as to which cell type serves as the main context for a gene’s disease activity [3,6-8]. This challenge is compounded by the complex and multicellular nature of psoriasis, with lesions arising from the interactions among a number of heterogeneous cell types, such as T-cells, dendritic cells, macrophages and KCs [9]. Even from an optimistic standpoint, therefore, it must be acknowledged that genome-scale approaches will provide us only with pieces of the puzzle (i.e., individual genes) [1,2]. Putting this puzzle together will require conceptual bridges that connect genetics with biology, and this, in turn, is almost certain to require a high quality mapping between putative disease genes and their relevant cell type(s).

There has been historical tension between the idea of psoriasis as a skin disease, affecting mostly KCs, and the understanding of psoriasis as an (auto)-immune disease, involving cells from the adaptive and/or innate immune systems [10-14]. Early on, psoriasis was viewed primarily as a skin disease, and was treated entirely with topical agents, such as coal tar derivatives, salicylic acid and corticosteroids [15,16]. The understanding of psoriasis as an immune disease, however, was bolstered by the efficacy and widespread adoption of immunosuppressant drugs (e.g., cyclosporine) [17]. This has been abundantly reinforced, more recently, by the success of biologic therapies designed to block inflammatory cytokines or inhibit lymphocyte activation (e.g., etanercept, alefacept and efaluzimab) [18]. The progression in psoriasis treatments thus reflects increased emphasis on immune cells, and T-cells in particular, as central players in psoriasis pathogenesis [16]. Still, when transplanted onto immunodeficient mice, uninvolved skin from psoriasis patients develops into psoriasis lesions, whereas normal skin from normal subjects does not [19]. This suggests that a skin-intrinsic defect is required for development of psoriasis lesions, and it is possible that this underlying skin abnormality initiates or partly drives the inflammatory response and aggregation of immune cells within lesions [20]. A balanced view of psoriasis, therefore, appears most justifiable, in which psoriasis is understood as a cutaneous/immune hybrid disease, dependent upon pathological interactions between KCs and immune cells, as well as interactions among the immune cells themselves. Moreover, insofar as immune cells are accepted as primary drivers of psoriasis, the relative contributions of the innate and adaptive immune systems remains unclear [14,21]. Macrophages, NK cells and neutrophils, for example, are all present in psoriasis lesions and reinforce inflammatory cascades by releasing cytokines while activating other inflammatory cell types, including T-cells [22-24].

Genome-scale approaches can provide insights into the relative importance of KCs and the balance between innate and adaptive immunity in psoriasis pathogenesis [1-4]. Studies of genome-wide expression have proven informative with regard to the composition of psoriasis lesions and have been used to assess which inflammatory cells show increased abundance relative to uninvolved skin [3,6]. One prior meta-analysis of microarray data, for instance, showed that genes with elevated expression in psoriasis lesions were enriched with genes expressed at high levels in KCs, as expected, but also enriched with genes expressed at high levels in cell types from the adaptive (T-cells) and innate immune systems (macrophages and NK-cells) [3]. GWAS findings, moreover, have the potential to identify cell types acting as instigators in the disease process [7,8,25]. Along these lines, the importance of the adaptive immune system in psoriasis has long been supported by strong genetic associations involving genes functioning in antigen presentation (i.e., *HLA-C*) [1,2]. It was especially notable, therefore, when a recent psoriasis GWAS meta-analysis uncovered associations that implicated genes functioning within the innate immune system (e.g., *DDX58*, *KLF4*, *ZC3H12C*, *CARD14* and *CARM1*) [26]. Alongside these results, nearly all GWAS studies to date have also uncovered some loci near genes with KC-specific functions (e.g., *LCE3D*), thus implicating disease mechanisms intrinsic to the skin [11-13]. Authors have often interpreted such results to support the relevance of particular cell types in psoriasis, but no systematic analyses have been carried out to associate GWAS findings with cell types specific to skin, innate immunity, or adaptive immunity. As we move into the post-GWAS era, however, such analyses will be necessary and can indeed provide a first step towards functional studies aimed at understanding how risk variants contribute to disease mechanisms [7].

The goal of this study is to extend findings from psoriasis transcriptome and GWAS analyses to establish connections between genes and disease-relevant cell types. We provide an updated meta-analysis of the psoriasis transcriptome, based upon data from prior studies that have utilized a common microarray platform (*n* = 216 patients total) [6,27-31]. Using these data, we identify differentially expressed genes (DEGs) within psoriasis lesions, assign a candidate cell type to each DEG, and assess overall trends with respect to the assigned cell types. Following the same strategy, we identify genes associated with psoriasis susceptibility loci [26,32,33], assign cell types to each candidate gene, and characterize overall trends. Our findings provide new insights into the relative role of KCs, adaptive immunity and innate immunity in psoriasis, while providing a first step towards post-GWAS functional analyses. To illustrate this latter point, we develop a systematic approach for functional interpretation of intergenic susceptibility loci. The approach requires assignment of candidate cell types to candidate genes and identifies potentially causal (non-coding) SNPs at which variation is predicted to influence transcription factor binding.

## Results

### Meta-analysis identifies 1904 differentially expressed genes (DEGs) with significantly altered expression in psoriasis lesions (*n* = 216 patients)

We evaluated gene expression in lesional (PP) and uninvolved (PN) skin samples across a large patient cohort (*n* = 216 patients). Our analysis included 16358 skin-expressed genes for which expression was detectable in at least 10% of the 432 biopsies (PP and PN) from 216 patients. In total, we identified 1019 PP-increased DEGs (FC > 1.50 and FDR < 0.05; Additional file 1) along with 885 PP-decreased DEGs (FC < 0.67 and FDR < 0.05; Additional file 2). Expression of PP-increased DEGs was, on average, detected above background in 94% of PP samples and 84% of PN samples (Additional file 3, Parts A and B). Likewise, expression of PP-decreased DEGs was, on average, detected above background in 86% of PP samples and 94% of PN samples (Additional file 3, Parts C and D). These percentage values were significantly large in comparison to all other genes included in our analysis (P ≤ 0.02; Additional file 3). We were unable to identify any DEGs for which expression was decreased in all 216 patients. However, we identified 13 DEGs for which expression was increased in all 216 patients (*IL36G*, *KYNU*, *RHCG*, *ATP12A*, *HPSE*, *CCL20*, *PRSS27*, *FUT2*, *SERPINB13*, *WNT5A*, *LOC100996579*, *C20orf24* and *SOX7*). These may be regarded as hallmark genes with universally or near-universally increased expression in psoriasis lesions.

### Psoriasis DEGs overlap significantly with genes altered in diverse skin diseases (squamous cell carcinoma, atopic dermatitis, Mediterranean spotted fever eschars)

**Figure 1:**
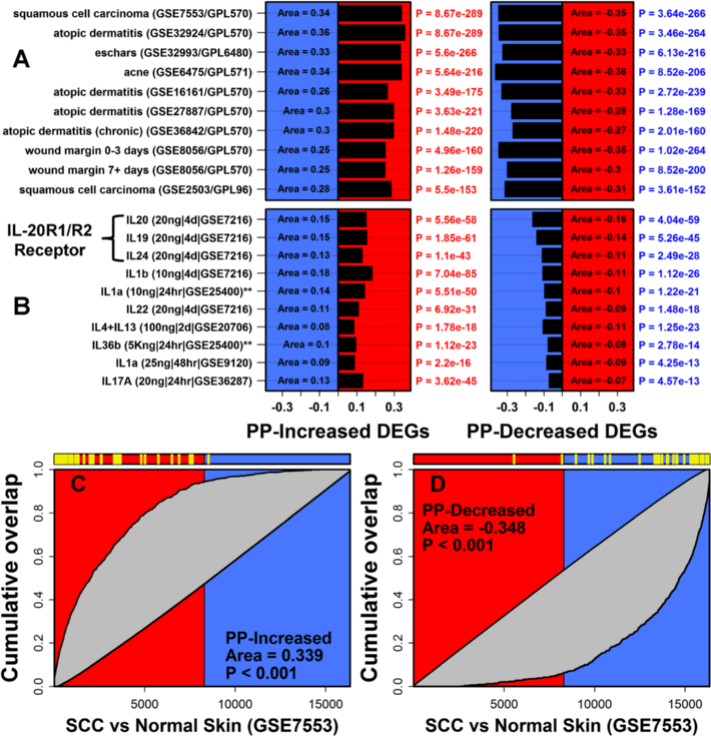
**Gene set enrichment analysis: Psoriasis DEGs overlap significantly with genes altered in other skin diseases and in cytokine-treated KCs.** We identified 1019 PP-increased DEGs and 885 PP-decreased DEGs (*n* = 216 patients) and evaluated how these genes were altered in other skin diseases or experiments with cytokine-treated KCs. **(A)** Comparison with skin diseases. Psoriasis DEGs were compared to ranked gene lists generated from microarray studies comparing diseased skin samples with normal or uninvolved skin. Barplots show GSEA area statistics calculated for each comparison (i.e., area between foreground and background detection rate curves; see parts **C** and **D**). Positive area statistics indicate that psoriasis DEGs are disproportionately elevated in a given skin disease, while negative statistics indicate that psoriasis DEGs are disproportionately repressed. The analysis was performed with respect to PP-increased DEGs and PP-decreased DEGs, and comparisons are ranked according to the maximum p-value from the two analyses (Wilcoxon rank sum test). The top-ranked 10 comparisons are shown (of 51 included in our screen). **(B)** Comparison with cytokine-treated KCs. The analysis was repeated with respect to ranked gene lists generated from microarray experiments in which cultured KCs were treated with cytokines. Left margin labels list the concentration of cytokine (per mL) and the duration of treatment. The top-ranked 10 comparisons are shown (of 46 included in our screen). Most experiments were performed with primary NHEKs (** denotes experiments performed using reconstituted epidermis). **(C)** GSEA analysis of psoriasis-increased DEGs and squamous cell carcinoma (SCC). Genes were ranked according to how strongly they were increased in SCC as compared to normal skin (GEO series GSE7553). The figure shows the cumulative overlap between psoriasis-increased DEGs and this ranked gene list. Significance of this overlap is proportional to the area between this curve and the diagonal (i.e., grey region). Yellow hashmarks (top) denote the 100 DEGs most strongly elevated in psoriasis lesions (i.e., highest PP/PN fold-change). **(D)** GSEA analysis of psoriasis-decreased DEGs and SCC. Yellow hashmarks (top) denote the 100 DEGs most strongly repressed in psoriasis lesions (i.e., lowest PP/PN fold-change).

Comparison of psoriasis DEGs to genes altered in a diverse set of skin diseases revealed strong correspondence (Figure 1). The strongest correspondence was observed for squamous cell carcinoma, atopic dermatitis and Mediterranean spotted fever eschars, although significant correspondence was also detected for other skin conditions as well (e.g., acne, wounding, nickel contact dermatitis; Figure 1 and Additional file 4). For these conditions, PP-increased DEGs overlapped significantly with genes elevated in diseased skin, while conversely, PP-decreased DEGs overlapped significantly with genes repressed in diseased skin (Figure 1 and Additional file 4). We next compared psoriasis DEGs to those genes altered in microarray experiments in which cultured KCs (or skin equivalents) were treated with cytokines. This highlighted the IL-20R1/IL-20R2 receptor complex cytokines (IL-20, IL-19 and IL-24) as those best able to induce expression of PP-increased DEGs and repress expression of PP-decreased DEGs (Figure 1 and Additional file 4).

Psoriasis DEGs were disproportionately elevated or repressed in cultured KCs following RNAi treatments targeting differentiation-associated genes or lncRNAs (Additional file 4). PP-increased DEGs were elevated in KCs following knockdown of the anti-differentiation lncRNA *ANCR* (P = 5.8 × 10^−25^, GSEA) [34], but decreased following knockdown of the pro-differentiation gene *TP63* (P = 1.2 × 10^−25^, GSEA) [35]. PP-decreased DEGs, moreover, were elevated in KCs following knockdown of the pro-differentiation gene *MLL2* (P = 9.4 × 10^−36^, GSEA) [36]. Finally, inspection of psoriasis DEGs revealed trends related to DNA methylation [37]. PP-increased DEGs were disproportionately associated with hypo-methylated DNA sites in psoriasis lesions (P = 4.8 × 10^−13^, GSEA), while conversely, PP-decreased DEGs were disproportionately associated with hyper-methylated DNA sites (P = 6.8 × 10^−15^, GSEA).

### Assignment of psoriasis DEGs and GWAS candidates to cell types present in lesional skin

Psoriasis is a complex disease involving interactions among multiple cell types from skin and the adaptive/innate immune systems [9]. For psoriasis DEGs, it is unclear which cell types underlie shifts in gene expression, and similarly, it is often uncertain which cell types mediate disease-associated effects of genes near susceptibility loci [3,6,7]. To address these issues, we compiled a large database of microarray samples from 10 different cell types: primary KCs, fibroblasts, CD4+ T-cells, NK cells, CD8+ T-cells, B cells, macrophages, monocytes, dendritic cells (DCs) and neutrophils (Additional file 5). For each cell type, a large number of microarray samples was obtained, with no fewer than 118 samples for any one cell type (Additional file 5).

**Figure 2:**
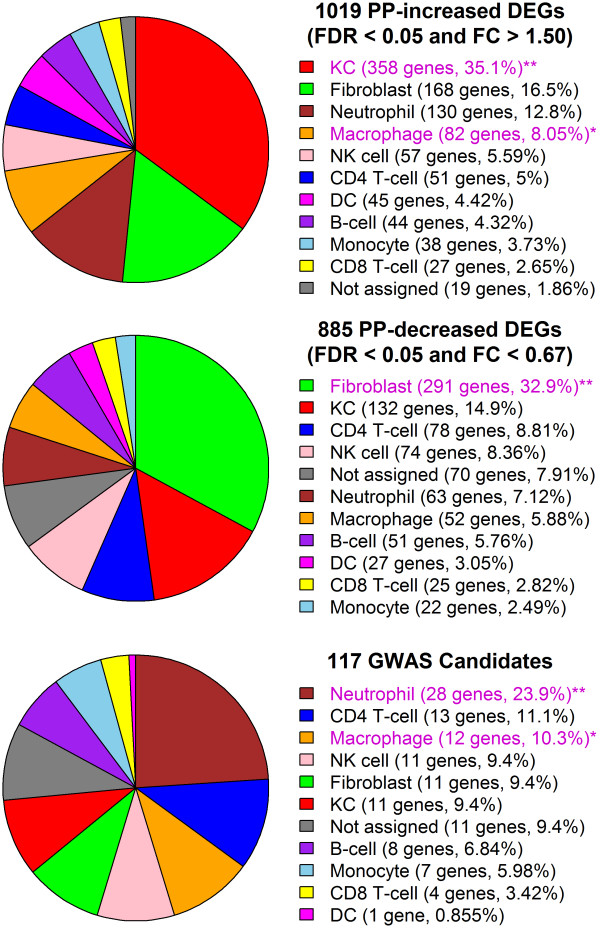
**Assignment of psoriasis DEGs and GWAS candidates to cell types present in lesional skin.** Human genes were assigned to one of 10 cell types present in psoriasis lesions. Genes were assigned to the cell type for which median expression was highest, provided that the gene’s expression was detected in at least 10% of microarray samples for that cell type (P < 0.05, Wilcoxon signed-rank test). If a gene’s expression was not detected with respect to at least 10% of microarray samples for any cell type, no assignment was made (i.e., unassigned). Pie charts show the percentage of PP-increased DEGs (top), PP-decreased DEGs (middle) and GWAS candidates (bottom) that were either unassigned or allocated to one of the 10 cell types. Magenta labels denote those cell types for which the number of assigned genes was significantly large in comparison to all skin-expressed genes (PP-increased and PP-decreased DEGs) or in comparison to all genes represented on the Affymetrix Human Genome U133 Plus 2.0 array platform (GWAS candidates) (one asterisk, P < 0.05; two asterisks, FDR < 0.05; Fisher’s Exact Test).

The database was used to assign a candidate cell type to each human gene based upon the gene’s expression level across the 10 cell types. For each gene, we identified the cell type for which the gene’s median expression was highest, provided that expression was detected in at least 10% of microarray samples (P < 0.05, Wilcoxon signed rank test) [38]. No assignment was made if a gene’s expression was not detected in at least 10% of microarray samples for any cell type (P < 0.05). After assignments were made, we assessed trends among PP-increased and PP-decreased DEGs, as well as among candidate genes identified from psoriasis GWAS studies (Figure 2).

### Psoriasis-increased DEGs are enriched with genes expressed at high levels in KCs and macrophages

**Figure 3:**
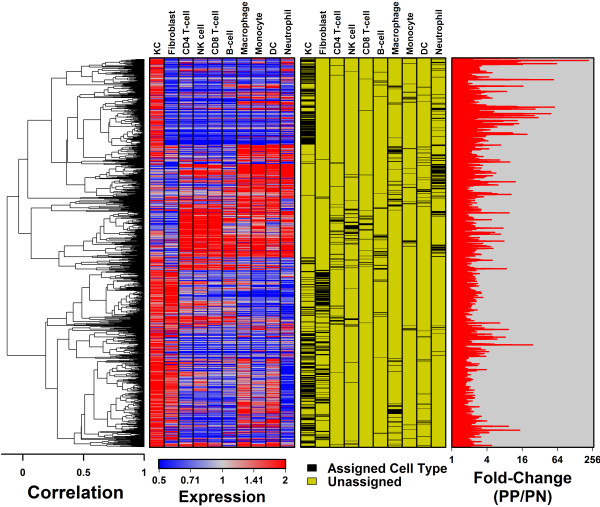
**Cluster analysis of 1019 genes elevated in psoriasis lesions and their expression across 10 cell types.** We identified 1019 PP-increased DEGs with PP/PN fold-change greater than 1.50 and FDR less than 0.05 (*n* = 216 patients; Wilcoxon rank sum test). These genes were clustered based upon their expression pattern across 10 cell types (Spearman correlation distance and average linkage). The red-blue heatmap shows expression of genes in each cell type, with red colors indicating relatively high expression (compared to normal human skin) and blue colors indicating relatively low expression (compared to normal human skin). The yellow-black heatmap shows the cell type assigned to each gene (i.e., the cell type for which the gene’s median expression was highest, with detection frequency greater than 10%). The chart on the far right shows the estimated median fold-change (PP/PN) for each gene (*n* = 216 patients).

Approximately 50% of PP-increased DEGs were assigned to KCs or fibroblasts, while the remaining 50% were assigned to immune cell types. PP-increased DEGs were enriched with respect to the number of genes assigned to KCs and macrophages (P < 0.05; Fisher’s Exact Test; Figures 2 and 3). These trends were further supported by rank-based analyses, which showed that KC- and macrophage-assigned genes tended to have elevated expression in PP versus PN skin (P ≤ 2.2 × 10^−20^; GSEA; Additional file 6, Part A). A non-parametric bootstrap analysis also indicated that PP-increased DEGs, on average, had higher-than-expected expression and detection frequency only for KCs and macrophages, but not for other cell types (Additional file 7). 35% of PP-increased DEGs (358/1019) were expressed more highly in KCs than any other cell type (Figure 2). Examples of KC-assigned genes strongly elevated in psoriasis lesions included *SERPINB4*, *SPRR2C* and *SERPINB3* (Additional file 8). Consistent with heightened KC proliferation, KC-assigned PP-increased DEGs were frequently associated with organelle fission, cell division and the cell cycle (data not shown). Approximately 8% of PP-increased DEGs (82/1019) were assigned to macrophages (Figure 2), the strongest examples of which included *KYNU*, *ADAMDEC1* and *CXCL13* (Additional file 8). These and other macrophage DEGs were commonly associated with response to biotic stimulus, immune response and programmed cell death (data not shown).

PP-increased DEGs were not significantly enriched with respect to several inflammatory cell types, such as CD4+ T-cells and dendritic cells (Figure 2). Notably, among 438 DEGs elevated more than 2-fold in psoriasis lesions (rather than 1.5-fold), there was significant enrichment for neutrophil-assigned genes (64/438 DEGs; P = 0.031, Fisher’s Exact Test). For other inflammatory cell types, however, lack of enrichment is likely due to the fact that DEGs include only genes showing the most *consistent* expression patterns among psoriasis lesions, excluding those genes associated with more variable patterns, such as those that might be associated with infiltration by certain immune cells [3,6]. To test this idea, we calculated cell type-specific signatures for each of the 216 patients, which allowed us to quantify the degree to which genes showing specific expression in a given cell type were elevated or repressed within individual patients [3]. Consistent with our expectation, genes specifically expressed by most immune cell types showed a trend towards elevated expression in some patients, but not others (Additional file 9). Genes specifically expressed by KCs, in contrast, showed a significant trend towards PP-increased expression in nearly all patients (86%) (Additional file 9).

### Psoriasis-decreased DEGs are enriched with genes expressed at high levels in fibroblasts and reticular dermis

**Figure 4:**
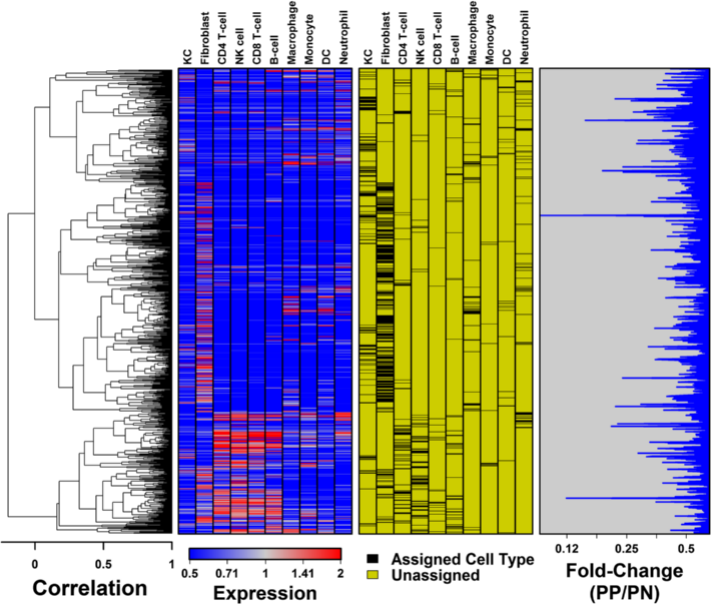
**Cluster analysis of 885 genes decreased in psoriasis lesions and their expression across 10 cell types.** We identified 885 PP-decreased DEGs with PP/PN fold-change less than 0.67 and FDR less than 0.05 (*n* = 216 patients; Wilcoxon rank sum test). These genes were clustered based upon their expression pattern across 10 cell types (Spearman correlation distance metric and average linkage). The red-blue heatmap shows the expression of each gene in each cell type, with red colors indicating relatively high expression (compared to normal human skin) and blue colors indicating relatively low expression (compared to normal human skin). The yellow-black heatmap shows the cell type assigned to each gene (highest median expression with detection frequency greater than 10%). The chart on the far right shows the estimated median fold-change (PP/PN) for each gene (*n* = 216 patients).

PP-decreased DEGs included a disproportionate number of genes assigned to fibroblasts (P < 0.05; Fisher’s Exact Test; Figures 2 and 4). This trend was reinforced by a rank-based analysis, which showed that fibroblast-associated genes tended to have decreased expression in PP versus PN skin (P = 1.2 × 10^−97^; GSEA; Additional file 6, Part B). Additionally, whereas PP-decreased DEGs usually had lower-than-expected detection frequency and expression in most cell types, this wasn’t the case for fibroblasts; in contrast, PP-decreased DEGs had higher-than-expected detection frequency in fibroblasts on average (P = 0.048; Additional file 10). Moreover, for nearly all patients (84%), fibroblast signature scores were significantly low (P < 0.05), indicating that genes specifically expressed by fibroblasts tend to decline in PP skin (Additional file 9). Consistent with these results, PP-decreased genes were enriched with respect to genes showing elevated expression in LCM-dissected reticular dermis in comparison to normal human skin (P = 1.2 × 10^−46^; GSEA; data not shown). 33% of PP-decreased DEGs (291/885) were expressed more highly in fibroblasts than any other cell type (Figure 2), of which *TSPAN8* and *PAMR1* were among the most strongly repressed in psoriasis lesions (Additional file 11). PP-decreased DEGs assigned to fibroblasts were associated with functions involving development, morphogenesis and cell adhesion (data not shown).

### Psoriasis and innate immunity: candidate genes from psoriasis GWAS studies are enriched for genes expressed at high levels in neutrophils

Genome-wide association studies (GWAS) of psoriasis have been instrumental in identifying genes likely to be involved in the psoriasis disease process [1,2]. We evaluated 117 candidate genes near psoriasis susceptibility loci, including those listed in the NHGRI GWAS catalogue and those identified in a recent psoriasis meta-analysis (see Methods) [26]. Most GWAS candidates were assigned to immune cell types, with a disproportionately large number assigned to neutrophils and macrophages (neutrophils, P = 1.5 × 10^−5^; macrophages, P = 0.012; Fisher’s Exact Test; Figure 2). Consistent with these trends, the candidate genes, on average, had higher-than-expected detection frequency and expression in all immune cell types (P ≤ 0.059), but this was not the case with respect to KCs and fibroblasts (Additional file 12). GWAS candidates assigned to neutrophils were associated with diverse immune functions, such as cytokine production, response to other organism, and innate immune response (data not shown).

We also analyzed a set of 357 psoriasis-associated genes from the Human Genome Epidemiology Network (HuGENet) database [33]. This set includes candidate genes from psoriasis GWAS studies, but in addition includes genes identified from non-GWAS epidemiologic population-based studies [33]. In agreement with the above analyses, the 357 HuGENet genes were enriched with neutrophil-assigned genes (Additional file 13). We next combined the 117 GWAS candidates with the 357 HuGENet genes, yielding a maximally comprehensive set of 439 genes associated with psoriasis based upon genetic analyses (Additional files 13 and 14). This set of genes was also significantly enriched with neutrophil-assigned genes, with approximately 19% of genes (82/439) expressed more highly in neutrophils than any other cell type (Additional files 13 and 14).

**Figure 5:**
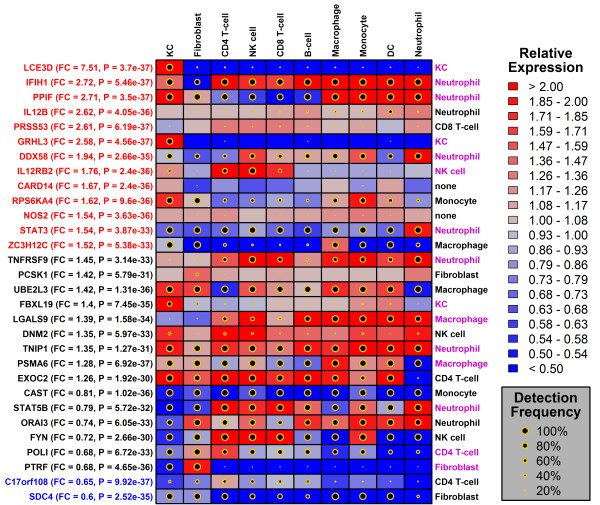
**Top 30 GWAS candidates with altered expression in psoriasis lesions and their expression across 10 cell types.** The table lists the 30 psoriasis GWAS candidates for which expression was most strongly altered in psoriasis lesions (i.e., lowest p-value, Wilcoxon rank sum test; *n* = 216 patients). Each of the listed genes was skin-expressed, with expression detected above background with respect to at least 10% of the 432 PP and PN skin samples from all 216 patients. The color scale denotes median expression of each gene, as compared to the gene’s median expression in normal human skin. Red colors thus denote expression that is higher in comparison to normal human skin, while blue colors indicate that expression is lower in comparison to normal human skin. For each gene and cell type, black circles indicate the percentage of microarray samples for which the gene’s expression was detected above background (P < 0.05, Wilcoxon signed-rank test). Red labels (left margin) denote PP-increased DEGs (FDR < 0.05 with median PP/PN fold-change > 1.5), while blue labels (left margin) represent PP-decreased DEGs (FDR < 0.05 with median PP/PN fold-change < 0.67). The cell type assigned to each gene is listed in the right margin. Magenta labels denote cases in which the gene’s expression in the assigned cell type is significantly higher than its expression in any of the other 9 cell types (FDR < 0.05, Wilcoxon rank sum test).

**Figure 6:**
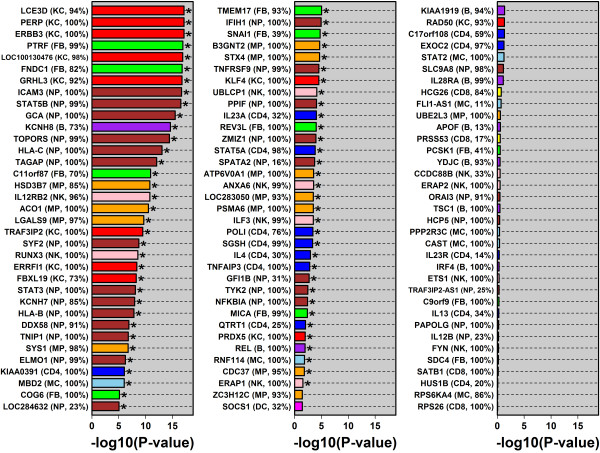
**Cell type assignments for 105 psoriasis GWAS candidate genes.** The left margin lists each individual gene, the assigned cell type, and the gene’s detection frequency with respect to that cell type (KC = keratinocytes; FB = fibroblasts; CD4 = CD4+ T-cells; NK = NK Cells; CD8 = CD8+ T-cells; B = B-cells; MP = macrophage; MC = monocyte; DC = dendritic cells; NP = neutrophils). Genes are ranked by the degree to which they showed specific expression in the assigned cell type. To obtain the p-value shown for each gene, expression in the assigned cell type was compared to expression in each of the other nine cell types, respectively (*n* = 50 samples per cell type; Wilcoxon rank sum test). The p-value shown for each gene corresponds to the largest p-value obtained from each of the 9 comparisons to other cell types (asterisk, FDR < 0.05).

Further inspection of the 117 psoriasis GWAS candidates revealed that, for many, expression was significantly altered in psoriasis lesions, although in most cases estimated fold-changes did not meet DEG criteria (Figure 5). Overall, 13 candidate genes were also PP-increased DEGs, while 2 others were PP-decreased DEGs (Figure 5). Candidate genes varied in the degree to which expression was specific to a given cell type, and this influenced the confidence with which candidate cell types could be assigned (Figure 6). With respect to *LCE3D*, for instance, KCs were assigned as the candidate cell type with high confidence, since expression was distinguishably greater in KCs as compared to any other cell type (Figures 5 and 6). Among the candidate genes for which cell type assignment confidence was greatest, most were assigned to KCs or neutrophils, although some were also assigned to fibroblasts (e.g., *PTRF* and *FNDC1*; see Figure 6).

### Susceptibility-associated variation at rs11121129 disrupts an AP1 binding site associated with *TNFRSF9* expression in neutrophils

The assignment of cell types to candidate genes provides a first step towards an improved functional understanding of susceptibility variants. To illustrate this point, we here develop a procedure for analysis of intergenic risk loci, with the goal of generating hypotheses directed at candidate gene transcription and allele-specific transcription factor binding [39-41]. The procedure requires identification of a candidate cell type for a suspected target gene near a susceptibility locus. Transcription factors (TFs) that potentially regulate the target gene are then identified based upon co-expression analysis. Finally, we identify SNPs at which variation is predicted to influence binding of these TFs. The overall strategy is illustrated below using the *TNFRSF9* risk locus as an example (i.e., rs11121129).

**Figure 7:**
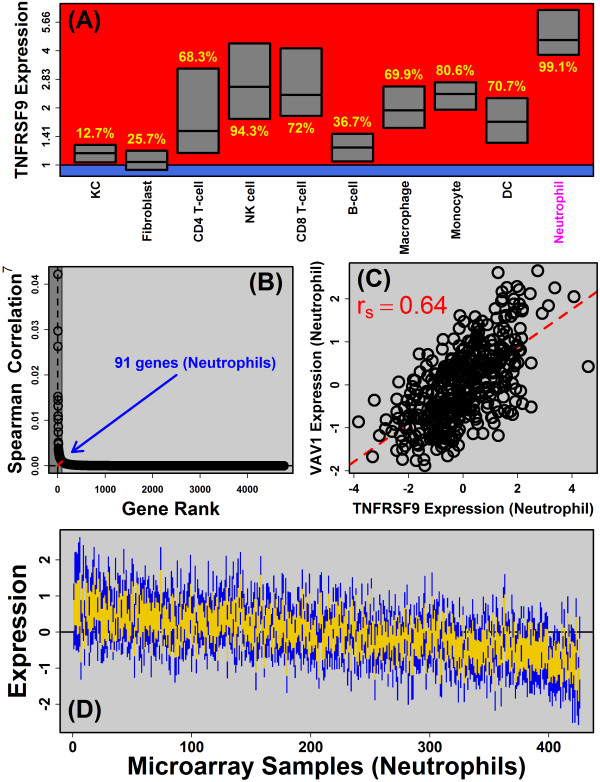
**Identification of 91 genes co-expressed with *TNFSRF9 *in neutrophils. (A)** Expression of *TNFSRF9* across 10 cell types. Expression is normalized to that observed in normal human skin. Values in yellow denote *TNFSRF9* detection frequency. **(B)** Procedure used to identify *TNFSRF9*-co-expressed genes in neutrophils. For genes expressed in both skin and neutrophils, we calculated the Spearman correlation coefficient between the expression pattern of each gene and that of *TNFSRF9* (*n* = 426 neutrophil microarray samples). Genes were ranked based upon the estimated Spearman correlation coefficient (raised to the 7th power). To identify an appropriate cut-off for *TNFSRF9*-co-expressed genes, we located the “bend” in the curve of descending correlation coefficient estimates (i.e., the rank minimizing the length of the red line). **(C)** The gene most strongly associated with *TNFSRF9* expression in neutrophils was *VAV1*. The scatterplot compares normalized expression values (log_2_ scale) between *TNFSRF9* and *VAV1* in neutrophils. **(D)** Expression of 91 genes co-expressed with *TNFRSF9* in neutrophils (*n* = 426 microarray samples). Microarray samples were ordered with *TNFRSF9* expression descending from left to right. Gold regions outline the middle 50% of normalized expression values among the 91 genes. Blue regions outline the middle 80% of normalized expression values among the 91 genes. Expression of each gene was normalized to its baseline (average) expression across all 426 samples, such that positive values correspond to above-average expression and negative values correspond to below-average expression.

Individuals carrying the A allele at rs11121129 (chr1, 8268095) have elevated psoriasis risk (OR = 1.13; P = 1.7 × 10^−8^), but mechanisms underlying this association are not known, since the locus is non-coding and does not affect protein structure [26]. Candidate genes near this locus include solute carrier family 45 member 1 (*SLC45A1*) and tumor necrosis factor receptor superfamily member 9 (*TNFRSF9*). Expression of *SLC45A1* (distance from SNP: 116.3 kb) was rarely detectable in any of the 10 cell types we considered (data not shown). On the other hand, expression of *TNFRSF9* (distance: 264.9 kb) was frequently detected in immune cells and was almost always detected in neutrophils (>99%), which was the cell type for which *TNFRSF9* expression was quantitatively highest (Figure 7A). Consistent with this, we used RT-PCR and flow cytometry to show that *TNFRSF9* mRNA and protein expression was highest in neutrophils, as compared to other cell types, including T-cells and monocytes (Additional file 15). Moreover, in agreement with microarray data (Figure 5), RT-PCR confirmed significant elevation of *TNFRSF9* expression in psoriasis lesions as compared to uninvolved and normal skin (Additional file 15). We thus hypothesized that genetic variation at rs11121129 (or linked SNPs) altered the affinity of sequence-specific DNA-binding factors to influence *TNFRSF9* expression in neutrophils.

**Figure 8:**
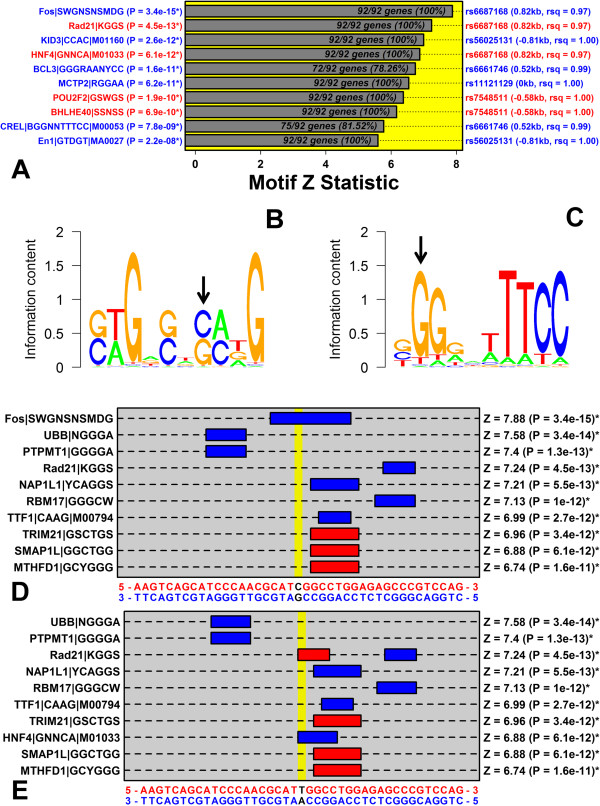
**Susceptibility-associated variation at rs11121129 disrupts an AP1 binding site associated with *TNFRSF9 *expression in neutrophils. (A)** Top 10 binding sites ranked according to their enrichment with respect to sequences adjacent to genes co-expressed with *TNFRSF9* in neutrophils. The listed binding sites include only those that are sensitive to variation at SNPs in linkage disequilibrium with rs11121129 (*r*^2^ > 0.90). The chart shows the *Z* statistic quantifying the degree to which each binding site is enriched among sequences adjacent to *TNFRSF9*-co-expressed genes. Associated p-values are listed in the left margin, where an asterisk symbol is used to denote FDR < 0.05. The right margin lists the SNP for which variation is predicted to influence binding. Blue font denotes binding sites for which the risk variant abrogates a match to the binding site, while red font denotes binding sites for which the risk variant engenders a match to the binding site. **(B)** Sequence logo for Fos|SWGNSNSMDG. The black arrow denotes the position for which variation at rs6687168 is predicted to influence binding (see parts **D** and **E**). **(C)** Sequence logo for CREL|BGGNNTTTCC|M00053. The black arrow denotes the position for which variation at rs6661746 is predicted to influence binding. **(D)** Top 10 binding sites matching the non-risk variant at rs6687168 (Chr 1, 8268893 – 8268933). **(E)** Top 10 binding sites matching the risk variant at rs6687168 (Chr 1, 8268893 – 8268933). In both **(D)** and **(E)**, the right margin lists p-values quantifying the degree to which motifs are enriched among sequences adjacent to *TNFRSF9*-co-expressed genes in neutrophils.

We assembled a dictionary of 1937 empirically-determined DNA motifs recognized by human TFs and other DNA-binding proteins (see Methods). We then assessed whether any of these motifs were enriched with respect to intergenic sequences adjacent to *TNFRSF9* and those genes co-expressed with *TNFRSF9* in neutrophils. We identified 91 genes co-expressed with *TNFRSF9* in neutrophils, based upon a rank analysis of Spearman correlation coefficients (Figure 7B and D). The gene most strongly correlated with *TNFRSF9* expression, for instance, was vav 1 guanine nucleotide exchange factor (*VAV1*) (*r*_*s*_ = 0.64; Figure 7C). We next identified 595 motifs that occurred at significantly elevated frequency among intergenic regions adjacent to *TNFRSF9* and its co-expression partners in neutrophils (FDR < 0.05 with *Z* > 0). We reasoned that binding of such motifs may be disrupted by susceptibility-associated variation near rs11121129. We identified five SNPs linked with rs11121129 (*r*^2^ > 0.90), yielding a total of 6 potentially causal SNPs, and we identified cases in which these SNPs altered sequences matching those motifs enriched with respect to genes co-expressed with *TNFRSF9* in neutrophils (Figure 8A).

The risk allele at rs6687168 (265.7 kb upstream of *TNFRSF9*) abrogated a c-Fos (AP-1) binding site (Figure 8B, D and E) that was enriched among intergenic sequences adjacent to *TNFRSF9*-co-expressed genes (P = 3.4 × 10^−15^; Figure 8A). Notably, a risk allele at rs6661746 (265.4 kb upstream of *TNFRSF9*) also abrogated an NF-κB binding site enriched among intergenic sequences adjacent to *TNFRSF9*-co-expressed genes, although the degree of enrichment was weaker (P = 7.8 × 10^−9^; Figure 8A). These results suggest the hypothesis that AP-1 binds the C allele at rs6687168, leading to a psoriasis-protective effect via the effect of this interaction on *TNFRSF9* expression in neutrophils; whereas AP-1 does not bind the T allele at rs6687168, leading to increased psoriasis risk.

### Susceptibility-associated variation alters binding sites (AP-1, IRF1, STAT3 and STAT4) enriched in sequences near candidate genes and their co-expression partners

We repeated the analysis described above but focused on other intergenic loci identified from a recent GWAS meta-analysis of psoriasis (Additional file 16) [26]. Our analysis included a chromosome 9 SNP (rs10979182) within a “gene desert”, 565 kb upstream of *KLF4*, which encodes a TF known to control KC differentiation. Consistent with this, *KLF4* was most strongly expressed in KCs as compared to other cell types (Additional file 16, Part E). With respect to KCs, we identified an AP-1 motif enriched with respect to sequences near genes co-expressed with *KLF4* (P = 2.3 × 10^−3^), and found that a linked psoriasis risk variant at rs7850481 (543 kb upstream of *KLF4*) abrogated a match to this motif (Additional file 16, Part E). On the other hand, the risk variant 72 kb upstream of *IL12B* (rs12188300) engendered a motif recognized by AP-1, which was associated with *IL12B*-co-expressed genes in dendritic cells (Additional file 16, Part C). These results, taken together, suggest that AP-1:DNA interactions are sensitive to susceptibility-associated genetic variation, potentially impacting expression of candidate genes in both KCs and immune cells.

*B3GNT2* encodes a type II transmembrane enzyme located 99.6 kb from an intergenic susceptibility locus on chromosome 2 (rs10865331). Expression of *B3GNT2* is ubiquitous with detection frequency greater than 99% for each of the 10 cell types we considered; however, expression was quantitatively higher in macrophages as compared to other cell types (Additional file 16, Part B). Genes co-expressed with *B3GNT2* in macrophages were located next to intergenic sequences with elevated density of motifs recognized by TFs important to psoriasis pathogenesis, such as IRF1, STAT4 and STAT5 (Additional file 16, Part B). With respect to the IRF1 motif, a putative binding site is sensitive to variation at rs6759003 (107 kb from the *B3GNT2* transcription stop site), with the risk allele predicted to increase IRF1 affinity (Additional file 16, Part B). Similarly, we identified a second locus at rs13024541 (104 kb from *B3GNT2*) for which the risk variant increased affinity for STAT TFs enriched in sequences near *B3GNT2*-co-expressed genes in macrophages, including STAT4, STAT5A and STAT6 (Additional file 16, Part B). These results are consistent with the hypothesis that susceptibility-associated variation near *B3GNT2* leads to increased IRF1 and STAT1 binding to modify *B3GNT2* expression in macrophages.

## Discussion

Genome-scale studies of psoriasis have identified many genes involved in the disease process, but further work is needed to understand how each gene contributes to lesion development. The main goal of this study, therefore, was to extend findings from expression profiling and GWAS studies of psoriasis by establishing connections between individual genes and candidate cell types. We estimate that 50% of genes differentially expressed in psoriasis lesions (DEGs) can be attributed to KCs or fibroblasts, with the other 50% driven by the activities of immune cells. This contrasted with genes implicated in psoriasis GWAS studies, which were less frequently assigned to KCs or fibroblasts (20%), but were more commonly assigned to immune cells (71%) with many showing highest expression in neutrophils (24%). This latter result emphasizes the role of the innate immune system in psoriasis, consistent with novel loci uncovered in a recent psoriasis GWAS meta-analysis [26]. The identification of candidate cell types provides an important first step towards functional analysis, and to illustrate this idea we developed a systematic approach for generating hypotheses to explain GWAS hits at intergenic loci. This allowed us to identify (non-coding) susceptibility-associated variation predicted to influence binding of TFs associated with co-expression networks in specific cell types. Our findings suggest new mechanisms by which non-coding susceptibility alleles might regulate expression of nearby target genes, including mechanisms mediated by the binding of sequence-specific factors such as AP-1, NF-κB, IRF1, STAT3 and STAT4.

It has often been assumed that differential expression trends in psoriasis lesions are driven mostly by KCs, which are the majority cell type in full-thickness skin biopsies. This expectation is more likely correct for genes with significantly elevated expression in psoriasis lesions, but is less likely correct for genes with decreased expression. Among DEGs with decreased expression in lesions, we assigned twice as many to fibroblasts (33%) as to KCs (15%) (Figure 2). This result is consistent with previous findings [3,6,42] and may be attributed to several mechanisms. First, angiogenesis within psoriasis lesions expands the dermal vasculature [43], and this may decrease the number of fibroblasts and consequently the abundance of fibroblast-derived mRNAs. Second, it is possible that epidermal expansion in psoriasis lesions leads to increased expression of KC-derived transcripts and that this effectively dilutes transcripts derived from minority cell types, such as fibroblasts [42]. It is notable that neither of these possibilities requires any shift in transcription on a per-cell basis in fibroblasts. Nevertheless, changes in the transcriptional activity of fibroblasts do occur and are likely to shape the transcriptome of full-thickness lesional skin biopsies to some degree. Psoriatic fibroblasts, for example, produce excessive amounts of cytokines such as IL-6 and IL-8 [44,45], and it has been proposed that these and other fibroblast-derived factors contribute to angiogenesis and also control KC proliferation and differentiation [46]. Further work will thus be needed to better understand why fibroblast-derived mRNAs are so abundant among psoriasis-decreased DEGs, and in this regard, we expect that analyses of dermal and epidermal sections of lesions obtained by laser capture microdissection will prove informative [47]. Understanding the contribution of fibroblasts to the psoriasis transcriptome will likely have implications for the interpretation of genome-wide expression patterns in other skin diseases as well, since we observed strong overlap between psoriasis DEGs and those genes with altered expression in other skin diseases, such as squamous cell carcinoma, atopic dermatitis and Mediterranean spotted fever eschars (Figure 1 and Additional file 4).

Development of an inflammatory infiltrate is a characteristic feature of psoriasis lesions, although even macroscopically normal skin includes a sizable immune cell population [48]. Psoriasis-increased DEGs included a slightly higher-than-expected number of genes assigned to macrophages (Figure 2) and the most strongly elevated DEGs (>2-fold) included a significant proportion of genes most highly expressed by neutrophils (data not shown). We could also identify some psoriasis-decreased DEGs expressed mostly by immune cells (Figure 4), possibly representing cases in which the altered environment of psoriasis lesions led to down-regulation of transcription within resident (non-infiltrating) immune cells. Overall, the proportion of psoriasis DEGs associated with T-cells, NK-cells, B-cells, DCs and monocytes was limited (Figure 2). This is most likely due to the variable degree of inflammatory cell infiltration that occurs among lesions, which may be associated with variation among lesions with respect to developmental stage, genetic background, or environmental factors (Additional file 9) [3,6,49]. As a result of this variation, genes associated with infiltration by these cell types may be excluded from DEGs, which instead include genes showing consistent responses across lesions (e.g., KCs and fibroblasts). This suggests that, in order to study inflammatory cell infiltration in psoriasis lesions, large sample sizes are generally needed, and a strict focus on genes meeting criteria for differential expression is insufficient [3,6]. It is interesting to note that the most consistent inflammatory signature was observed for neutrophils (Additional file 9). Among lesions from the 216 patients, we almost always observed a slight increase in the expression of neutrophil signature genes; moreover, in contrast to other immune cell types, we never observed patients showing a significant decrease in the expression of neutrophil signature genes (Additional file 9).

Neutrophils are a hallmark feature of psoriasis lesions and are located in concentrated areas under the stratum corneum where they form regions known as Munro’s microabscesses [24]. In our analysis, there was significant enrichment of neutrophil-assigned genes among GWAS candidates, with 1 of every 4 candidates expressed more highly in neutrophils than any other cell type (Figure 2). Whether neutrophils have a causative role in the development of psoriasis lesions remains unclear, but their appearance in lesions precedes that of T-cells [49], and regions near neutrophil microabscesses show heightened mitosis and epidermal growth [24,50]. Prior studies have also demonstrated a correlation between lesion development and abundance of neutrophils in circulation [51,52], and reduction of peripheral neutrophils using adsorption apheresis was reported to improve psoriasis lesions in 39 of 44 patients [53]. Neutrophils also represent a source of cytokines demonstrated to drive lesion development, such as IL-17A, and indeed one study indicated that IL-17A(+) neutrophils are more abundant in lesions than IL-17A(+) T-cells [54]. Such epidermal neutrophils generate reactive oxygen species such as superoxide anion, leading to tissue damage and possible hyper-activation of innate defense mechanisms [55]. Neutrophils also interact with other cell types, such as KCs, T-cells and dendritic cells. For example, KCs can be induced to express cell surface molecules, such as the adhesion glycoprotein CEACAM1, which facilitate KC-neutrophil interactions and prolong lifespan of neutrophils once removed from circulation [56]. Under certain conditions, moreover, neutrophils express MHC class II molecules such as HLA-DR, which may allow neutrophils to interact directly with T-cells and participate in antigen presentation and T-cell activation [24,57-59]. Post-apoptotic neutrophils within the epidermis may also provide a source of autoantigens by the formation of neutrophil extracellular traps (NETs) consisting of a web-like chromatin structure, human neutrophil elastase (HNE), cathelicidin (LL37), and secretory leukocyte proteinase inhibitor (SLPI) [60-62]. These NET components provide DNA complexes that can enhance pDC activation and production of cytokines such as IFN-α [60].

Genome-scale studies of psoriasis are exploratory and not hypothesis-driven, but such studies can provide the leads necessary to begin hypothesis-driven investigations into pathogenetic mechanisms [1,2]. We and others have emphasized that identification of candidate cell types is necessary as a first step towards functional analysis of individual genes [7]. Along these lines, one starting point may be the limited set of 15 genes implicated from GWAS results that are also included among DEGs with altered expression in psoriasis lesions (Figure 5). Of these 15 DEGs, two attractive candidates are *LCE3D* and *GRHL3*, since both genes show a trend towards cell type-specific expression, with strong expression in KCs but lacking detectable expression in other cell types (Figure 5). Consistent with KC-specific expression, *LCE3D* encodes a cornified envelope protein [63] and *GRHL3* encodes a TF that activates a pro-differentiation gene expression program [35]. Among the 13 other DEGs associated with psoriasis susceptibility loci, most are expressed by multiple cell types (Figure 5). Of these, however, 5 were expressed more highly in neutrophils than any other cell type (*IFIH1*, *PPIF*, *IL12B*, *DDX58* and *STAT3*). Interestingly, *IFIH1* (also known as *MDA5*) and *DDX58* (also known as *RIG-I*) encode (RIG-I-like) receptors that recognize virus-derived intracellular DNA and thus serve as a part of the cytosolic trigger for activation of innate immune responses [64]. Recently, this viral response pathway emerged from a large-scale analysis of expression data, where it was designated as the “STAT1-57” regulon and shown to be activated in psoriasis lesions, cytokine-inducible (e.g., IFN-γ, IFN-α, TNF, IL-1α), and repressed *in vivo* by biologic therapies or glucocorticoids [65]. *IFIH1* and *DDX58* are expressed in human KCs where they function as RIG-I-like receptors to initiate antiviral defenses [66,67]. These receptors, however, have similar functionality in neutrophils, suggesting that this same antiviral pathway might be activated in multiple cell types from the psoriatic epidermis [68].

The majority of signals identified in psoriasis GWAS studies are located in non-coding regions and this has also been the case for other complex genetic diseases [26,32,69,70]. Susceptibility-associated variation at non-coding loci may influence gene expression by altering TF binding, but it is time-consuming to test hypotheses regarding the effects of individual SNPs on TF binding and downstream expression [39-41]. We therefore developed an approach to prioritize candidate regulatory mechanisms, which provides a rank-ordering of potentially causal non-coding SNPs at which susceptibility-associated variation is predicted to influence TF binding in specific cell types. We applied this strategy to six intergenic susceptibility loci identified from a recent psoriasis GWAS meta-analysis (Additional file 16) [26]. We identified SNPs near *TNFRSF9* at which psoriasis susceptibility variants disrupted binding sites for AP-1 (rs6687168) and NF-κB (rs6661746), where both binding sites were also enriched with respect to sequences adjacent to genes co-expressed with *TNFRSF9* in neutrophils (Figures 7 and 8). In T-cells, a prior study has confirmed that AP-1 and NF-κB positively regulate *TNFRSF9* expression [71,72], although it is unclear whether such regulation occurs in neutrophils or other immune cell types. *TNFRSF9* (also *CD137* or *4-1BB*) encodes a TNF receptor superfamily membrane protein that mediates T-cell activation [73], but *TNFRSF9* is also expressed by other immune cells including neutrophils [74]. Within psoriasis lesions, *TNFRS9* expression on neutrophils may have pro- and anti-inflammatory effects. On the one hand, expression of *TNFRS9* inhibits extension of neutrophil lifespan mediated by the anti-apoptotic effects of cytokines such as IFN-γ, and this can increase neutrophil apoptosis and reduce cytotoxic damage associated with neutrophil infiltration [75,76]. These same effects, however, may favor the formation of NETs, providing a source of autoantigens and contributing to pDC activation [60]. Loss of *TNFRSF9* expression due to psoriasis susceptibility alleles may thus have either pro- or anti-inflammatory effects by altering the balance of forces affecting neutrophil apoptosis.

Psoriasis is now understood to be an (auto)-immune disease that is dependent upon interactions between KCs and immune cells [2,9,13]. The exact cell type underlying the primary defect leading to development of psoriasis lesions has been extensively debated, and at a glance, findings from psoriasis GWAS studies have been mixed, with some loci seeming to support a primary role for KCs and others pointing towards innate or adaptive immune cells [9,11,14]. The analysis of gene expression alone is not sufficient to resolve this issue, but quantitative analyses of gene expression can at least provide objective insights [7]. Our analysis of genes near susceptibility loci from psoriasis GWAS studies lends support to multiple cell types, including T-cells, but above all our findings bolster support for neutrophils as participants in pathogenetic disease mechanisms [24,49,54,55,60]. We expect that this conclusion can be sharpened by future work, based upon the identification of new psoriasis susceptibility loci, new experimental evidence, generation of new datasets for evaluating cell type-specific expression, and continued development of bioinformatic analysis protocols. This ongoing work will contribute to the broader goal of finding connections between individual genes and their disease-relevant cell types [7]. This gene-cell mapping will, in turn, be essential for the design of hypothesis-driven functional studies, which will be needed to fully integrate findings from GWAS studies into our understanding of psoriasis disease mechanisms.

## Conclusions

The purpose of this study was to extend findings from expression profiling and GWAS studies by mapping identified genes to candidate cell types. Our findings indicate that approximately 50% of psoriasis DEGs can be attributed to resident skin cells, although the dominant cell type differs between psoriasis-increased DEGs (keratinocytes, 35%) and psoriasis-decreased DEGs (fibroblasts, 33%). In contrast, genes located near susceptibility loci from psoriasis GWAS studies are predominantly associated with immune cells (71%), and for such genes there is a significant tendency for expression to be highest in neutrophils (24%, P = 1.5 × 10^−5^). We have also investigated GWAS hits at (non-coding) intergenic psoriasis susceptibility loci to identify sites at which allele-specific TF binding may influence candidate gene expression. Our results suggest new hypotheses for interpretation of intergenic hits from psoriasis GWAS studies, and we have identified SNPs predicted to influence binding of AP-1, NF-κB, IRF1, STAT3 and STAT4. Taken together, our findings offer objective and data-driven evidence supporting the idea that innate immunity and neutrophils contribute to psoriasis pathogenesis. Additionally, by mapping genes to cell types, we have taken steps towards post-GWAS functional analysis of psoriasis susceptibility loci, and for the first time we have implemented a strategy for interpretation of GWAS hits within intergenic (non-coding) regions.

## Methods

### Ethics statement

Skin biopsies were obtained from volunteer patients in accordance with Declaration of Helsinki principles [6,27-31]. Informed written consent was provided by each participant under protocols approved by an independent ethics committee or institutional review board (Alfred Hospital, Melbourne, Australia; Chesapeake Research Review Inc., Columbia, MD; Research Review Board Inc., Richmond Hill, Ontario, Canada; Rockefeller University, New York, NY, IRB no. AMA-0674; Royal Adelaide Hospital, Adelaide, Australia; University of Michigan, Ann Arbor, MI, IRB No. HUM00037994).

### Microarray analysis of psoriasis gene expression data

Data were pooled from 8 studies that had used microarrays to evaluate gene expression in lesional and uninvolved skin from psoriasis patients (Additional file 17; Gene Expression Omnibus accession IDs: GSE13355, GSE14905, GSE30999, GSE34248, GSE41662, GSE41663, GSE47751 and GSE50790) [6,27-31]. The same commercial oligonucleotide microarray platform was used in each study (Affymetrix Human Genome U133 Plus 2.0 array). The initial dataset included 458 paired PP and PN samples from 229 patients. CEL data files were evaluated using Affymetrix quality control (QC) metrics, such as average background, scale intensity factor, RNA degradation score, percentage of probe sets called present, and four metrics derived from probe level models (NUSE median, NUSE IQR, RLE median, RLE IQR) (Additional file 18, Parts A – H) [77]. A sample was removed if it was extreme with respect to the complete dataset or other samples from the same study (since data from each study was normalized independently, see below). This ensured that all samples normalized together could be compared under viable normalization assumptions. After QC filtering, there remained 434 paired PP and PN samples from 217 patients.

Post-QC samples were normalized using Robust Multichip Average (RMA). Samples from GSE14905, GSE34248, GSE41662, GSE41663, GSE47751 and GSE50790 were each normalized independently using RMA. For GSE13355, samples had been processed in three batches over three consecutive years, but PP and PN samples from the same patient were always allocated to the same batch. The three batches of samples were therefore normalized (by RMA) independently of one another. Similarly, for GSE30999, samples had been collected at 10 study sites, with PP and PN samples from the same patient always collected at the same site. GSE30999 samples were thus normalized in 10 different batches, with each batch including only samples from the same study site. Given these normalization procedures, samples from each dataset and/or batch were normalized separately, but in every case PP and PN samples from the same patient were normalized together. After normalization, PP versus PN differences in RMA expression scores were calculated for each probe set and each patient, and these differences were pooled across all batches and all studies.

Affymetrix arrays feature multiple “sibling” probe sets designed to target the same gene [78]. To limit redundancy, we *a priori* chose a single probe set to evaluate for each of the 20184 human genes represented on the Affymetrix Human Genome U133 Plus 2.0 array. In choosing the representative, we preferentially selected probe sets expected to hybridize specifically with its target, excluding those probe sets containing either an “_s_” or “_x_” suffix in the Affymetrix probe set identification number [79]. If there was more than one probe set representing the same gene, but no difference in expected hybridization specificity, the representative probe set was chosen as the one for which median RMA expression was the highest across all 434 PP and PN samples. For each probe set, we calculated the median RMA expression score among PP samples, the median RMA expression score among PN samples, averaged these two values, and chose as a representative the probe set for which this average value was greatest. For 15175 genes (75.2%), the chosen representative probe set was expected to hybridize specifically with a single mRNA transcript from the target gene. For nearly all other genes (21.8%), the representative was expected to hybridize non-specifically but only with mRNAs derived from the same human gene (i.e., probe sets with an “_s_” suffix) [79].

We next inspected samples to assess whether genes characteristically increased or decreased in psoriasis lesions showed expected trends (Additional file 18, Part I). This revealed one outlier patient for which genes typically increased in PP skin were decreased, while conversely, genes typically decreased in PP skin were increased (GSE30999; samples GSM768097 and GSM768096). Most likely, for this patient, PP and PN labels had been reversed during sample processing in the laboratory. Nevertheless, since it was not possible to confirm the labeling error, samples from this patient were excluded. Following these pre-processing steps, there remained 432 paired PP and PN samples from 216 patients, with samples from all patients showing expected trends for genes characteristically increased or decreased in lesions (Additional file 18, Part I). Finally, the 216 patients were clustered based upon PP versus PN differences in RMA expression scores across 16358 skin-expressed genes (Additional file 18, Part J). Cluster analysis did not identify strong outliers and further showed that patients did not cluster primarily according to the dataset from which patient samples were derived (Additional file 18, Part J). Consistent with prior work [30], this indicated that variation among the 216 patients is only weakly associated with study-specific factors.

Differential expression analyses were carried out with respect to 16358 skin-expressed genes. We here consider skin-expressed genes to be those for which expression was detected for at least 10% of the 432 biopsies (PP and PN) from the 216 patients. Detection of representative probe sets was assessed by testing for a difference in signal intensities between paired perfect match (PM) and mismatch (MM) probes, with a significant difference providing evidence for detection of a probe set above background (P < 0.05; one-sided Wilcoxon signed-rank test) [38]. For the 16358 skin-expressed genes, a non-parametric method was used to assess differential expression between paired PP and PN biopsies (Wilcoxon rank sum test; *n* = 216 patients). To control the false discovery rate (FDR), raw p-values were adjusted using the Benjamini-Hochberg correction [80]. For identification of differentially expressed genes (DEGs), we required FDR < 0.05 along with median PP/PN fold-change (FC) greater than 1.50 or less than 0.67. In addition, PP-increased DEGs included only those genes for which median FC was greater than 1 with respect to each of the 8 studies included in our analysis. Similarly, PP-decreased DEGs included only genes for which median FC was less than 1 with respect to each of the 8 studies. This ensured that DEGs showed consistent trends across all studies included in the analysis. These criteria yielded 1019 PP-increased DEGs (FC > 1.50 and FDR < 0.05) along with 885 PP-decreased DEGs (FC < 0.67 and FDR < 0.05).

### Gene expression across 10 cell types (keratinocytes, fibroblasts, CD4+ T-cell, NK cell, CD8+ T-cell, B-cell, macrophages, monocytes, dendritic cells, neutrophils)

We used microarray data from Gene Expression Omnibus to construct a database for evaluation of gene detection and expression across 10 disease-relevant cell types (Additional file 5). Data samples were generated from the same microarray platform used to profile expression in lesional and uninvolved skin from psoriasis patients (Affymetrix Human Genome U133 Plus 2.0 array). For each cell type, an initial batch of CEL files was downloaded and screened to filter out low-quality hybridizations. We removed 5% of samples with extreme background (2.5% lowest and 2.5% highest), 5% of samples with extreme scale parameters (2.5% lowest and 2.5% highest), 5% of samples with the fewest probe sets called present, and 5% of samples with high RNA degradation scores (Additional file 5). Based on these QC criteria, we excluded 11 – 15% of CEL files initially identified for each cell type (Additional file 5). A representative probe set was evaluated for each human gene represented on the array, and for consistency, we used the same representatives as in our analysis of differential expression in PP and PN skin (see above). Similarly, as above, a gene was considered detected in a sample if there was a significant difference in signal intensities between PM and MM probes (one-sided Wilcoxon signed-rank test) [38]. A gene’s overall detection frequency for a cell type was thus calculated as the proportion of post-QC samples for which this test was significant (P < 0.05).

Relative expression across cell types was evaluated based upon 50 representative microarray samples chosen for each cell type. To identify these samples, all post-QC samples for a given cell type were normalized by RMA and we calculated a centroid by averaging expression for each gene across samples. The 50 representative samples were then chosen by identifying those for which the Euclidean distance between the sample and cell type centroid was minimal. After the 50 representative samples had been identified for each cell type, representatives from all cell types were normalized together by RMA to create a composite dataset. For normalization purposes, the composite dataset also included 50 representative microarray samples used to evaluate expression in normal human skin (i.e., representatives chosen from 131 post-QC normal skin samples). In total, therefore, the composite dataset consisted of 550 microarray samples, including representatives from 10 cell types in addition to normal human skin. These data were used to compare relative expression across cell types, with expression values for each gene normalized to its median expression in normal human skin.

### Identification of genes associated with psoriasis in genetic studies

We identified 117 candidate genes from psoriasis GWAS studies based upon entries from the National Human Genome Research Institute (NHGRI) GWAS catalogue [32] and a recent psoriasis GWAS meta-analysis [26].

We first identified 44 entries associated with the disease trait “Psoriasis” in the NHGRI GWAS catalogue [32]. Genes associated with these entries included those reportedly associated with each SNP in the source study as well as any genes immediately upstream or downstream (i.e., database columns “Reported Genes”, “Upstream_gene_id” and “Downstream_gene_id”). Additionally, we analyzed the chromosomal location for each entry and identified any genes overlapping with this location, the most immediate upstream gene, and the most immediate downstream gene. Altogether, we identified 77 unique candidate genes associated with 44 entries from the NHGRI GWAS catalogue [32].

Second, we analyzed 36 loci identified from a recent psoriasis GWAS meta-analysis [26]. Candidate genes associated with these loci included the “notable genes” listed in Table one from Tsoi et al. [26], any genes overlapping the lead SNP, and genes immediately upstream or downstream from the lead SNP. This yielded 101 unique candidate genes associated with 36 loci.

We pooled the 77 candidates identified from the NHGRI GWAS catalogue with the 101 candidates identified in the GWAS meta-analysis. After removing duplicates from both sources, there remained 129 unique genes. Of these, 117 were represented by a probe set feature on the Affymetrix Human Genome U133 Plus 2.0 array and were thus considered in our analyses of psoriasis GWAS candidates.

Additional analyses were performed based upon 357 genes included in the Human Genome Epidemiology Network (HuGENet) database (Additional files 13 and 14) [33]. This gene set is more comprehensive than that discussed above, since it includes genes associated with psoriasis in GWAS studies, as well as genes linked to psoriasis in non-GWAS epidemiologic population-based studies [33]. The “Phenopedia” file was downloaded from the HuGENet website (October 2013) and we identified 395 unique human genes associated with the disease identifier “Psoriasis(C0033860)”. Of these 395 genes, 357 were represented by a probe set feature included on the Affymetrix Human Genome U133 Plus 2.0 array and were thus included in our analyses (Additional files 13 and 14).

### Analysis of susceptibility-associated variation near intergenic (non-coding) psoriasis susceptibility loci

We analyzed susceptibility-associated variation near six intergenic (non-coding) susceptibility loci identified from a recent psoriasis GWAS meta-analysis (Figures 7 and 8 and Additional file 16) [26]. In each case, we first identified a suspected target gene near the susceptibility locus (i.e., *TNFRSF9*, *B3GNT2*, *IL12B*, *TAGAP*, *KLF4* or *NFKBIA*). The cell type for which the target gene’s median expression was highest was assigned as the candidate cell type (Figure 7A). Given this cell type, we identified the local co-expression network for the target gene, consisting of the *N* genes most strongly co-expressed with the target gene (Spearman correlation coefficient, *r*_s_). The cut-off value of *N* was chosen based upon the steepness with which *r*_s_ estimates declined across the set of genes (with *r*_s_ > 0) that had been ranked in descending order according to *r*_s_ estimates (Figure 7B). The chosen value of *N* was determined by identifying the bending point or “knee” in this curve, corresponding the point at which distance between the curve and the origin of the plot is minimized (Figure 7B) [81,82]. Once the local co-expression network for the suspected target gene had been identified, intergenic sequences adjacent to genes within this co-expression network were analyzed to identify DNA motifs present with significantly elevated frequency [81].

We compiled a dictionary of 1937 motifs, where each motif represented the experimentally-determined binding preference of a human TF or DNA-binding protein. Motifs were obtained by pooling collections from five sources (i.e., Jaspar, UniPROBE, ENCODE, hPDI and TRANSFAC) [83-87], as described in a recent publication [65]. To identify motif matches in the human genome, intergenic sequences were scanned with masking of repetitive DNA elements and assembly gaps (R package: BSgenome.Hsapiens.UCSC.hg19). A position weight matrix (PWM) was evaluated for each motif, with a genome match declared at any locus for which the PWM score exceeded 80% of the maximum match score for that PWM (R function: matchPWM, R package: Biostrings). PWM matches were assessed using empirically-determined nucleotide background frequencies estimated from intergenic regions in the human genome (0.30, 0.20, 0.20, 0.30 for A, C, G and T, respectively). For each intergenic sequence, forward and reverse strands were scanned in 5’ to 3’ orientation, and the total number of PWM matches for any sequence was equal to the number of matches on both strands (any overlapping matches were merged and not double-counted). For each motif, the number of matches was compared between a foreground set of intergenic sequences (adjacent to the *N* genes within the co-expression network) and a background set of intergenic sequences (adjacent to genes with detectable expression in the candidate cell type excluding sequences adjacent to the *N* genes within the co-expression network). Significant enrichment with respect to foreground sequences was assessed using semiparametric generalized additive logistic models [81]. Based on this analysis, we identified motifs significantly associated with co-expression networks for the putative target gene and ranked all motifs according to the strength of this association.

For each of the six intergenic loci, we identified SNPs in linkage disequilibrium with the lead SNP identified in the prior GWAS meta-analysis (*r*^2^ > 0.90) [26]. To identify linked SNPs, variant call format (vcf) files were downloaded from the 1000 Genomes project (phase 1) and converted to PLINK input files (EUR subpopulation). PLINK was then used to identify all SNPs in linkage disequilibrium with the lead SNP (*r*^2^ > 0.90) (i.e., the --ld-snp command option) [88]. We inspected linked SNPs to identify those at which variation engendered or abrogated a match to one of the 1937 motifs included in our dictionary. Consistent with the above-stated criterion, a motif match for a SNP locus was called if the PWM score exceeded 80% of the maximum score for that PWM on either the forward or reverse strand (R function: matchPWM, R package: Biostrings). This yielded SNP-motif pairs for which genetic variation was predicted to influence TF binding. SNP-motif pairs were then ranked by the degree to which the motif showed enrichment among sequences adjacent to genes belonging to the target gene’s co-expression network (Figure 8A).

### Analysis of *TNFRSF9* mRNA and protein expression

Human KCs and immune cell subsets were obtained from healthy human volunteers using a published protocol [89]. Lesional, uninvolved and healthy control skin was obtained and processed as described [90]. RNA was extracted using RNeasy columns (Qiagen, Cat No. 74104) and evaluated using the Agilent 2100 Bioanalyzer. A commercial kit was used for reverse transcription (Applied Biosystems, Cat No. 4368814). QRT-PCR was done with primers for *TNFRSF9* (Applied Biosystems, Cat No. Hs00155512_m1) and *RPLP0* (Applied Biosystems, Cat No. Hs99999902_m1) using the 7990HT Fast Real-Time PCR system (Applied Biosystems). Whole blood was obtained from healthy donors and analyzed by flow cytometry for cell surface TNFRSF9 (Biolegend, Cat. No. 309810), anti-CD15 (BioLegend, Cat No. 323022), anti-CD3 (BioLegend, Cat No. 344808) and anti-CD14 (BD Pharmingen, Cat. No. 555397). Samples were analyzed using the LSR II flow cytometer (BD Biosciences).

## Competing interests

The authors declare that they have no competing interests.

## Authors’ contributions

WRS, PS, AJ and JEG participated in the design of the study, analysis of data and drafting the manuscript. MKS performed RT-PCR and flow cytometry experiments. JJV and JTE assisted in drafting the manuscript and revising it critically. All authors have read and approved of the final manuscript.

## Pre-publication history

The pre-publication history for this paper can be accessed here:

http://www.biomedcentral.com/1755-8794/7/27/prepub

## Supplementary Material

Additional file 1 **Genes significantly elevated in psoriasis lesions (*n*** **= 216 patients).** The table lists the 1019 differentially expressed genes (DEGs) significantly elevated in psoriasis lesions (FC > 1.50, FDR < 0.05). DEGs are sorted according to their median fold-change in lesional skin as compared to uninvolved skin (*n* = 216 patients). The table lists the frequency with which each gene was detected significantly above background among PP samples (third column) and PN samples (fourth column) (Wilcoxon signed rank test, P < 0.05). P-values for differential expression were calculated using the Wilcoxon rank sum test (fifth column) and FDR-adjusted p-values were calculated using the Benjamini-Hochberg method (final column). Click here for file

Additional file 2 **Genes significantly decreased in psoriasis lesions (*n*** **= 216 patients).** The table lists the 885 differentially expressed genes (DEGs) significantly decreased in psoriasis lesions (FC < 0.67, FDR < 0.05). DEGs are sorted according to their median fold-change in lesional skin as compared to uninvolved skin (*n* = 216 patients). The table lists the frequency with which each gene was detected significantly above background among PP samples (third column) and PN samples (fourth column) (Wilcoxon signed rank test, P < 0.05). P-values for differential expression were calculated using the Wilcoxon rank sum test (fifth column) and FDR-adjusted p-values were calculated using the Benjamini-Hochberg method (final column). Click here for file

Additional file 3 **Psoriasis DEGs are (on average) detected in most (>84%) PP and PN skin samples (P ≤ 0.02).** Figures (A) – (D) show the average detection frequency of psoriasis DEGs (black arrow) as compared to a null distribution generated by random sampling from all skin-expressed genes. (A) The 1019 PP-increased DEGs were detected in 94.5% of PP samples on average. This percentage value was significantly larger than expected based upon 10000 random samples of 1019 genes. (B) The 1019 PP-increased DEGs were detected in 84.1% of PN samples on average (P = 0.02, 10000 random samples of 1019 genes). (C) The 885 PP-decreased DEGs were detected in 86.5% of PP samples on average (P < 0.001, 10000 random samples of 885 genes). (D) The 885 PP-decreased DEGs were detected in 94.5% of PN samples on average (P < 0.001, 10000 random samples of 885 genes). Click here for file

Additional file 4 **Gene set enrichment analysis: Psoriasis DEGs overlap significantly with genes altered in various skin conditions, cytokine-treated KCs, and RNAi-treated KCs.** (A) Comparison with skin diseases. Expanded results from Figure 1A with the top-ranked 30 comparisons (of 51 included in our screen). (B) Comparison with cytokine-treated KCs. Expanded results from Figure 1B with the top-ranked 30 comparisons (of 46 included in our screen). (C) RNAi experiments. Ranked gene lists were generated from experiments in which microarrays were used to evaluate gene expression in KCs following RNAi. The RNAi target is listed in the label for each experiment (left margin). In both (B) and (C), one asterisk (*) denotes experiments performed with HaCaT KCs, while two asterisks (**) denotes experiments performed using reconstituted epidermis. All other experiments were performed with primary NHEKs. Click here for file

Additional file 5 **Number of microarray samples analyzed for each cell type.** The table lists the number of microarray samples initially considered for each cell type, as well as the number of microarray samples remaining following quality control checks (post-QC). All samples were generated using same oligonucleotide microarray platform (Affymetrix Human Genome U133 Plus 2.0 Array). Analyses are based only upon the post-QC samples that survived the quality control filtering (final column on right). GEO series accession ids associated with post-QC samples are listed below the table, with the number of samples from each GEO series listed in parentheses. Click here for file

Additional file 6 **Genes assigned to each of 10 cell types show bias towards PP-increased or PP-decreased expression.** (A) The 16358 skin-expressed genes were ranked according to the estimated median PP/PN fold-change (*n* = 216 patients, horizontal axis). For each cell type, the line tracks the cumulative overlap between top-ranked genes and the set of genes assigned to that cell type. The area calculated for each cell type represents the region between the diagonal line (random expectation) and the line drawn for each cell type, respectively. P-values were generated from the Wilcoxon rank sum test. (B) The same analysis was repeated, except only genes showing a cell type-specific expression pattern were included, with expression in the assigned cell type significantly higher than expression in each of the other 9 cell types (FDR < 0.05; Wilcoxon rank sum test). Click here for file

Additional file 7 **PP-increased DEGs show aberrantly high expression in KCs and macrophages.** We identified 1019 PP-increased DEGs (median FC > 1.50, FDR < 0.05; Wilcoxon rank sum test; *n* = 216 patients). The average detection frequency among the 1019 DEGs was calculated for each cell type (black arrows, left column). Likewise, the median expression among the 1019 DEGs was calculated with respect to each cell type (black arrows, right column). Null distributions for the average detection frequency and median expression level were generated based upon 10000 samples of 1019 genes chosen randomly from 16358 skin-expressed genes. P-values were calculated using these empirical null distributions and are shown in the figure. Expression values (right column) are normalized to normal human skin, such that values greater than 1 indicate that expression is greater than that in normal skin, while values less than 1 indicate that expression is lower than that in normal skin. Click here for file

Additional file 8 **Top 30 genes most strongly elevated in psoriasis lesions (*n*** **= 216 patients) and their expression across 10 cell types.** The table lists the 30 DEGs most strongly elevated in psoriasis lesions (i.e., highest median PP/PN fold-change, with FDR < 0.05). The color scale denotes median expression of each gene, as compared to the gene’s median expression in normal human skin. Red colors thus denote expression that is higher in comparison to normal human skin, while blue colors indicate that expression is lower in comparison to normal human skin. For each gene and cell type, black circles indicate the percentage of microarray samples for which the gene’s expression was detected above background (P < 0.05, Wilcoxon signed-rank test). The cell type assigned to each gene is listed in the right margin. Magenta labels denote cases in which the gene’s expression in the assigned cell type is significantly higher than its expression in any of the other 9 cell types (FDR < 0.05, Wilcoxon rank sum test). Click here for file

Additional file 9 **Psoriasis lesions from 216 patients vary with respect to the expression of genes specifically expressed by immune cell types.** Cell type-specific signature scores were calculated for each patient and each cell type. Signature scores for a cell type were calculated based upon a set of 250 genes with high expression in that cell type (as compared to the other 9 cell types), with scores equal to the weighted average of fold-change values (PP/PN) among the 250 genes for a given patient (weighted arithmetic mean). Genes showing the most cell type-specific expression pattern were assigned the greatest weight in these calculations. Patients were then clustered based upon signature scores across the 10 cell types (Euclidean distance with complete linkage). Values in the bottom margin indicate the percentage of patients for which signature scores were significantly large (red, P < 0.05) or significantly small (blue, P < 0.05). Significance of scores was evaluated based upon whether PP/PN fold-changes for the 250 signature genes differed from those of all other skin-expressed genes (Wilcoxon rank sum test). Patients were assigned to one of three groups based upon the patterns observed for immune cell types (weak inflammation, 67/216; moderate inflammation, 104/216; strong inflammation, 45/216). Click here for file

Additional file 10 **PP-decreased DEGs show aberrantly low expression in all cell types except fibroblasts.** We identified 885 PP-decreased DEGs (median FC > 1.50, FDR < 0.05; Wilcoxon rank sum test; *n* = 216 patients). The average detection frequency among the 885 DEGs was calculated for each cell type (black arrows, left column). Likewise, the median expression among the 885 DEGs was calculated with respect to each cell type (black arrows, right column). Null distributions for the average detection frequency and median expression level were generated based upon 10000 samples of 885 genes chosen randomly from 16358 skin-expressed genes. P-values were calculated using these empirical null distributions and are shown in the figure. Expression values (right column) are normalized to normal human skin, such that values greater than 1 indicate that expression is greater than that in normal skin, while values less than 1 indicate that expression is lower than that in normal skin. Click here for file

Additional file 11 **Top 30 genes most strongly decreased in psoriasis lesions (*n*** **= 216 patients) and their expression across 10 cell types.** The table lists the 30 DEGs most strongly decreased in psoriasis lesions (i.e., lowest median PP/PN fold-change, with FDR < 0.05). The color scale denotes median expression of each gene, as compared to the gene’s median expression in normal human skin. Red colors thus denote expression that is high in comparison to normal human skin, while blue colors indicate that expression is low in comparison to normal human skin. For each gene and cell type, black circles indicate the percentage of microarray samples for which the gene’s expression was detected above background (Wilcoxon signed-rank test; P < 0.05). The cell type assigned to each gene is listed in the right margin. This is the cell type for which the gene’s median expression level was the highest, provided that the gene’s detection frequency for that cell type was greater than 10%. Magenta labels denote cases in which the gene’s expression in the assigned cell type is significantly higher than its expression in each of the other 9 cell types, respectively (FDR < 0.05, Wilcoxon rank sum test). Click here for file

Additional file 12 **Candidate genes from psoriasis GWAS studies show aberrantly high expression in immune cell populations.** We identified 117 candidate genes near GWAS loci. The average detection frequency among these 117 genes was calculated for each cell type (black arrows, left column). Likewise, the median expression among the 117 genes was calculated with respect to each cell type (black arrows, right column). Null distributions for the average detection frequency and median expression level were generated based upon 10000 samples of 117 genes chosen randomly from the 20184 human genes represented on the Affymetrix Human Genome U133 Plus 2.0 array. P-values were calculated using these empirical null distributions and are shown in the figure. Expression values (right column) are normalized to normal human skin, such that values greater than 1 indicate that expression is greater than that in normal skin, while values less than 1 indicate that expression is lower than that in normal skin. Click here for file

Additional file 13 **Assignment of gene candidates from genetic studies to cell types present in lesional skin (HuGENet database).** Human genes were assigned to one of 10 cell types present in psoriasis lesions (see Results and Methods). Top: Analysis of 357 genes associated with psoriasis in either GWAS or non-GWAS population-based studies (HuGENet database). Bottom: Combined analysis of the 357 genes associated with psoriasis from the HuGENet database and the set of 117 GWAS-only gene candidates (439 unique genes total). Magenta labels denote those cell types for which the number of assigned genes was significantly large in comparison to known human genes (i.e., all genes represented on the Affymetrix Human Genome U133 Plus 2.0 array platform) (one asterisk, P < 0.05; two asterisks, FDR < 0.05; Fisher’s Exact Test). Click here for file

Additional file 14 **Cluster analysis of 439 genes genetically associated with psoriasis in population-based genetic studies and their expression across 10 cell types.** We identified 117 genes near candidate loci identified from GWAS studies and combined these with a set of 357 genes associated with psoriasis (GWAS or non-GWAS studies) in the HuGENet database (439 genes total). These genes were clustered based upon their expression pattern across 10 cell types (Euclidean distance and average linkage). The red-blue heatmap shows the expression of genes in each cell type, with red colors indicating relatively high expression (compared to normal human skin) and blue colors indicating relatively low expression (compared to normal human skin). The yellow-black heatmap shows the cell type assigned to each gene (i.e., the cell type for which the gene’s median expression was highest, with detection frequency greater than 10%). The chart on the far right shows the estimated median fold-change (PP/PN) for each gene (*n* = 216 patients). Click here for file

Additional file 15 ***TNFRSF9 *** **mRNA is elevated in psoriasis lesions and *TNFRSF9 *** **mRNA and protein levels are highest in neutrophils as compared to other cell types.** (A) *TNFRSF9* mRNA expression in five cell types (*n* > 4 for each cell type). (B) *TNFRSF9* mRNA is significantly elevated in psoriasis lesions as compared to uninvolved and normal skin (*n* = 11). (C) TNFRS9 (CD137) protein expression is highest in neutrophils as compared to T-cells and monocytes isolated from the peripheral blood. (D) Flow cytometric analysis shows TNFRSF9-positive neutrophils over isotype control (negative cells). In parts (A) – (C), expression is normalized to the housekeeping gene large ribosomal protein P0 (*RPLP0*). Bars represent mean ± 1SD (*P < 0.05, **P < 0.01, ***P < 0.001). Click here for file

Additional file 16 **Identification and prioritization of transcription factor binding sites sensitive to susceptibility-associated variation near psoriasis intergenic risk loci.** Procedures outlined in Figures 7 and 8 were repeated with respect to six genes near intergenic risk loci (*TNFRSF9*, *B3GNT2*, *IL12B*, *TAGAP*, *KLF4* and *NFKBIA*). Top: Expression of each gene was evaluated across 10 cell types (*n* = 50 microarray samples per cell type). Expression is normalized to that observed in normal human skin and values in yellow denote the gene’s detection frequency. The candidate cell type for each gene is shown in magenta font. Bottom: Top 10 binding sites most strongly enriched among sequences near co-expressed genes identified with respect to the candidate cell type. The listed binding sites include only those that are sensitive to variation at a SNP locus in linkage disequilibrium with the lead SNP (*r*^2^ > 0.90). The chart shows the *Z* statistic quantifying the degree to which each binding site is enriched among sequences adjacent to co-expressed genes. Associated p-values are listed in the left margin, where an asterisk symbol is used to denote FDR < 0.05. The right margin lists the SNP for which variation is predicted to influence binding. Blue font denotes binding sites for which the risk variant abrogates a match to the binding site, while red font denotes binding sites for which the risk variant engenders a match to the binding site. Click here for file

Additional file 17 **Demographic data and washout protocols for psoriasis microarray cohorts.** The table lists the number of patient samples included from each of the eight microarray studies (*n*). The table also lists known demographic data on each patient cohort and the disease severity of patients admitted into each study. The final two columns indicate reported washout protocols. This represents the period of time for which participants discontinued systemic or topical medications prior to biopsy collections. In some cases, information was not provided in original research reports and thus it was not possible to provide a complete description for all studies. Click here for file

Additional file 18 **Quality control (QC) processing of psoriasis microarray data.** The dataset was generated by pooling samples across 8 prior studies (GSE13355, GSE14905, GSE30999, GSE34248, GSE41662, GSE41663, GSE47751 and GSE50790), which yielded an initial set of 458 paired PP and PN samples from 229 patients. All samples were generated using the same commercial microarray platform (Affymetrix Human Genome U133 Plus 2.0 array). The 458 samples were evaluated with respect to (A) Average background, (B) Scale factor, (C) RNA degradation score, (D) Percentage of probe sets called present, (E) NUSE median, (F) NUSE IQR, (G) RLE median and (H) RLE IQR. Parts (A) – (H) show QC results for each of these metrics with respect to each dataset individually (top) as well as the combination across all datasets (bottom). Yellow symbols represent microarray samples excluded on the basis of QC criteria. Following these steps, there remained 434 samples from 217 patients. (I) For the remaining patients, we calculated the average fold-change among 434 genes with strongly elevated expression (median FC > 2.0 and FDR < 0.05; *n* = 217), and the average fold-change among 194 genes with strongly repressed expression (median FC < 0.50 and FDR < 0.05; *n* = 217). This revealed one outlier patient with trends opposite to those expected. The patient was thus removed from the dataset, yielding 432 samples from 216 patients. (J) The 216 remaining patients were clustered based upon PP versus PN differences observed for 16358 skin-expressed genes (Euclidean distance metric with complete linkage). Click here for file
