# Supplementary material for: Cellular dissection of psoriasis for transcriptome analyses and the post-GWAS era
Source: BMC Med Genomics. 2014 May 22;7:27. doi: 10.1186/1755-8794-7-27 (PMC4060870; doi:10.1186/1755-8794-7-27)
Supplement: Additional file 1 — Genes significantly elevated in psoriasis lesions ( n = 216 patients). The table lists the 1019 differentially expressed genes (DEGs) significantly elevated in psoriasis lesions (FC > 1.50, FDR < 0.05). DEGs are sorted according to their median fold-change in lesional skin as compared to uninvolved skin (n = 216 patients). The table lists the frequency with which each gene was detected significantly above background among PP samples (third column) and PN samples (fourth column) (Wilcoxon signed rank test, P < 0.05). P-values for differential expression were calculated using the Wilcoxon rank sum test (fifth column) and FDR-adjusted p-values were calculated using the Benjamini-Hochberg method (final column). [file 1755-8794-7-27-S1.pdf]

Additional File 1. Genes significantly elevated in psoriasis lesions (*n* = 216 patients). The table lists the 1019 differentially expressed genes (DEGs) significantly elevated in psoriasis lesions (FC > 1.50, FDR < 0.05). DEGs are sorted according to their median fold-change in lesional skin as compared to uninvolved skin (*n* = 216 patients). The table lists the frequency with which each gene was detected significantly above background among PP samples (third column) and PN samples (fourth column) (Wilcoxon signed rank test, P < 0.05). P-values for differential expression were calculated using the Wilcoxon rank sum test (fifth column) and FDR-adjusted p-values were calculated using the Benjamini-Hochberg method (final column).

Symbol (Probe ID)	FC (PP/PN)	Freq (PP)	Freq (PN)	P-value	FDR
SERPINB4 (211906_s_at)	217.6	1.00	0.77	3.75E-37	4.29E-35
S100A12 (205863_at)	61.95	1.00	0.33	3.5E-37	4.29E-35
TCN1 (205513_at)	57.11	1.00	0.80	3.75E-37	4.29E-35
S100A7A (232170_at)	54.88	1.00	0.61	3.55E-37	4.29E-35
SPRR2C (220664_at)	49.52	1.00	0.58	3.5E-37	4.29E-35
DEFB4A (207356_at)	30.13	1.00	0.68	3.8E-37	4.29E-35
S100A9 (203535_at)	26.61	1.00	0.98	3.7E-37	4.29E-35
SERPINB3 (209720_s_at)	25.53	1.00	0.99	3.6E-37	4.29E-35
AKR1B10 (206561_s_at)	24.31	1.00	0.92	3.55E-37	4.29E-35
PI3 (203691_at)	19.33	1.00	0.84	3.5E-37	4.29E-35
IL36G (220322_at)	16.73	1.00	0.98	3.45E-37	4.29E-35
KYNU (217388_s_at)	16.55	1.00	0.91	3.45E-37	4.29E-35
LCN2 (212531_at)	16.22	1.00	0.82	3.65E-37	4.29E-35
TMPRSS11D (207602_at)	14.96	0.95	0.02	3.55E-37	4.29E-35
RHCG (219554_at)	14.56	0.99	0.55	3.45E-37	4.29E-35
ATP12A (207367_at)	13.96	1.00	0.71	3.45E-37	4.29E-35
OASL (205660_at)	13.41	1.00	0.72	3.5E-37	4.29E-35
VNN3 (220528_at)	13.15	0.92	0.05	3.5E-37	4.29E-35
HPSE (219403_s_at)	12.90	1.00	0.70	3.45E-37	4.29E-35
IGFL1 (239430_at)	12.80	0.99	0.16	4.37E-37	4.29E-35
ADAMDEC1 (206134_at)	12.21	1.00	0.83	6.19E-37	4.29E-35
C10orf99 (227736_at)	11.50	1.00	0.90	3.5E-37	4.29E-35
KLK6 (204733_at)	11.30	0.99	0.72	4.25E-37	4.29E-35
GDA (224209_s_at)	10.91	1.00	0.85	3.55E-37	4.29E-35
CXCL13 (205242_at)	10.34	0.97	0.36	1.1E-36	5.01E-35
KLK13 (205783_at)	9.99	1.00	0.99	3.65E-37	4.29E-35
CCL20 (205476_at)	9.82	0.83	0.20	3.45E-37	4.29E-35
LTF (202018_s_at)	9.56	0.99	0.67	1.5E-36	5.71E-35
KRT16 (209800_at)	9.31	1.00	1.00	3.8E-37	4.29E-35
CXCL1 (204470_at)	9.30	0.98	0.33	3.91E-37	4.29E-35
PRSS27 (232074_at)	8.65	0.95	0.36	3.45E-37	4.29E-35
CHI3L2 (213060_s_at)	8.63	1.00	0.84	4.25E-37	4.29E-35
MMP12 (204580_at)	8.28	0.99	0.73	9.12E-35	1.29E-33
OAS2 (204972_at)	7.84	1.00	0.93	4.02E-37	4.29E-35
GZMB (210164_at)	7.54	0.97	0.64	4.14E-37	4.29E-35
LCE3D (224328_s_at)	7.51	1.00	1.00	3.7E-37	4.29E-35
ZC3H12A (218810_at)	6.93	1.00	0.80	3.8E-37	4.29E-35
HERC6 (219352_at)	6.88	1.00	0.98	3.8E-37	4.29E-35
TNIP3 (220655_at)	6.63	0.77	0.02	4.82E-37	4.29E-35

CD274 (227458_at)	6.62	1.00	0.93	3.5E-37	4.29E-35
CXCL10 (204533_at)	6.18	1.00	0.84	4.46E-35	7.12E-34
CHRNA9 (221107_at)	5.96	0.99	0.66	5.03E-37	4.29E-35
IL19 (220745_at)	5.96	0.87	0.07	1.14E-36	5.08E-35
HYAL4 (220249_at)	5.90	0.96	0.44	4.02E-37	4.29E-35
ARSF (214490_at)	5.90	0.94	0.16	4.56E-37	4.29E-35
RSAD2 (213797_at)	5.68	0.91	0.57	4.65E-36	1.2E-34
SAMD9 (228531_at)	5.39	1.00	1.00	4.19E-37	4.29E-35
FOXE1 (206912_at)	5.28	0.98	0.67	3.86E-37	4.29E-35
TGM1 (206008_at)	5.26	1.00	0.94	3.7E-37	4.29E-35
PLA2G4D (1554914_at)	5.22	0.96	0.51	3.75E-37	4.29E-35
MX1 (202086_at)	5.08	1.00	1.00	3.65E-37	4.29E-35
IFI6 (204415_at)	4.95	0.98	0.88	7.42E-37	4.43E-35
SLC26A9 (242271_at)	4.94	1.00	0.87	4.14E-37	4.29E-35
MMP1 (204475_at)	4.92	0.90	0.28	1.48E-34	1.99E-33
FCHSD1 (226698_at)	4.91	0.95	0.65	3.6E-37	4.29E-35
SLC6A14 (219795_at)	4.90	1.00	1.00	5.1E-37	4.29E-35
VNN1 (205844_at)	4.89	0.98	0.74	5.7E-37	4.29E-35
UPP1 (203234_at)	4.82	0.98	0.49	3.8E-37	4.29E-35
CHAC1 (219270_at)	4.79	0.95	0.26	9.13E-37	4.76E-35
IL36A (221404_at)	4.75	0.85	0.03	3.7E-37	4.29E-35
KLHDC7B (236285_at)	4.75	1.00	0.79	6.11E-37	4.29E-35
CMPK2 (226702_at)	4.63	1.00	0.99	1.06E-36	4.97E-35
XDH (241994_at)	4.62	0.99	0.90	7.02E-37	4.4E-35
ISG15 (205483_s_at)	4.57	1.00	0.97	6.19E-37	4.29E-35
IL36RN (222223_s_at)	4.54	1.00	1.00	3.6E-37	4.29E-35
PRKCQ (210038_at)	4.42	0.93	0.56	4.5E-37	4.29E-35
GBP6 (1559607_s_at)	4.32	0.97	0.48	4.62E-37	4.29E-35
CNFN (224329_s_at)	4.31	1.00	1.00	3.6E-37	4.29E-35
CXCL2 (209774_x_at)	4.28	0.93	0.49	1.29E-36	5.35E-35
OAS3 (218400_at)	4.27	1.00	1.00	3.86E-37	4.29E-35
KCNJ15 (210119_at)	4.25	1.00	0.96	3.5E-37	4.29E-35
TMC5 (222904_s_at)	4.23	1.00	0.89	3.97E-37	4.29E-35
IFI27 (202411_at)	4.21	1.00	1.00	3.86E-37	4.29E-35
PGBD5 (219225_at)	4.17	0.94	0.49	7.42E-37	4.43E-35
LRG1 (228648_at)	4.11	0.99	0.82	1.9E-36	6.5E-35
CXCL9 (203915_at)	4.09	1.00	0.89	3.26E-34	3.99E-33
LOC100505839 (1557389_at)	4.02	0.99	0.71	4.25E-37	4.29E-35
TGM3 (206004_at)	4.00	1.00	0.98	7.95E-37	4.55E-35
SLC5A1 (206628_at)	4.00	1.00	0.96	3.8E-37	4.29E-35
IFI44L (204439_at)	3.95	0.99	0.96	1.88E-34	2.46E-33
SLC7A11 (209921_at)	3.94	1.00	0.93	4.5E-37	4.29E-35
S100A8 (202917_s_at)	3.89	1.00	1.00	3.6E-37	4.29E-35
FUT3 (214088_s_at)	3.89	0.99	0.70	1.46E-36	5.66E-35
CXCR2 (207008_at)	3.88	1.00	0.95	4.14E-37	4.29E-35
PDZK1IP1 (219630_at)	3.86	1.00	1.00	4.62E-37	4.29E-35
CCNB1 (214710_s_at)	3.86	1.00	1.00	5.46E-37	4.29E-35
CYP4Z2P (1553434_at)	3.83	0.80	0.22	3.49E-35	5.85E-34
RNASE7 (234700_s_at)	3.81	1.00	0.98	6.26E-34	7.16E-33

TTC39A (210652_s_at)	3.80	1.00	0.97	3.75E-37	4.29E-35
SOST (223869_at)	3.80	0.83	0.36	1.02E-33	1.12E-32
SPRR3 (218990_s_at)	3.78	0.67	0.04	9.39E-37	4.79E-35
KLK10 (209792_s_at)	3.76	1.00	1.00	3.75E-37	4.29E-35
IRF7 (208436_s_at)	3.72	1.00	0.95	5.46E-37	4.29E-35
PLAT (201860_s_at)	3.71	1.00	1.00	1.11E-33	1.2E-32
OAS1 (202869_at)	3.68	1.00	0.88	4.82E-37	4.29E-35
GJB2 (223278_at)	3.68	1.00	1.00	3.7E-37	4.29E-35
A2ML1 (1564307_a_at)	3.67	1.00	1.00	3.55E-37	4.29E-35
TMPRSS4 (218960_at)	3.66	0.98	0.22	4.37E-37	4.29E-35
CCL18 (32128_at)	3.58	1.00	0.90	2.04E-29	1.19E-28
INA (204465_s_at)	3.57	0.93	0.60	3.5E-37	4.29E-35
IFIT1 (203153_at)	3.56	1.00	0.99	4.71E-35	7.45E-34
IFIT3 (229450_at)	3.51	1.00	1.00	1.38E-36	5.52E-35
REN (206367_at)	3.44	0.58	0.07	1.11E-36	5.02E-35
KRT6A (209125_at)	3.40	1.00	1.00	3.86E-37	4.29E-35
DLGAP5 (203764_at)	3.40	1.00	0.97	4.62E-37	4.29E-35
RTP4 (219684_at)	3.38	0.95	0.51	5.7E-37	4.29E-35
GDPD3 (219722_s_at)	3.36	1.00	1.00	3.75E-37	4.29E-35
SAMSN1 (220330_s_at)	3.36	1.00	0.98	1.26E-36	5.3E-35
FAM83A (239586_at)	3.35	0.99	0.59	3.75E-37	4.29E-35
TMEM45B (230323_s_at)	3.34	1.00	1.00	3.6E-37	4.29E-35
IL8 (211506_s_at)	3.32	0.70	0.06	6.11E-36	1.47E-34
SPRR1A (213796_at)	3.31	1.00	1.00	4.43E-37	4.29E-35
SDR9C7 (1553077_at)	3.25	0.98	0.72	2.83E-36	8.6E-35
LIPG (219181_at)	3.24	0.91	0.63	2.26E-35	4.11E-34
KIAA0101 (202503_s_at)	3.21	1.00	1.00	3.97E-37	4.29E-35
ACPP (204393_s_at)	3.21	1.00	1.00	1.02E-36	4.9E-35
SERPINA1 (211429_s_at)	3.20	1.00	0.92	8.17E-37	4.58E-35
FOSL1 (204420_at)	3.20	0.89	0.54	3.3E-36	9.42E-35
CYP24A1 (206504_at)	3.20	0.87	0.63	9.73E-36	2.08E-34
FUT2 (210608_s_at)	3.20	1.00	0.99	3.45E-37	4.29E-35
FAM43A (227410_at)	3.20	1.00	1.00	4.08E-37	4.29E-35
ADAP2 (219358_s_at)	3.20	1.00	1.00	4.02E-37	4.29E-35
ABCA12 (215465_at)	3.19	1.00	1.00	3.75E-37	4.29E-35
RGS1 (202988_s_at)	3.18	0.95	0.71	3.97E-37	4.29E-35
NETO2 (218888_s_at)	3.18	1.00	0.95	7.21E-37	4.43E-35
IFI44 (214453_s_at)	3.17	1.00	0.98	3.21E-36	9.29E-35
SERPINB13 (217272_s_at)	3.17	1.00	1.00	3.45E-37	4.29E-35
ALOX12B (207381_at)	3.17	1.00	0.99	6.37E-37	4.29E-35
SLAMF7 (222838_at)	3.16	1.00	0.76	2.34E-36	7.59E-35
PNP (201695_s_at)	3.15	1.00	1.00	3.86E-37	4.29E-35
IL7R (226218_at)	3.13	1.00	0.88	7.2E-36	1.65E-34
WNT5A (213425_at)	3.12	1.00	0.97	3.45E-37	4.29E-35
RRM2 (201890_at)	3.11	1.00	1.00	9.79E-37	4.83E-35
HS3ST3A1 (219985_at)	3.10	0.96	0.72	5.7E-37	4.29E-35
FLVCR2 (222866_s_at)	3.08	1.00	0.93	3.65E-37	4.29E-35
IL17A (216876_s_at)	3.06	0.83	0.07	9.79E-37	4.83E-35
LAMP3 (205569_at)	3.06	1.00	1.00	3.5E-37	4.29E-35

DHRS9 (219799_s_at)	3.05	1.00	0.96	2.7E-34	3.38E-33
LOC439949 (232001_at)	3.05	0.97	0.71	3.8E-37	4.29E-35
TMEM86A (227570_at)	3.04	1.00	0.91	9.13E-37	4.76E-35
PCP4L1 (241382_at)	3.03	1.00	0.91	3.09E-35	5.26E-34
IDO1 (210029_at)	3.02	0.74	0.07	4.82E-37	4.29E-35
ATP10B (214070_s_at)	3.02	1.00	1.00	4.5E-37	4.29E-35
SCO2 (205241_at)	3.02	1.00	1.00	5.62E-37	4.29E-35
APOBEC3A (210873_x_at)	3.01	0.94	0.56	9.36E-35	1.32E-33
BCL2A1 (205681_at)	3.01	1.00	0.84	5.24E-37	4.29E-35
CDH26 (232306_at)	3.00	0.85	0.37	1.16E-36	5.11E-35
AREG (205239_at)	3.00	1.00	0.96	7.95E-37	4.55E-35
ESYT3 (1554912_at)	2.99	1.00	1.00	6.37E-36	1.51E-34
CDK5R1 (204995_at)	2.98	1.00	0.98	3.75E-37	4.29E-35
TYMP (217497_at)	2.98	1.00	0.93	1.01E-36	4.88E-35
KIF20A (218755_at)	2.97	0.94	0.64	8.52E-37	4.67E-35
CLEC7A (221698_s_at)	2.97	1.00	1.00	3.5E-37	4.29E-35
IL26 (221111_at)	2.96	0.73	0.16	4.78E-36	1.21E-34
KIAA1239 (230765_at)	2.95	0.75	0.37	1.68E-36	6.07E-35
AIM2 (206513_at)	2.94	0.99	0.81	3.91E-37	4.29E-35
CCNE1 (213523_at)	2.92	1.00	0.76	4.19E-37	4.29E-35
C12orf5 (219099_at)	2.92	1.00	1.00	4.02E-37	4.29E-35
GBP1 (202270_at)	2.92	1.00	1.00	7.02E-37	4.4E-35
CRABP2 (202575_at)	2.92	1.00	1.00	3.65E-37	4.29E-35
C12orf56 (236840_at)	2.89	0.73	0.51	4.31E-37	4.29E-35
TEX101 (223906_s_at)	2.89	0.64	0.30	4.96E-37	4.29E-35
SPRR1B (205064_at)	2.89	1.00	1.00	3.65E-37	4.29E-35
TTK (204822_at)	2.88	1.00	1.00	1.26E-36	5.3E-35
TDO2 (205943_at)	2.87	0.94	0.55	5.61E-35	8.57E-34
CARD6 (224414_s_at)	2.87	0.99	0.83	5.94E-37	4.29E-35
PARP9 (223220_s_at)	2.87	1.00	0.99	3.55E-37	4.29E-35
PLBD1 (218454_at)	2.86	1.00	1.00	3.5E-37	4.29E-35
C9orf169 (1569144_a_at)	2.86	1.00	1.00	2.09E-36	6.98E-35
ZBED2 (219836_at)	2.84	1.00	0.80	4.56E-37	4.29E-35
XAF1 (228617_at)	2.82	1.00	1.00	5.55E-36	1.36E-34
GK3P (215966_x_at)	2.81	0.94	0.35	5.7E-37	4.29E-35
GPR68 (229055_at)	2.80	1.00	0.98	3.75E-37	4.29E-35
WFDC12 (1553081_at)	2.80	1.00	0.95	1.91E-31	1.41E-30
SLC16A10 (222939_s_at)	2.80	1.00	0.98	6.02E-37	4.29E-35
CFB (202357_s_at)	2.80	0.99	0.85	6.37E-37	4.29E-35
C9orf84 (233504_at)	2.80	0.83	0.57	1.14E-36	5.08E-35
CTLA4 (236341_at)	2.78	0.87	0.56	8.97E-36	1.95E-34
PGLYRP4 (220944_at)	2.78	1.00	0.97	3.86E-37	4.29E-35
NOD2 (220066_at)	2.77	1.00	1.00	3.55E-37	4.29E-35
PBK (219148_at)	2.76	1.00	0.99	2.76E-36	8.4E-35
ARG1 (206177_s_at)	2.76	1.00	1.00	1.01E-36	4.88E-35
KRT17 (205157_s_at)	2.76	1.00	1.00	3.74E-35	6.19E-34
WDR66 (230193_at)	2.75	0.94	0.76	2.4E-36	7.7E-35
IL1B (39402_at)	2.75	0.92	0.35	1.01E-36	4.88E-35
CKS2 (204170_s_at)	2.75	1.00	1.00	5.31E-37	4.29E-35

MELK (204825_at)	2.74	1.00	0.96	4.43E-37	4.29E-35
GALNT6 (228303_at)	2.73	0.95	0.56	6.64E-37	4.31E-35
AURKA (208079_s_at)	2.73	0.99	0.91	7.52E-37	4.46E-35
SFT2D2 (214838_at)	2.73	1.00	0.93	4.43E-37	4.29E-35
IFIH1 (219209_at)	2.72	1.00	1.00	5.46E-37	4.29E-35
POLR3G (206653_at)	2.72	1.00	0.98	4.82E-37	4.29E-35
PPIF (201489_at)	2.71	1.00	1.00	3.5E-37	4.29E-35
CYP7B1 (207386_at)	2.71	1.00	0.93	3.65E-37	4.29E-35
CYP2C18 (208126_s_at)	2.70	0.99	0.92	3.18E-33	3.1E-32
IL20 (224071_at)	2.70	0.72	0.01	5.94E-37	4.29E-35
EPHB2 (209588_at)	2.70	0.78	0.38	1.63E-36	5.95E-35
HSD17B2 (204818_at)	2.69	0.98	0.73	2.15E-31	1.57E-30
GGH (203560_at)	2.67	1.00	1.00	4.31E-37	4.29E-35
SLC7A5 (201195_s_at)	2.66	1.00	0.93	9.52E-37	4.79E-35
IRAK2 (231779_at)	2.66	1.00	0.98	3.91E-37	4.29E-35
NT5C3 (223298_s_at)	2.65	1.00	1.00	3.97E-37	4.29E-35
VMP1 (1569003_at)	2.65	0.99	0.78	4.37E-37	4.29E-35
GPX2 (202831_at)	2.65	1.00	0.89	1.54E-36	5.78E-35
ZDHHC21 (235068_at)	2.65	1.00	1.00	6.19E-37	4.29E-35
LRRC55 (232856_at)	2.65	0.60	0.24	3.78E-36	1.03E-34
APOL1 (209546_s_at)	2.64	0.96	0.71	1.09E-36	4.99E-35
ALDH1A3 (203180_at)	2.63	1.00	0.98	3.82E-34	4.6E-33
IL12B (207901_at)	2.62	0.83	0.26	4.05E-36	1.09E-34
FAM110C (226863_at)	2.62	1.00	1.00	4.14E-37	4.29E-35
CCNB2 (202705_at)	2.62	1.00	1.00	8.4E-37	4.66E-35
CHAC2 (235117_at)	2.62	1.00	0.92	4.37E-37	4.29E-35
PRSS53 (214226_at)	2.61	0.88	0.51	6.19E-37	4.29E-35
HBEGF (38037_at)	2.60	0.96	0.68	9.01E-37	4.75E-35
DEPDC1B (226980_at)	2.60	1.00	0.87	1.33E-36	5.4E-35
PRSS3 (213421_x_at)	2.60	1.00	1.00	5.24E-37	4.29E-35
LOC388820 (243955_at)	2.60	0.64	0.57	4.4E-36	1.15E-34
KIAA1199 (212942_s_at)	2.60	1.00	0.93	5.39E-37	4.29E-35
IVL (214599_at)	2.59	1.00	1.00	5.39E-37	4.29E-35
FGFBP1 (205014_at)	2.59	1.00	1.00	4.22E-35	6.84E-34
PLA2G2F (221416_at)	2.59	0.88	0.31	1.02E-36	4.9E-35
GRHL3 (232116_at)	2.58	1.00	1.00	4.56E-37	4.29E-35
PI15 (229947_at)	2.58	1.00	0.96	4.31E-34	5.09E-33
PLA2G3 (220780_at)	2.58	0.84	0.54	6.46E-37	4.29E-35
MKI67 (212022_s_at)	2.57	0.98	0.73	3.08E-36	9.08E-35
OTUB2 (219369_s_at)	2.57	1.00	1.00	1.92E-35	3.62E-34
EPSTI1 (235276_at)	2.56	1.00	0.97	1.42E-36	5.56E-35
FAM83D (225687_at)	2.56	1.00	1.00	2.46E-35	4.38E-34
CH25H (206932_at)	2.56	1.00	0.96	4.71E-36	1.21E-34
FBXO45 (225099_at)	2.56	1.00	1.00	3.48E-36	9.78E-35
HAL (206643_at)	2.56	1.00	1.00	7.42E-37	4.43E-35
C15orf48 (223484_at)	2.55	1.00	1.00	3.31E-35	5.57E-34
ASPM (219918_s_at)	2.54	1.00	0.99	8.37E-36	1.86E-34
CERS3 (1554253_a_at)	2.53	1.00	1.00	4.62E-37	4.29E-35
SERPINA3 (202376_at)	2.53	0.98	0.93	1.79E-31	1.33E-30

CEP55 (218542_at)	2.53	1.00	0.94	1.22E-36	5.24E-35
SPRR2G (236119_s_at)	2.52	1.00	1.00	1E-35	2.12E-34
CDKN3 (209714_s_at)	2.52	1.00	1.00	1.14E-36	5.08E-35
ST6GALNAC1 (227725_at)	2.52	0.94	0.69	9.01E-37	4.75E-35
SOCS3 (227697_at)	2.50	1.00	0.97	2.42E-35	4.34E-34
CCR7 (206337_at)	2.49	0.95	0.43	3.21E-36	9.29E-35
NAMPT (217739_s_at)	2.49	1.00	1.00	3.7E-37	4.29E-35
SPC25 (209891_at)	2.48	0.99	0.80	2.54E-36	7.99E-35
FAM65C (227654_at)	2.48	1.00	0.97	6.55E-37	4.29E-35
SMOX (210357_s_at)	2.48	1.00	0.86	6.11E-37	4.29E-35
NDC80 (204162_at)	2.48	0.99	0.93	1.19E-36	5.2E-35
TMEM171 (240770_at)	2.47	0.93	0.56	9.01E-37	4.75E-35
LOC100505702 (229635_at)	2.47	1.00	0.95	6.91E-36	1.6E-34
KLRB1 (214470_at)	2.47	0.86	0.63	3.21E-36	9.29E-35
DNASE1L3 (205554_s_at)	2.47	1.00	1.00	1.48E-36	5.69E-35
APOBEC3B (206632_s_at)	2.47	1.00	1.00	3.48E-36	9.78E-35
GZMA (205488_at)	2.46	0.98	0.82	2.85E-35	4.94E-34
FAM26F (229391_s_at)	2.46	1.00	0.79	5.71E-36	1.39E-34
ARNTL2 (223586_at)	2.46	1.00	0.84	4.08E-37	4.29E-35
PRR11 (228273_at)	2.46	1.00	1.00	6.92E-37	4.38E-35
FABP5 (202345_s_at)	2.45	1.00	1.00	3.55E-37	4.29E-35
CDC20 (202870_s_at)	2.45	1.00	0.97	6.91E-36	1.6E-34
SMPD3 (219695_at)	2.45	0.99	0.88	1.46E-36	5.66E-35
DSC2 (226817_at)	2.45	1.00	1.00	3.97E-37	4.29E-35
MPZL2 (203780_at)	2.45	1.00	1.00	3.5E-37	4.29E-35
HTR3A (216615_s_at)	2.44	0.82	0.21	3.3E-34	4.03E-33
HMOX1 (203665_at)	2.44	1.00	0.99	1.06E-35	2.22E-34
TRIM10 (221627_at)	2.44	0.73	0.37	1.06E-36	4.97E-35
GNA15 (205349_at)	2.43	1.00	0.99	4.08E-37	4.29E-35
FBXO6 (231769_at)	2.43	1.00	1.00	5.54E-37	4.29E-35
DDX60L (228152_s_at)	2.43	0.97	0.91	4E-35	6.53E-34
DCUN1D3 (239648_at)	2.41	1.00	1.00	4.31E-37	4.29E-35
STEAP1B (217553_at)	2.41	0.84	0.61	1.23E-35	2.52E-34
LYPD5 (236039_at)	2.41	0.99	0.84	5.19E-36	1.29E-34
SLC16A6 (230748_at)	2.41	1.00	1.00	4.25E-37	4.29E-35
ISG20 (33304_at)	2.41	0.92	0.37	5.1E-37	4.29E-35
LDLR (202068_s_at)	2.40	1.00	1.00	2.91E-36	8.74E-35
GJB6 (231771_at)	2.39	1.00	1.00	3.55E-37	4.29E-35
PRDM1 (228964_at)	2.39	1.00	1.00	2.91E-36	8.74E-35
UBE2F (225783_at)	2.39	1.00	1.00	3.55E-37	4.29E-35
PHLDA2 (209803_s_at)	2.39	1.00	0.94	2.87E-36	8.65E-35
S100P (204351_at)	2.38	1.00	0.99	1.11E-27	5.49E-27
ADAM23 (244463_at)	2.38	0.82	0.63	1.14E-36	5.08E-35
FCGR3B (204007_at)	2.38	0.99	0.84	1.06E-33	1.16E-32
TC2N (1553132_a_at)	2.38	1.00	1.00	4.02E-37	4.29E-35
HMMR (207165_at)	2.38	1.00	0.97	2.34E-36	7.59E-35
TPX2 (210052_s_at)	2.37	1.00	0.89	1.05E-36	4.96E-35
LRP8 (228955_at)	2.37	0.98	0.87	8.64E-37	4.73E-35
CDC6 (203967_at)	2.37	0.98	0.84	8.49E-36	1.87E-34

EPHX3 (220013_at)	2.36	1.00	1.00	5.39E-37	4.29E-35
LCK (204891_s_at)	2.36	0.99	0.87	3.79E-35	6.26E-34
SELE (206211_at)	2.35	0.99	0.95	4.91E-33	4.61E-32
MX2 (204994_at)	2.34	1.00	1.00	7.15E-34	8.07E-33
TOP2A (201292_at)	2.34	1.00	1.00	2.76E-36	8.4E-35
GBAP1 (210589_s_at)	2.34	1.00	0.97	5.24E-37	4.29E-35
BUB1 (209642_at)	2.33	0.98	0.74	6.55E-36	1.54E-34
UBE2L6 (201649_at)	2.33	1.00	1.00	4.69E-37	4.29E-35
PTTG1 (203554_x_at)	2.33	1.00	1.00	5.1E-37	4.29E-35
IL4R (203233_at)	2.33	1.00	0.85	4.08E-37	4.29E-35
CDK1 (203213_at)	2.32	1.00	1.00	9.39E-37	4.79E-35
DDX60 (218986_s_at)	2.32	1.00	1.00	4.46E-35	7.12E-34
IL36B (231755_at)	2.32	0.77	0.52	8.76E-35	1.25E-33
SELL (204563_at)	2.32	0.97	0.88	1.86E-34	2.43E-33
NLRP2 (221690_s_at)	2.31	0.72	0.52	2.44E-28	1.28E-27
DSG3 (235075_at)	2.31	1.00	1.00	3.75E-37	4.29E-35
TREX2 (211788_s_at)	2.30	0.43	0.01	6.37E-37	4.29E-35
GK (214681_at)	2.30	0.99	0.88	2.68E-36	8.28E-35
MUC4 (217109_at)	2.30	0.73	0.35	6.96E-35	1.03E-33
ELF3 (210827_s_at)	2.30	0.99	0.90	2.5E-36	7.93E-35
CDH3 (203256_at)	2.29	1.00	0.97	1.48E-36	5.69E-35
SPCS3 (218817_at)	2.29	1.00	1.00	1.17E-36	5.15E-35
LAIR2 (207509_s_at)	2.29	0.82	0.24	6.78E-35	1E-33
UNC93A (214382_at)	2.29	0.99	0.97	1.44E-33	1.52E-32
CD2 (205831_at)	2.29	1.00	0.97	1.58E-34	2.11E-33
MICALL1 (55081_at)	2.28	1.00	1.00	6.55E-37	4.29E-35
NEK2 (204641_at)	2.28	1.00	0.96	3.18E-35	5.37E-34
F12 (205774_at)	2.28	1.00	0.90	4.25E-37	4.29E-35
TMEM40 (222892_s_at)	2.27	1.00	1.00	5.39E-37	4.29E-35
KIF4A (218355_at)	2.27	1.00	0.96	1.75E-36	6.2E-35
FCGR1B (214511_x_at)	2.25	0.82	0.34	1.57E-30	1.03E-29
CASP5 (207500_at)	2.25	0.82	0.18	3.68E-36	1.01E-34
CCL22 (207861_at)	2.25	0.71	0.57	7.54E-34	8.46E-33
KPNA2 (211762_s_at)	2.24	1.00	1.00	4.82E-37	4.29E-35
KLK8 (206125_s_at)	2.24	1.00	1.00	3.3E-36	9.42E-35
KIF2C (209408_at)	2.23	1.00	0.97	8.06E-37	4.57E-35
USP18 (219211_at)	2.23	0.92	0.78	3.82E-33	3.66E-32
TNFRSF21 (218856_at)	2.22	1.00	0.98	5.78E-37	4.29E-35
CCRN4L (1554283_at)	2.22	0.97	0.78	6.82E-37	4.38E-35
RAET1E (1552777_a_at)	2.22	0.97	0.92	9.75E-35	1.37E-33
EPHA2 (203499_at)	2.22	1.00	1.00	9.79E-37	4.83E-35
ATP13A4 (1557136_at)	2.21	1.00	0.99	8.18E-35	1.18E-33
VSNL1 (203797_at)	2.21	1.00	1.00	4.14E-37	4.29E-35
CYP4F22 (244692_at)	2.21	1.00	0.99	1.95E-35	3.65E-34
PRSS2 (205402_x_at)	2.21	0.98	0.78	5.54E-37	4.29E-35
CRCT1 (220620_at)	2.21	1.00	0.99	1.57E-32	1.35E-31
C12orf75 (225105_at)	2.20	1.00	1.00	1.73E-36	6.15E-35
MMP9 (203936_s_at)	2.20	1.00	0.99	3.27E-28	1.69E-27
AASS (210852_s_at)	2.20	1.00	0.90	4.62E-37	4.29E-35

CST7 (210140_at)	2.20	0.98	0.70	1.24E-35	2.55E-34
LOC100506411 (1565836_at)	2.20	0.68	0.62	9.24E-35	1.31E-33
AUNIP (220011_at)	2.20	0.97	0.87	1.7E-36	6.12E-35
CENPE (205046_at)	2.20	0.97	0.81	8.4E-37	4.66E-35
MIR155HG (229437_at)	2.20	0.93	0.77	1.4E-34	1.9E-33
C17orf96 (228066_at)	2.19	0.69	0.61	2.3E-36	7.54E-35
SGPP2 (238567_at)	2.19	1.00	0.99	7.74E-34	8.67E-33
GPT2 (224839_s_at)	2.19	1.00	1.00	8.61E-36	1.9E-34
S100A7 (205916_at)	2.19	1.00	1.00	4.08E-37	4.29E-35
UBE2C (202954_at)	2.19	1.00	1.00	3.54E-35	5.91E-34
KIF18B (222039_at)	2.19	1.00	0.97	6.28E-37	4.29E-35
GM2A (212737_at)	2.19	1.00	1.00	3.6E-37	4.29E-35
FZD5 (221245_s_at)	2.18	1.00	1.00	9.01E-37	4.75E-35
PRIC285 (228230_at)	2.18	0.86	0.44	3.65E-37	4.29E-35
EIF4EBP1 (221539_at)	2.17	1.00	1.00	8.06E-37	4.57E-35
CCL2 (216598_s_at)	2.17	1.00	1.00	2.89E-35	4.99E-34
NABP1 (233085_s_at)	2.17	1.00	1.00	3.3E-36	9.42E-35
GJA3 (239572_at)	2.17	0.98	0.85	2.97E-35	5.1E-34
BUB1B (203755_at)	2.17	1.00	0.94	3.12E-36	9.13E-35
LYN (202625_at)	2.17	1.00	1.00	3.86E-37	4.29E-35
ID1 (208937_s_at)	2.17	1.00	1.00	4.09E-34	4.87E-33
MPHOSPH6 (203740_at)	2.17	1.00	1.00	3.6E-37	4.29E-35
LOC100996579 (236598_at)	2.16	0.98	0.79	3.45E-37	4.29E-35
MAD2L1 (203362_s_at)	2.16	1.00	1.00	2.52E-35	4.46E-34
RABIF (204478_s_at)	2.15	1.00	1.00	4.14E-37	4.29E-35
PLAC8 (219014_at)	2.15	0.85	0.49	6.46E-33	5.94E-32
HK2 (202934_at)	2.15	1.00	1.00	5.62E-37	4.29E-35
AFAP1L2 (226829_at)	2.14	1.00	1.00	4.75E-37	4.29E-35
UBE2T (223229_at)	2.14	1.00	0.97	2.65E-36	8.21E-35
EPN3 (223895_s_at)	2.14	1.00	0.98	3.1E-33	3.03E-32
RDH16 (206753_at)	2.14	0.61	0.29	3.5E-37	4.29E-35
THAP2 (230380_at)	2.14	0.98	0.87	8.06E-37	4.57E-35
DPH3 (225195_at)	2.14	1.00	1.00	6.19E-37	4.29E-35
PGM2 (225367_at)	2.14	1.00	1.00	3.97E-37	4.29E-35
C20orf24 (224376_s_at)	2.13	1.00	1.00	3.45E-37	4.29E-35
KIF23 (204709_s_at)	2.13	1.00	0.86	3.34E-36	9.53E-35
TPBG (203476_at)	2.12	1.00	1.00	3.65E-37	4.29E-35
CDCA5 (224753_at)	2.12	0.97	0.70	1.2E-36	5.21E-35
CYP27B1 (205676_at)	2.12	0.88	0.69	1.1E-35	2.3E-34
PLCXD1 (218951_s_at)	2.12	1.00	1.00	1.27E-36	5.3E-35
TRBC1 (213193_x_at)	2.12	1.00	0.99	1.09E-33	1.18E-32
EREG (205767_at)	2.12	1.00	1.00	6.27E-32	4.9E-31
TK1 (1554408_a_at)	2.11	0.99	0.78	5.92E-35	8.97E-34
SOGA2 (213358_at)	2.11	1.00	1.00	1.41E-35	2.81E-34
CXCR4 (217028_at)	2.11	1.00	0.99	5.79E-36	1.4E-34
CDCA2 (226661_at)	2.11	0.99	0.90	1.01E-36	4.88E-35
SRXN1 (225252_at)	2.10	1.00	1.00	2.34E-36	7.59E-35
STARD4 (226390_at)	2.10	0.98	0.92	1.26E-34	1.72E-33
CENPN (228559_at)	2.10	1.00	1.00	3.04E-36	9E-35

PLSCR1 (202446_s_at)	2.10	1.00	1.00	2.06E-36	6.93E-35
NLRX1 (219680_at)	2.09	1.00	0.99	5.46E-37	4.29E-35
GINS3 (45633_at)	2.09	1.00	1.00	3.6E-37	4.29E-35
RND1 (210056_at)	2.09	0.20	0.00	6.2E-36	1.48E-34
N4BP1 (32069_at)	2.09	1.00	1.00	5.17E-37	4.29E-35
CCL8 (214038_at)	2.09	0.94	0.80	3.31E-31	2.37E-30
DIO2 (231240_at)	2.09	1.00	1.00	4.6E-33	4.34E-32
CD3D (213539_at)	2.09	0.99	0.91	2E-35	3.75E-34
FAM108C1 (225436_at)	2.09	1.00	1.00	8.76E-37	4.74E-35
SERPINB9 (209723_at)	2.09	0.99	0.97	1.7E-36	6.12E-35
BIRC5 (202094_at)	2.09	0.80	0.58	1.4E-36	5.54E-35
CTPS1 (202613_at)	2.08	1.00	1.00	4.5E-37	4.29E-35
ENTPD7 (220153_at)	2.08	0.85	0.54	2.12E-35	3.89E-34
PDSS1 (220865_s_at)	2.08	1.00	1.00	1.77E-36	6.25E-35
LOC100506013 (1559280_a_at)	2.08	0.91	0.77	2.81E-32	2.33E-31
STAT1 (200887_s_at)	2.08	1.00	1.00	4.14E-37	4.29E-35
KIF2A (203087_s_at)	2.08	1.00	1.00	4.43E-37	4.29E-35
TRIP13 (204033_at)	2.08	1.00	0.98	6.55E-36	1.54E-34
SOX7 (228698_at)	2.08	1.00	1.00	3.45E-37	4.29E-35
UHRF1 (225655_at)	2.08	1.00	1.00	6.28E-36	1.5E-34
AMMECR1 (226421_at)	2.08	1.00	1.00	6.28E-37	4.29E-35
STS (203767_s_at)	2.07	1.00	1.00	8.27E-34	9.22E-33
LGALS3BP (200923_at)	2.07	1.00	1.00	2.09E-36	6.98E-35
TXNDC17 (224511_s_at)	2.06	1.00	1.00	4.5E-37	4.29E-35
BLNK (207655_s_at)	2.06	1.00	1.00	4.19E-37	4.29E-35
FPR1 (205119_s_at)	2.06	0.78	0.23	6.78E-34	7.68E-33
SPIN4 (228654_at)	2.06	1.00	1.00	1.98E-36	6.72E-35
NCAPG (218662_s_at)	2.06	1.00	0.94	4.58E-36	1.19E-34
NCAPH (212949_at)	2.05	0.82	0.59	1.5E-36	5.71E-35
ULBP2 (221291_at)	2.05	0.83	0.64	6.64E-37	4.31E-35
HIGD1A (242317_at)	2.05	1.00	1.00	2.46E-35	4.38E-34
CASC5 (228323_at)	2.05	1.00	0.97	3.04E-36	9E-35
GLT1D1 (229770_at)	2.04	0.93	0.64	3.48E-36	9.78E-35
GINS2 (221521_s_at)	2.04	0.99	0.94	4.46E-36	1.16E-34
TRIM22 (213293_s_at)	2.04	1.00	1.00	7.3E-36	1.67E-34
SLC26A4 (206529_x_at)	2.04	0.95	0.81	2.12E-33	2.14E-32
KIF14 (236641_at)	2.04	0.91	0.74	8.52E-37	4.67E-35
ERCC6L (219650_at)	2.03	0.85	0.33	1.85E-36	6.42E-35
ANGPTL4 (221009_s_at)	2.03	0.96	0.57	9.6E-36	2.06E-34
LOC100288860 (239127_at)	2.03	1.00	0.99	1.49E-35	2.92E-34
HSPA4L (205543_at)	2.03	1.00	1.00	4.19E-37	4.29E-35
XKRX (230349_at)	2.03	0.98	0.95	3.55E-32	2.89E-31
SLC28A3 (232277_at)	2.03	1.00	1.00	7.3E-36	1.67E-34
UCA1 (227919_at)	2.03	0.83	0.58	2.66E-35	4.68E-34
CENPA (204962_s_at)	2.02	1.00	0.99	1.8E-35	3.42E-34
SHROOM2 (204967_at)	2.02	1.00	1.00	1.05E-36	4.96E-35
SH2D2A (207351_s_at)	2.02	0.47	0.06	9.47E-36	2.04E-34
PRC1 (218009_s_at)	2.01	1.00	1.00	1.23E-35	2.52E-34
ASF1B (218115_at)	2.01	0.90	0.74	3.08E-36	9.08E-35

PYCARD (221666_s_at)	2.01	1.00	0.99	1.1E-35	2.3E-34
NRBF2 (221803_s_at)	2.01	1.00	1.00	6.82E-37	4.38E-35
GLB1L3 (1569886_a_at)	2.01	1.00	0.98	5.32E-35	8.19E-34
LOC728431 (229678_at)	2.00	0.99	0.93	3.7E-37	4.29E-35
CCL4 (204103_at)	2.00	0.72	0.19	8.49E-36	1.87E-34
DIAPH3 (229097_at)	2.00	1.00	1.00	4.05E-36	1.09E-34
KIF11 (204444_at)	2.00	1.00	1.00	3.3E-36	9.42E-35
CXCR6 (206974_at)	2.00	0.71	0.09	5.62E-37	4.29E-35
KCNK6 (223658_at)	1.99	1.00	1.00	4.82E-37	4.29E-35
DUOXA1 (1555404_a_at)	1.99	0.86	0.68	5.31E-37	4.29E-35
PTAFR (227184_at)	1.99	0.67	0.63	4.5E-37	4.29E-35
AKIRIN2 (223145_s_at)	1.99	1.00	1.00	7.11E-37	4.43E-35
RAB38 (219412_at)	1.99	1.00	1.00	3.65E-37	4.29E-35
RASGRP1 (205590_at)	1.99	1.00	1.00	7.84E-37	4.55E-35
IFNG (210354_at)	1.99	0.72	0.12	1.22E-36	5.24E-35
IRF8 (204057_at)	1.98	1.00	0.99	4.03E-33	3.84E-32
KRT6B (213680_at)	1.98	1.00	1.00	7.71E-36	1.75E-34
SERPINB1 (213572_s_at)	1.98	1.00	1.00	3.53E-36	9.84E-35
HDHD1 (203974_at)	1.98	1.00	1.00	5.03E-37	4.29E-35
CCNE2 (205034_at)	1.98	1.00	1.00	9.47E-36	2.04E-34
POLE2 (205909_at)	1.97	1.00	1.00	4.89E-37	4.29E-35
BATF2 (228439_at)	1.97	0.58	0.02	5.55E-36	1.36E-34
CENPF (207828_s_at)	1.97	1.00	1.00	2.89E-35	4.99E-34
NFE2L3 (204702_s_at)	1.97	1.00	1.00	1.46E-36	5.66E-35
NR4A3 (209959_at)	1.97	0.68	0.62	1.9E-33	1.95E-32
NIPAL4 (230188_at)	1.97	1.00	1.00	1.2E-36	5.21E-35
KIF18A (221258_s_at)	1.97	0.95	0.79	7.51E-36	1.71E-34
NMI (203964_at)	1.97	1.00	1.00	4.43E-37	4.29E-35
CDCA3 (223307_at)	1.96	1.00	0.98	3.25E-36	9.36E-35
CLDN17 (221328_at)	1.96	0.49	0.01	1.54E-33	1.61E-32
C11orf82 (228281_at)	1.96	0.98	0.76	1.52E-36	5.75E-35
CD83 (204440_at)	1.96	1.00	0.98	2.72E-36	8.34E-35
SKA3 (227165_at)	1.96	0.69	0.70	1.11E-36	5.02E-35
MAP3K9 (213927_at)	1.95	1.00	1.00	1.93E-34	2.51E-33
IL12RB1 (1552584_at)	1.95	0.94	0.75	3.01E-35	5.15E-34
S100A2 (204268_at)	1.95	1.00	1.00	2.37E-36	7.66E-35
LOC285628 (232504_at)	1.95	0.89	0.71	4.69E-37	4.29E-35
CD80 (1554519_at)	1.95	0.63	0.05	1.95E-35	3.65E-34
LINC00518 (244829_at)	1.95	0.92	0.74	2.09E-32	1.76E-31
IFITM10 (227863_at)	1.94	0.97	0.75	1.91E-34	2.48E-33
KCTD4 (239787_at)	1.94	0.87	0.89	7.23E-25	2.9E-24
UCK2 (209825_s_at)	1.94	1.00	1.00	1.08E-36	4.97E-35
ACP5 (204638_at)	1.94	0.93	0.82	5.26E-36	1.3E-34
DDX58 (222793_at)	1.94	1.00	0.95	2.66E-35	4.68E-34
SMAGP (209679_s_at)	1.94	1.00	1.00	1.12E-36	5.06E-35
SLC35E4 (1568623_a_at)	1.93	0.95	0.86	4.84E-35	7.62E-34
STIL (205339_at)	1.93	1.00	1.00	5.86E-37	4.29E-35
LOC100131262 (1558152_at)	1.93	0.99	0.92	2.04E-36	6.86E-35
CDC45 (204126_s_at)	1.93	0.51	0.24	1.95E-36	6.65E-35

CD48 (204118_at)	1.93	0.95	0.85	2.69E-30	1.71E-29
NUSAP1 (218039_at)	1.93	1.00	1.00	1.14E-36	5.08E-35
LOC100506776 (239237_at)	1.93	0.84	0.70	3.42E-32	2.79E-31
SULT2B1 (205759_s_at)	1.93	1.00	0.99	7.97E-35	1.15E-33
NUF2 (223381_at)	1.92	1.00	0.93	2.91E-36	8.74E-35
TUBG1 (201714_at)	1.92	1.00	1.00	1.24E-36	5.3E-35
E2F8 (219990_at)	1.92	1.00	0.97	4.14E-33	3.93E-32
PANX1 (204715_at)	1.91	1.00	1.00	3.39E-36	9.62E-35
IRF9 (203882_at)	1.91	1.00	1.00	3.6E-37	4.29E-35
ICOS (210439_at)	1.91	0.67	0.39	1.4E-36	5.54E-35
FAIM3 (221601_s_at)	1.91	0.81	0.47	2.09E-35	3.84E-34
PRSS22 (205847_at)	1.91	0.98	0.75	7.63E-37	4.46E-35
SDCBP2 (233565_s_at)	1.91	1.00	0.99	1.24E-36	5.3E-35
ZWINT (204026_s_at)	1.91	1.00	1.00	4E-36	1.08E-34
ABCG4 (207593_at)	1.91	0.84	0.33	3.6E-37	4.29E-35
RALGPS2 (227224_at)	1.91	1.00	1.00	1.06E-36	4.97E-35
SLC23A2 (209236_at)	1.91	0.97	0.94	4.71E-35	7.45E-34
GPR183 (205419_at)	1.91	0.99	0.97	6.08E-35	9.18E-34
FERMT1 (218796_at)	1.90	1.00	1.00	1.8E-35	3.42E-34
HMOX2 (218120_s_at)	1.90	1.00	1.00	1.03E-36	4.92E-35
SLC27A4 (225779_at)	1.90	1.00	0.96	4.78E-36	1.21E-34
AURKB (209464_at)	1.90	0.92	0.57	3.78E-36	1.03E-34
GINS1 (206102_at)	1.90	1.00	1.00	3.48E-36	9.78E-35
GBP5 (229625_at)	1.89	0.77	0.56	1.63E-36	5.95E-35
DTL (218585_s_at)	1.89	1.00	1.00	6.01E-34	6.9E-33
FAM160A1 (242687_at)	1.89	1.00	1.00	1.4E-36	5.54E-35
PLEK (203471_s_at)	1.89	0.98	0.93	1.71E-33	1.77E-32
FOXM1 (202580_x_at)	1.89	0.76	0.62	9.13E-37	4.76E-35
SRD5A3 (222750_s_at)	1.89	1.00	1.00	3.89E-35	6.38E-34
CNNM4 (218900_at)	1.88	0.96	0.76	3.21E-36	9.29E-35
CD24 (216379_x_at)	1.88	1.00	1.00	5.78E-37	4.29E-35
MFHAS1 (225478_at)	1.88	1.00	1.00	5.1E-37	4.29E-35
ZNF165 (206683_at)	1.88	0.99	0.94	1.05E-33	1.14E-32
MLKL (238025_at)	1.88	1.00	0.99	8.99E-35	1.28E-33
ACTR3C (223874_at)	1.88	0.91	0.68	4.78E-36	1.21E-34
ETHE1 (204034_at)	1.88	1.00	1.00	1.18E-35	2.43E-34
SHCBP1 (219493_at)	1.87	1.00	0.99	1.39E-33	1.46E-32
SUSD4 (219389_at)	1.87	1.00	0.88	7.17E-33	6.56E-32
ACE2 (219962_at)	1.87	0.82	0.33	1.85E-33	1.9E-32
HS3ST3B1 (227361_at)	1.87	1.00	1.00	1.5E-36	5.71E-35
CENPW (226936_at)	1.87	1.00	1.00	3.21E-36	9.29E-35
VSIG10L (238654_at)	1.87	1.00	0.99	5.61E-35	8.57E-34
GABRA4 (208463_at)	1.87	0.82	0.57	4.8E-28	2.45E-27
PLCXD2 (235230_at)	1.86	0.99	0.97	1.14E-33	1.22E-32
SERPINB8 (206034_at)	1.86	1.00	0.89	2.49E-35	4.43E-34
FMO1 (205666_at)	1.86	1.00	1.00	1.1E-27	5.43E-27
LRRC8B (212978_at)	1.86	1.00	1.00	1.11E-36	5.02E-35
PYDC1 (243722_at)	1.86	0.90	0.74	9.19E-28	4.57E-27
RAD51AP1 (204146_at)	1.86	1.00	0.99	1.35E-35	2.71E-34

SQLE (209218_at)	1.86	1.00	1.00	1.85E-36	6.42E-35
RABGGTA (203573_s_at)	1.86	1.00	1.00	6.11E-37	4.29E-35
WDR53 (227814_at)	1.85	1.00	1.00	1.16E-36	5.11E-35
SERPINB12 (1553057_at)	1.85	0.95	0.79	3.09E-25	1.26E-24
CXCL11 (210163_at)	1.85	0.87	0.52	3.5E-30	2.21E-29
FBN2 (203184_at)	1.85	0.81	0.63	7.36E-31	5.03E-30
SFXN1 (218392_x_at)	1.85	0.99	0.95	3.8E-37	4.29E-35
CYB5R4 (219079_at)	1.85	1.00	1.00	2.12E-35	3.89E-34
SPTLC2 (225095_at)	1.85	1.00	1.00	1.75E-36	6.2E-35
NCOA7 (225344_at)	1.84	1.00	1.00	4.56E-37	4.29E-35
CD8A (205758_at)	1.84	0.86	0.74	7.88E-30	4.77E-29
CXCL17 (226960_at)	1.84	0.58	0.19	4.14E-34	4.91E-33
TUBB6 (209191_at)	1.84	1.00	1.00	9.26E-37	4.79E-35
OIP5 (213599_at)	1.84	0.98	0.91	3.89E-35	6.38E-34
LMNB2 (216952_s_at)	1.84	0.91	0.71	4.84E-35	7.62E-34
TUBBP5 (222361_at)	1.84	0.90	0.60	6.11E-36	1.47E-34
GNLY (205495_s_at)	1.84	0.57	0.17	6.29E-33	5.8E-32
P2RY2 (206277_at)	1.84	0.93	0.65	2.97E-35	5.1E-34
HCAR3 (205220_at)	1.84	1.00	1.00	3.3E-34	4.03E-33
GMPPB (219920_s_at)	1.84	1.00	0.92	5.33E-36	1.31E-34
SLPI (203021_at)	1.84	1.00	1.00	1.71E-34	2.26E-33
MXD1 (226275_at)	1.84	1.00	1.00	3.48E-36	9.78E-35
TMEM54 (225536_at)	1.83	1.00	1.00	1.26E-35	2.58E-34
AMPD3 (207992_s_at)	1.83	1.00	1.00	2.76E-36	8.4E-35
ERVMER34-1 (219987_at)	1.83	0.57	0.24	6.43E-34	7.33E-33
AGPAT9 (224480_s_at)	1.83	1.00	1.00	9.19E-33	8.25E-32
SERTAD1 (223394_at)	1.83	1.00	1.00	8.76E-37	4.74E-35
FKBPL (219187_at)	1.83	1.00	0.89	1.44E-36	5.62E-35
GFOD2 (214076_at)	1.83	0.99	0.95	5.03E-37	4.29E-35
NDRG4 (209159_s_at)	1.83	0.98	0.93	9.52E-37	4.79E-35
SPRYD4 (225616_at)	1.83	1.00	1.00	1.47E-35	2.89E-34
FAM54A (228069_at)	1.83	1.00	0.97	1.73E-35	3.31E-34
CHEK1 (238075_at)	1.83	1.00	0.99	1.63E-35	3.16E-34
BCL3 (204908_s_at)	1.83	1.00	0.98	1.3E-35	2.63E-34
ECE2 (227103_s_at)	1.83	0.95	0.75	9.73E-36	2.08E-34
APOL6 (1557116_at)	1.83	1.00	1.00	4.4E-36	1.15E-34
CSF2RA (210340_s_at)	1.82	0.83	0.43	3.18E-35	5.37E-34
ADH7 (210505_at)	1.82	0.91	0.58	9.39E-37	4.79E-35
PSORS1C2 (220635_at)	1.82	0.94	0.87	4.8E-26	2.08E-25
NCAPH2 (40640_at)	1.82	0.82	0.66	7.96E-33	7.21E-32
MYD88 (209124_at)	1.82	1.00	1.00	5.7E-37	4.29E-35
SERPINB7 (206421_s_at)	1.82	1.00	1.00	2.31E-26	1.03E-25
TPRXL (239061_at)	1.82	0.71	0.61	2.47E-36	7.84E-35
TYMS (202589_at)	1.82	1.00	1.00	3.45E-35	5.78E-34
PTGER3 (213933_at)	1.81	1.00	1.00	2.2E-33	2.22E-32
SLMO2 (222441_x_at)	1.81	1.00	1.00	1.08E-36	4.97E-35
SNX20 (228869_at)	1.81	0.99	0.90	2.93E-34	3.63E-33
UCHL3 (204616_at)	1.81	1.00	1.00	4.14E-37	4.29E-35
FAM40B (231880_at)	1.81	0.76	0.54	9.24E-35	1.31E-33

CDC25A (204695_at)	1.81	0.62	0.60	9.87E-36	2.1E-34
PARP14 (224701_at)	1.81	0.99	0.94	7.31E-37	4.43E-35
NAPSB (228055_at)	1.80	0.81	0.43	2.7E-34	3.38E-33
KRT78 (1553212_at)	1.80	1.00	1.00	4.69E-28	2.4E-27
CDCA8 (221520_s_at)	1.80	0.94	0.67	2.3E-35	4.14E-34
SASH3 (204923_at)	1.80	1.00	0.90	6.03E-32	4.74E-31
SKA1 (217640_x_at)	1.80	0.90	0.59	2.79E-36	8.51E-35
PPARGC1B (232181_at)	1.80	1.00	0.99	7.11E-36	1.64E-34
LRRC59 (222231_s_at)	1.80	1.00	1.00	1.35E-36	5.42E-35
TMEM206 (222752_s_at)	1.80	1.00	0.99	6.92E-37	4.38E-35
COTL1 (224583_at)	1.80	1.00	1.00	6.81E-33	6.24E-32
ITK (211339_s_at)	1.80	1.00	0.97	4.85E-30	3.02E-29
LRRC20 (218550_s_at)	1.80	0.89	0.66	3.12E-36	9.13E-35
PLCD4 (224505_s_at)	1.79	0.76	0.65	1.21E-34	1.66E-33
PXMP4 (219428_s_at)	1.79	1.00	1.00	8.53E-32	6.54E-31
GCH1 (204224_s_at)	1.79	1.00	1.00	4.19E-37	4.29E-35
E2F7 (228033_at)	1.79	0.99	0.93	2.46E-35	4.38E-34
DDHD1 (225970_at)	1.79	1.00	1.00	7.31E-32	5.66E-31
GZMK (206666_at)	1.79	0.87	0.73	9.84E-27	4.49E-26
HJURP (218726_at)	1.79	0.69	0.62	7.82E-36	1.76E-34
IRF1 (202531_at)	1.79	1.00	1.00	3.78E-36	1.03E-34
KCNK10 (220727_at)	1.79	0.85	0.15	4.56E-37	4.29E-35
DDX39A (201584_s_at)	1.79	1.00	1.00	1.9E-36	6.5E-35
MAPKAPK3 (202788_at)	1.79	1.00	1.00	9.01E-37	4.75E-35
UBE2S (202779_s_at)	1.78	0.88	0.52	1.31E-35	2.65E-34
CASP7 (207181_s_at)	1.78	1.00	1.00	6.37E-37	4.29E-35
RNASEH1 (218497_s_at)	1.78	1.00	1.00	6.55E-37	4.29E-35
SHB (204657_s_at)	1.78	0.84	0.70	2.44E-36	7.77E-35
ESRP2 (219395_at)	1.78	1.00	1.00	9.13E-37	4.76E-35
SLAMF8 (219386_s_at)	1.78	1.00	0.97	1.57E-27	7.65E-27
BLM (205733_at)	1.78	0.84	0.66	3.63E-36	1E-34
BRIP1 (235609_at)	1.77	1.00	0.95	2.52E-35	4.46E-34
RAD51 (205024_s_at)	1.77	0.96	0.75	2.42E-35	4.34E-34
FUT1 (206109_at)	1.77	0.99	0.92	4.98E-36	1.25E-34
FPR3 (230422_at)	1.77	0.99	0.96	8.9E-31	6.01E-30
MREG (219648_at)	1.77	1.00	1.00	3.48E-36	9.78E-35
SGOL2 (230165_at)	1.77	1.00	0.97	5.1E-35	7.95E-34
SLAMF1 (206181_at)	1.77	0.95	0.63	1.95E-35	3.65E-34
PNPT1 (225291_at)	1.77	1.00	1.00	5.46E-35	8.38E-34
SLC39A2 (220413_at)	1.77	1.00	1.00	5.51E-32	4.37E-31
SIRPG (220485_s_at)	1.77	0.46	0.14	2.63E-34	3.29E-33
MALL (209373_at)	1.77	1.00	1.00	5.4E-36	1.33E-34
KBTBD8 (239835_at)	1.77	0.97	0.92	1.15E-35	2.38E-34
CD177 (219669_at)	1.77	0.69	0.24	2.68E-36	8.28E-35
TRIM69 (1568592_at)	1.76	1.00	1.00	1.87E-36	6.46E-35
FYB (227266_s_at)	1.76	0.99	0.99	1.29E-29	7.67E-29
LINC00673 (227452_at)	1.76	1.00	1.00	2.2E-32	1.85E-31
KIF15 (219306_at)	1.76	0.91	0.75	1.21E-34	1.66E-33
SNX10 (218404_at)	1.76	1.00	0.99	1.65E-30	1.08E-29

TLR2 (204924_at)	1.76	0.98	0.93	3.73E-33	3.58E-32
IL12RB2 (206999_at)	1.76	0.98	0.71	2.4E-36	7.7E-35
BIRC3 (210538_s_at)	1.76	1.00	0.99	2.73E-31	1.98E-30
PPIL1 (222500_at)	1.76	1.00	1.00	2.7E-35	4.73E-34
CD28 (206545_at)	1.76	0.94	0.80	1.84E-31	1.36E-30
CDC25B (201853_s_at)	1.76	1.00	1.00	8.29E-37	4.63E-35
PPARD (37152_at)	1.75	1.00	1.00	5.54E-37	4.29E-35
RPP40 (213427_at)	1.75	1.00	1.00	2.21E-36	7.28E-35
ALAS1 (205633_s_at)	1.75	1.00	1.00	5.04E-35	7.87E-34
LINC00592 (1558195_at)	1.75	0.87	0.73	7.14E-28	3.59E-27
CATSPERB (220293_at)	1.75	0.73	0.43	2.01E-32	1.7E-31
DTX3L (225415_at)	1.75	1.00	1.00	3.97E-37	4.29E-35
SQRDL (217995_at)	1.75	1.00	1.00	5.94E-37	4.29E-35
ACOT7 (208002_s_at)	1.75	1.00	1.00	1.2E-36	5.21E-35
SPAG5 (203145_at)	1.75	1.00	0.92	5.19E-36	1.29E-34
TIMELESS (203046_s_at)	1.75	1.00	1.00	1.82E-36	6.36E-35
TAP2 (225973_at)	1.75	1.00	1.00	5.62E-37	4.29E-35
P2RY1 (207455_at)	1.75	1.00	0.99	6.99E-33	6.4E-32
IL2RG (204116_at)	1.75	0.99	0.95	9.81E-33	8.78E-32
HEATR3 (219289_at)	1.75	1.00	1.00	9.65E-37	4.82E-35
GBP3 (223434_at)	1.74	1.00	1.00	4.14E-34	4.91E-33
BDH1 (211715_s_at)	1.74	1.00	1.00	2.85E-35	4.94E-34
TRIM16 (204341_at)	1.74	1.00	1.00	2.24E-34	2.85E-33
PRRG4 (238513_at)	1.74	1.00	1.00	1.12E-35	2.32E-34
LMNB1 (203276_at)	1.74	0.99	0.94	2.89E-34	3.59E-33
TNFSF10 (202688_at)	1.74	1.00	1.00	2.57E-36	8.05E-35
IL4I1 (230966_at)	1.74	0.52	0.08	7.82E-36	1.76E-34
SRM (201516_at)	1.74	1.00	1.00	1.73E-35	3.31E-34
REL (206036_s_at)	1.74	1.00	1.00	1.85E-26	8.29E-26
ANKRD22 (238439_at)	1.74	1.00	1.00	1.42E-34	1.92E-33
GPR65 (214467_at)	1.73	0.99	0.91	4.55E-31	3.19E-30
SNHG3 (215011_at)	1.73	0.95	0.88	4.49E-34	5.29E-33
WARS (200629_at)	1.73	1.00	1.00	4.58E-35	7.27E-34
MAPK13 (210059_s_at)	1.73	1.00	1.00	3.44E-36	9.72E-35
AZGP1P1 (217013_at)	1.73	0.50	0.16	1.21E-33	1.3E-32
SH3GL3 (205637_s_at)	1.73	0.88	0.63	5.72E-32	4.52E-31
ANLN (222608_s_at)	1.72	1.00	1.00	1.63E-30	1.07E-29
PTPN22 (206060_s_at)	1.72	0.62	0.19	2.75E-33	2.72E-32
SLC31A1 (203971_at)	1.72	1.00	1.00	6.51E-34	7.42E-33
KCTD11 (235857_at)	1.72	1.00	0.99	3.67E-34	4.43E-33
FSCN1 (201564_s_at)	1.72	1.00	0.99	1.21E-35	2.5E-34
CSTB (201201_at)	1.72	1.00	1.00	2.18E-36	7.22E-35
RDH12 (242998_at)	1.72	1.00	0.99	2.72E-36	8.34E-35
TMEM79 (223544_at)	1.72	1.00	1.00	2.47E-36	7.84E-35
ELOVL7 (227180_at)	1.72	1.00	1.00	8.97E-36	1.95E-34
PARPBP (227928_at)	1.72	0.94	0.76	3.64E-35	6.04E-34
CSF2RB (205159_at)	1.72	1.00	1.00	9.62E-35	1.36E-33
RCC1 (206499_s_at)	1.72	1.00	0.99	5.04E-35	7.87E-34
CENPK (222848_at)	1.72	1.00	0.99	6.43E-34	7.33E-33

PCSK9 (227759_at)	1.72	0.91	0.65	1.73E-36	6.15E-35
MCM5 (216237_s_at)	1.72	1.00	1.00	7.96E-33	7.21E-32
CPSF2 (225994_at)	1.71	1.00	1.00	1.04E-35	2.19E-34
CKAP2L (229610_at)	1.71	0.63	0.59	5.1E-35	7.95E-34
TTC22 (235651_at)	1.71	1.00	1.00	1.09E-36	4.99E-35
NME1 (201577_at)	1.71	1.00	1.00	5.39E-35	8.28E-34
LAD1 (203287_at)	1.71	1.00	1.00	1.09E-36	4.99E-35
FAM111B (1557129_a_at)	1.71	0.91	0.81	1.96E-32	1.66E-31
IQGAP3 (229538_s_at)	1.71	0.99	0.95	3.87E-34	4.65E-33
FANCI (213007_at)	1.71	1.00	1.00	5.33E-36	1.31E-34
TTC9 (213174_at)	1.70	0.86	0.69	3.12E-36	9.13E-35
IFI30 (201422_at)	1.70	1.00	1.00	4.34E-35	6.96E-34
CD53 (203416_at)	1.70	1.00	1.00	7.7E-32	5.94E-31
SLC2A1 (201250_s_at)	1.70	1.00	1.00	2.33E-35	4.19E-34
CYP2E1 (209975_at)	1.70	1.00	1.00	6.27E-32	4.9E-31
NAA15 (222837_s_at)	1.70	1.00	1.00	1.42E-30	9.4E-30
GYS1 (201673_s_at)	1.70	1.00	1.00	4.71E-36	1.21E-34
TMEM117 (223594_at)	1.70	1.00	1.00	6.46E-37	4.29E-35
RAN (200750_s_at)	1.70	1.00	1.00	1.01E-36	4.88E-35
COL6A6 (230867_at)	1.69	0.97	0.87	7.66E-16	1.92E-15
RMND5A (212479_s_at)	1.69	1.00	1.00	3.21E-34	3.94E-33
PSMB10 (202659_at)	1.69	0.99	0.90	2.09E-35	3.84E-34
PGD (201118_at)	1.69	1.00	1.00	4.08E-33	3.89E-32
OVOL1 (229396_at)	1.69	1.00	1.00	4.73E-34	5.55E-33
LRR1 (235113_at)	1.69	1.00	1.00	2.36E-34	2.99E-33
PSMA5 (201274_at)	1.69	1.00	1.00	2.89E-34	3.59E-33
MRPL50 (225580_at)	1.69	1.00	1.00	7.06E-35	1.04E-33
TIMM13 (218188_s_at)	1.69	1.00	1.00	6.82E-36	1.59E-34
PRELID1 (224232_s_at)	1.69	1.00	1.00	1.44E-36	5.62E-35
ZNF518B (226909_at)	1.68	1.00	1.00	6.19E-37	4.29E-35
ABCG1 (204567_s_at)	1.68	1.00	1.00	1.88E-32	1.61E-31
GSDMC (234305_s_at)	1.68	1.00	1.00	1.63E-36	5.95E-35
CASP4 (209310_s_at)	1.68	1.00	1.00	6.55E-37	4.29E-35
C11orf75 (219806_s_at)	1.68	1.00	1.00	6.27E-32	4.9E-31
WHSC1 (209053_s_at)	1.68	1.00	0.96	6.78E-35	1E-33
TNFRSF4 (208023_at)	1.68	0.64	0.62	1.76E-32	1.51E-31
SLC16A1 (209900_s_at)	1.68	1.00	1.00	1.57E-36	5.8E-35
AP2S1 (208074_s_at)	1.68	1.00	1.00	6.19E-32	4.85E-31
SPOCK2 (202524_s_at)	1.68	0.98	0.87	3.93E-34	4.7E-33
BIK (205780_at)	1.68	0.79	0.54	3.63E-36	1E-34
CCDC64B (235095_at)	1.68	1.00	1.00	5.32E-35	8.19E-34
EXO1 (204603_at)	1.68	0.91	0.58	3.44E-36	9.72E-35
IL24 (206569_at)	1.67	0.63	0.55	3.58E-34	4.33E-33
PGLYRP3 (1553059_at)	1.67	0.69	0.48	6.6E-34	7.5E-33
ASCC3 (212815_at)	1.67	1.00	1.00	9.65E-37	4.82E-35
POLB (203616_at)	1.67	1.00	1.00	4.89E-37	4.29E-35
IDH3A (202070_s_at)	1.67	1.00	1.00	6.17E-35	9.28E-34
PUS10 (229362_at)	1.67	0.94	0.92	4.11E-36	1.1E-34
CARD14 (220598_at)	1.67	0.57	0.19	2.4E-36	7.7E-35

KIAA1609 (221843_s_at)	1.67	0.97	0.94	4.4E-36	1.15E-34
OLFM1 (213131_at)	1.67	1.00	1.00	2.15E-34	2.75E-33
EIF4A3 (201303_at)	1.67	1.00	1.00	7.63E-37	4.46E-35
IFRD2 (209100_at)	1.67	1.00	0.99	3.89E-35	6.38E-34
RAC2 (213603_s_at)	1.67	1.00	0.99	9.57E-34	1.05E-32
ODF3B (238327_at)	1.67	1.00	1.00	1.19E-30	7.94E-30
LTB (207339_s_at)	1.67	0.91	0.77	8.72E-34	9.65E-33
ZNF323 (222016_s_at)	1.67	1.00	0.98	3.27E-33	3.18E-32
VCP (208649_s_at)	1.67	1.00	1.00	1.06E-36	4.97E-35
C7orf57 (1557636_a_at)	1.67	0.72	0.46	4.14E-34	4.91E-33
UGCG (224967_at)	1.67	1.00	1.00	9.01E-37	4.75E-35
FCRLB (238452_at)	1.66	0.76	0.42	1.32E-32	1.16E-31
PRMT6 (223275_at)	1.66	1.00	0.99	4.52E-35	7.19E-34
RAB31 (217764_s_at)	1.66	1.00	1.00	1.73E-36	6.15E-35
GART (212378_at)	1.66	1.00	1.00	1.03E-36	4.92E-35
LOC100506100 (228773_at)	1.66	0.95	0.81	1.46E-35	2.89E-34
HMGB3P1 (216548_x_at)	1.66	0.88	0.70	8.37E-36	1.86E-34
JAK3 (227677_at)	1.66	1.00	0.96	5.26E-34	6.13E-33
PLK1 (202240_at)	1.66	0.82	0.46	3.73E-36	1.02E-34
DUSP14 (203367_at)	1.66	1.00	1.00	7.11E-36	1.64E-34
CCL7 (208075_s_at)	1.66	0.57	0.04	5.12E-36	1.28E-34
PYGL (202990_at)	1.66	1.00	1.00	1.8E-35	3.42E-34
DBF4 (204244_s_at)	1.66	1.00	1.00	1.61E-35	3.13E-34
EIF6 (210213_s_at)	1.66	1.00	1.00	4.52E-35	7.19E-34
TRIM62 (58308_at)	1.65	1.00	1.00	2.95E-36	8.82E-35
CCL19 (210072_at)	1.65	1.00	1.00	8.74E-19	2.5E-18
C5orf20 (239529_at)	1.65	0.88	0.62	3.33E-32	2.72E-31
FAM83F (235269_at)	1.65	0.76	0.37	1.3E-35	2.63E-34
CCDC109B (218802_at)	1.65	1.00	1.00	6.55E-37	4.29E-35
DOK3 (223553_s_at)	1.65	0.96	0.93	6.82E-31	4.69E-30
ZNF557 (220444_at)	1.65	0.98	0.88	7.87E-37	4.55E-35
C17orf109 (229740_at)	1.65	1.00	0.93	3.29E-30	2.08E-29
SLC16A9 (227506_at)	1.65	0.69	0.42	1.97E-30	1.28E-29
IL18RAP (207072_at)	1.65	0.62	0.49	3.99E-32	3.22E-31
JUNB (201473_at)	1.65	1.00	0.97	4.34E-35	6.96E-34
GTF3C6 (225083_at)	1.65	1.00	1.00	4.43E-37	4.29E-35
METTL8 (1554667_s_at)	1.65	1.00	1.00	5.55E-36	1.36E-34
PPP4R1 (201594_s_at)	1.65	1.00	1.00	5.17E-37	4.29E-35
IKBKE (204549_at)	1.65	0.98	0.82	2.4E-36	7.7E-35
RUVBL1 (201614_s_at)	1.65	1.00	1.00	1.68E-35	3.24E-34
LTB4R (210128_s_at)	1.65	1.00	1.00	1.42E-34	1.92E-33
DNAJC5 (224612_s_at)	1.64	1.00	1.00	1.27E-36	5.3E-35
ZWILCH (222606_at)	1.64	1.00	1.00	8.72E-36	1.92E-34
CORO2A (227177_at)	1.64	1.00	1.00	8E-32	6.15E-31
CCR5 (206991_s_at)	1.64	0.91	0.66	2.26E-33	2.27E-32
FEN1 (204767_s_at)	1.64	1.00	1.00	8.97E-36	1.95E-34
AVEN (219366_at)	1.64	1.00	1.00	1.27E-36	5.3E-35
GPRIN1 (227975_at)	1.64	0.46	0.03	7.54E-34	8.46E-33
CARHSP1 (224910_at)	1.64	1.00	1.00	5.87E-36	1.42E-34

GPR171 (207651_at)	1.64	0.88	0.69	3.21E-30	2.03E-29
POC1A (226355_at)	1.64	0.59	0.46	3.63E-36	1E-34
CALML5 (220414_at)	1.63	1.00	1.00	1.81E-27	8.79E-27
LCP2 (205269_at)	1.63	1.00	1.00	1.97E-30	1.28E-29
CD3G (206804_at)	1.63	0.71	0.61	1.79E-29	1.04E-28
BAK1 (203728_at)	1.63	0.88	0.63	3.74E-35	6.19E-34
ENDOU (206605_at)	1.63	1.00	1.00	1.7E-29	9.95E-29
ST8SIA4 (230836_at)	1.63	1.00	0.99	1.54E-28	8.28E-28
FBXO46 (205310_at)	1.63	1.00	1.00	6.92E-37	4.38E-35
PMM2 (203201_at)	1.63	1.00	1.00	3.94E-36	1.07E-34
P2RY8 (229686_at)	1.63	0.97	0.81	8.46E-31	5.73E-30
FOXRED2 (231846_at)	1.63	0.99	0.86	1.03E-36	4.92E-35
GGCT (215380_s_at)	1.63	1.00	1.00	4.64E-35	7.36E-34
STK17A (202693_s_at)	1.63	1.00	1.00	1.39E-35	2.77E-34
KLHL18 (212882_at)	1.63	1.00	1.00	2.57E-36	8.05E-35
LMO7 (242722_at)	1.63	1.00	0.98	2.95E-25	1.21E-24
POR (208928_at)	1.63	0.91	0.67	4.98E-36	1.25E-34
KCNJ2 (206765_at)	1.63	1.00	1.00	3.69E-35	6.12E-34
CTSC (225646_at)	1.63	1.00	1.00	8.76E-35	1.25E-33
NUDT5 (223100_s_at)	1.62	1.00	1.00	5.78E-37	4.29E-35
HSPH1 (206976_s_at)	1.62	1.00	1.00	3.26E-34	3.99E-33
SLC37A2 (238638_at)	1.62	0.98	0.94	9.3E-28	4.62E-27
MRPL12 (203931_s_at)	1.62	1.00	0.99	3.73E-33	3.58E-32
RGS18 (223809_at)	1.62	0.92	0.81	1.87E-23	6.83E-23
CACNB4 (207693_at)	1.62	0.74	0.51	6.46E-33	5.94E-32
BORA (219544_at)	1.62	1.00	1.00	2.27E-36	7.46E-35
CYCS (208905_at)	1.62	1.00	1.00	1.52E-36	5.75E-35
C19orf66 (53720_at)	1.62	1.00	1.00	1.62E-33	1.69E-32
RPS6KA4 (204632_at)	1.62	1.00	0.98	9.6E-36	2.06E-34
LOC100506714 (240233_at)	1.62	0.64	0.62	1.52E-36	5.75E-35
IL21R (237753_at)	1.62	0.92	0.53	9.08E-34	1E-32
USP6NL (204761_at)	1.62	1.00	1.00	1.69E-34	2.23E-33
C7orf29 (227598_at)	1.61	1.00	0.94	6.64E-36	1.56E-34
STEAP4 (225987_at)	1.61	1.00	1.00	1.46E-31	1.09E-30
ARL5B (226345_at)	1.61	1.00	1.00	2.83E-36	8.6E-35
CYB5R2 (220230_s_at)	1.61	1.00	1.00	2.81E-35	4.89E-34
LGALS2 (208450_at)	1.61	0.95	0.82	3.82E-28	1.96E-27
AEN (219361_s_at)	1.61	0.63	0.53	3.53E-36	9.84E-35
PLGRKT (218992_at)	1.61	1.00	1.00	8.3E-35	1.19E-33
DFNA5 (203695_s_at)	1.61	0.99	0.94	3.64E-26	1.59E-25
AHCY (200903_s_at)	1.61	1.00	1.00	7.52E-37	4.46E-35
NPM3 (205129_at)	1.61	0.96	0.81	3.21E-34	3.94E-33
PSME2 (201762_s_at)	1.61	1.00	1.00	2.34E-36	7.59E-35
RPL22L1 (225541_at)	1.60	1.00	1.00	7.93E-36	1.78E-34
GOT1 (208813_at)	1.60	1.00	1.00	1.54E-34	2.06E-33
SESN2 (223196_s_at)	1.60	0.94	0.80	2.09E-35	3.84E-34
HERC5 (219863_at)	1.60	1.00	0.99	1.83E-30	1.19E-29
CD3E (205456_at)	1.60	0.83	0.69	6.01E-31	4.15E-30
CCR2 (206978_at)	1.60	1.00	0.97	2.94E-22	1.01E-21

WDR12 (218512_at)	1.60	1.00	1.00	1.31E-36	5.35E-35
PSMC4 (201252_at)	1.60	1.00	1.00	2.44E-36	7.77E-35
METTL6 (1553689_s_at)	1.60	1.00	0.99	1.29E-34	1.76E-33
G6PD (202275_at)	1.60	0.88	0.71	6.33E-29	3.51E-28
H2AFX (213344_s_at)	1.60	0.98	0.97	4.42E-32	3.55E-31
SLCO4A1 (219911_s_at)	1.60	1.00	0.96	1.35E-30	8.95E-30
APEX2 (204408_at)	1.60	1.00	0.95	1.35E-36	5.42E-35
DDX52 (210320_s_at)	1.59	1.00	1.00	1.98E-36	6.72E-35
ADAM19 (209765_at)	1.59	0.94	0.80	1.59E-32	1.37E-31
PLCD1 (205125_at)	1.59	0.98	0.81	2.72E-36	8.34E-35
SP110 (208012_x_at)	1.59	1.00	1.00	3.04E-36	9E-35
IFI16 (208966_x_at)	1.59	1.00	1.00	4.19E-37	4.29E-35
CCDC19 (220308_at)	1.59	0.69	0.51	2.21E-36	7.28E-35
PRF1 (214617_at)	1.59	0.99	0.94	2.58E-29	1.48E-28
CYTIP (209606_at)	1.59	1.00	0.99	1.49E-28	7.99E-28
NAV3 (204823_at)	1.59	0.97	0.90	6.03E-32	4.74E-31
ST14 (202005_at)	1.59	1.00	0.98	1.8E-35	3.42E-34
CXCL6 (206336_at)	1.59	0.66	0.12	3.98E-33	3.8E-32
PVT1 (1558290_a_at)	1.59	1.00	0.91	7.93E-36	1.78E-34
BOP1 (212563_at)	1.59	0.95	0.86	4.43E-34	5.23E-33
CHRNA3 (210221_at)	1.59	0.72	0.62	1.66E-29	9.71E-29
IL17F (234408_at)	1.59	0.36	0.01	6.29E-33	5.8E-32
RUNDC3A (206196_s_at)	1.59	0.64	0.44	2.33E-34	2.95E-33
PKP3 (209873_s_at)	1.58	1.00	1.00	5.54E-35	8.47E-34
CCNA2 (213226_at)	1.58	1.00	1.00	9.12E-35	1.29E-33
CLPB (221845_s_at)	1.58	0.88	0.67	3.18E-35	5.37E-34
ITPKC (213076_at)	1.58	1.00	1.00	8.72E-34	9.65E-33
TMEM51 (218815_s_at)	1.58	0.98	0.94	9.79E-37	4.83E-35
TACC3 (218308_at)	1.58	0.82	0.51	1.19E-34	1.64E-33
TOP1 (208901_s_at)	1.58	1.00	1.00	8.52E-35	1.22E-33
SH2D1A (210116_at)	1.58	0.73	0.46	4.91E-33	4.61E-32
CIB2 (205008_s_at)	1.58	0.90	0.71	7.41E-32	5.73E-31
ESCO2 (235588_at)	1.58	0.69	0.57	2.12E-34	2.72E-33
PSMD11 (208777_s_at)	1.58	1.00	1.00	1.55E-35	3.03E-34
TP53I3 (210609_s_at)	1.58	1.00	0.91	7.71E-36	1.75E-34
MCM2 (202107_s_at)	1.58	1.00	1.00	1.54E-34	2.06E-33
PUSL1 (228733_at)	1.58	1.00	0.81	4.06E-35	6.61E-34
DENND2D (221081_s_at)	1.58	1.00	0.98	3.21E-36	9.29E-35
PARP12 (218543_s_at)	1.58	1.00	1.00	6.03E-36	1.45E-34
TMEM185B (219253_at)	1.57	1.00	1.00	8.3E-35	1.19E-33
ATOX1 (203454_s_at)	1.57	1.00	1.00	2.27E-34	2.88E-33
MAPK6 (207121_s_at)	1.57	1.00	1.00	6.37E-37	4.29E-35
CD69 (209795_at)	1.57	0.96	0.89	1.43E-26	6.46E-26
SNAP29 (218327_s_at)	1.57	1.00	1.00	1.2E-36	5.21E-35
CDC25C (205167_s_at)	1.57	0.87	0.53	5.93E-34	6.82E-33
DCUN1D5 (223151_at)	1.57	1.00	1.00	6.91E-36	1.6E-34
NCBP1 (209520_s_at)	1.57	1.00	1.00	4.03E-33	3.84E-32
TROAP (1568596_a_at)	1.57	0.70	0.55	2.38E-33	2.38E-32
FLJ45445 (225899_x_at)	1.57	1.00	1.00	9.2E-34	1.01E-32

CWH43 (220723_s_at)	1.57	1.00	1.00	1.92E-35	3.62E-34
EHF (225645_at)	1.57	1.00	1.00	3.91E-37	4.29E-35
MCM10 (223570_at)	1.57	0.91	0.81	8.41E-35	1.21E-33
PRMT3 (213320_at)	1.57	1.00	1.00	1.09E-34	1.51E-33
CTSD (200766_at)	1.57	1.00	1.00	1.06E-34	1.48E-33
TSTA3 (36936_at)	1.57	1.00	1.00	2.97E-35	5.1E-34
HYLS1 (227687_at)	1.56	1.00	0.97	1.85E-35	3.5E-34
FAAH2 (230792_at)	1.56	1.00	0.97	1.34E-32	1.17E-31
API5 (214960_at)	1.56	1.00	0.99	3.04E-36	9E-35
NCF2 (209949_at)	1.56	1.00	0.98	1.9E-33	1.95E-32
GPR158 (232195_at)	1.56	0.55	0.33	4.85E-30	3.02E-29
COMTD1 (226870_at)	1.56	1.00	0.94	6.28E-36	1.5E-34
DDX21 (224654_at)	1.56	1.00	1.00	3.34E-36	9.53E-35
ELL2 (226099_at)	1.56	1.00	1.00	2.46E-35	4.38E-34
MYC (202431_s_at)	1.56	1.00	0.99	1.97E-29	1.15E-28
IL32 (203828_s_at)	1.56	0.52	0.20	2.03E-26	9.09E-26
LYZ (213975_s_at)	1.56	1.00	1.00	7.46E-33	6.8E-32
RELL2 (1564031_a_at)	1.56	0.61	0.62	1.42E-36	5.56E-35
LYSMD2 (226748_at)	1.56	1.00	1.00	1.48E-34	1.99E-33
MFN1 (207098_s_at)	1.56	1.00	1.00	5.39E-37	4.29E-35
GID8 (218448_at)	1.56	1.00	1.00	2.97E-34	3.67E-33
FAM100B (224783_at)	1.55	0.98	0.97	6.73E-36	1.57E-34
MICB (206247_at)	1.55	0.96	0.90	5.29E-30	3.27E-29
IKZF1 (227346_at)	1.55	1.00	0.97	9.61E-27	4.39E-26
FAM84A (225667_s_at)	1.55	1.00	1.00	1.07E-35	2.25E-34
ATP6V0D1 (212041_at)	1.55	1.00	1.00	2.83E-33	2.79E-32
TMPRSS13 (223659_at)	1.55	1.00	0.99	6.82E-31	4.69E-30
SEMA7A (230345_at)	1.55	0.61	0.55	2.26E-35	4.11E-34
PCNA (201202_at)	1.55	1.00	1.00	2.09E-35	3.84E-34
KRT37 (207649_at)	1.55	0.45	0.02	6.38E-33	5.87E-32
RAB24 (225251_at)	1.55	1.00	1.00	2.78E-35	4.83E-34
MCTS1 (218163_at)	1.55	1.00	1.00	1.57E-35	3.06E-34
FUT6 (211465_x_at)	1.55	0.58	0.37	7.08E-33	6.48E-32
TIFAB (236673_at)	1.55	0.76	0.44	2.04E-34	2.63E-33
DUOXA2 (230615_at)	1.55	0.61	0.60	2.56E-30	1.64E-29
PIF1 (228252_at)	1.55	0.79	0.35	2.74E-35	4.78E-34
TFEC (206715_at)	1.55	0.91	0.79	6E-27	2.78E-26
PRKCH (218764_at)	1.55	1.00	1.00	3.53E-36	9.84E-35
NOS2 (210037_s_at)	1.55	0.23	0.01	3.63E-36	1E-34
ZDHHC13 (219296_at)	1.54	1.00	1.00	1.13E-34	1.57E-33
SGOL1 (1553690_at)	1.54	0.91	0.76	1.94E-31	1.42E-30
PSMG1 (203405_at)	1.54	1.00	1.00	5.69E-35	8.66E-34
CDCA7 (224428_s_at)	1.54	1.00	1.00	6.11E-32	4.8E-31
LAP3 (217933_s_at)	1.54	1.00	1.00	3.89E-35	6.38E-34
SEMA4B (234725_s_at)	1.54	1.00	1.00	7.2E-36	1.65E-34
TAP1 (202307_s_at)	1.54	1.00	1.00	4.28E-35	6.91E-34
GALE (202528_at)	1.54	0.98	0.80	4.17E-35	6.76E-34
RHOH (204951_at)	1.54	0.19	0.07	8.59E-29	4.72E-28
STAT3 (208991_at)	1.54	1.00	1.00	3.87E-33	3.71E-32

DNMT1 (201697_s_at)	1.54	1.00	1.00	5.24E-35	8.12E-34
PRR9 (237732_at)	1.54	0.99	0.87	4.25E-10	8.31E-10
PIGW (1558292_s_at)	1.54	1.00	1.00	6.68E-32	5.2E-31
CLSPN (243840_at)	1.54	0.58	0.51	3.6E-32	2.93E-31
DBNL (222429_at)	1.54	1.00	0.98	9.09E-36	1.97E-34
YARS (212048_s_at)	1.54	1.00	1.00	1.57E-36	5.8E-35
ENAH (222433_at)	1.54	1.00	1.00	6.37E-37	4.29E-35
KCTD5 (218474_s_at)	1.54	1.00	1.00	2.99E-36	8.93E-35
ALDH4A1 (203722_at)	1.54	0.95	0.82	1.8E-35	3.42E-34
C1QBP (214214_s_at)	1.54	1.00	1.00	4.09E-34	4.87E-33
ORMDL2 (218556_at)	1.54	1.00	1.00	6.6E-34	7.5E-33
FBXO5 (218875_s_at)	1.54	1.00	1.00	2.83E-33	2.79E-32
ACOT11 (214763_at)	1.54	0.42	0.06	6.46E-36	1.53E-34
MYO1E (203072_at)	1.54	1.00	1.00	2.26E-35	4.11E-34
RNASEH2A (203022_at)	1.54	1.00	0.99	2.24E-34	2.85E-33
PMCH (206942_s_at)	1.54	0.62	0.60	4.48E-32	3.59E-31
EPHA1 (205977_s_at)	1.54	0.95	0.82	4.66E-33	4.39E-32
CKAP4 (200999_s_at)	1.53	1.00	1.00	5.03E-37	4.29E-35
TRIM21 (204804_at)	1.53	0.98	0.96	3.64E-35	6.04E-34
NAT1 (214440_at)	1.53	1.00	1.00	4.11E-35	6.68E-34
MANF (202655_at)	1.53	1.00	1.00	1.26E-34	1.72E-33
TIGIT (240070_at)	1.53	0.57	0.13	5.32E-35	8.19E-34
PSPH (205194_at)	1.53	1.00	1.00	3.74E-35	6.19E-34
TMEM19 (226860_at)	1.53	1.00	1.00	2.59E-35	4.56E-34
SLC25A15 (218653_at)	1.53	0.98	0.98	6.51E-35	9.73E-34
ACOX3 (204241_at)	1.53	1.00	0.99	1.68E-35	3.24E-34
NUDT15 (219347_at)	1.53	1.00	1.00	1.7E-36	6.12E-35
CDC42EP1 (204693_at)	1.53	0.92	0.88	1.44E-33	1.52E-32
TWF1 (201745_at)	1.53	1.00	1.00	7.63E-37	4.46E-35
AGPAT5 (218096_at)	1.53	1.00	1.00	1.12E-33	1.21E-32
FASTKD1 (219002_at)	1.53	1.00	1.00	6.46E-36	1.53E-34
MCM6 (201930_at)	1.53	1.00	1.00	2.3E-34	2.92E-33
SEL1L3 (212314_at)	1.53	1.00	1.00	3.27E-31	2.34E-30
LLPH (224446_at)	1.53	1.00	1.00	4.9E-35	7.69E-34
ODZ2 (231867_at)	1.53	1.00	1.00	4.16E-31	2.94E-30
TLR8 (229560_at)	1.53	0.98	0.92	4.49E-24	1.71E-23
LOC284219 (1556425_a_at)	1.53	0.96	0.78	5.3E-32	4.21E-31
CTSL1 (202087_s_at)	1.53	1.00	1.00	2.57E-32	2.14E-31
CTSB (200838_at)	1.53	1.00	1.00	3.25E-36	9.36E-35
TOMM40L (226059_at)	1.52	0.99	0.91	1.36E-36	5.47E-35
FRMD8 (227964_at)	1.52	1.00	1.00	5.39E-37	4.29E-35
CPA4 (205832_at)	1.52	1.00	1.00	4.26E-28	2.18E-27
SRA1 (224864_at)	1.52	1.00	1.00	1.57E-35	3.06E-34
BYSL (203612_at)	1.52	0.97	0.82	3.13E-34	3.85E-33
CHST11 (226372_at)	1.52	0.97	0.91	6.15E-27	2.85E-26
POLQ (219510_at)	1.52	0.90	0.69	4.67E-34	5.48E-33
FAM162A (223193_x_at)	1.52	1.00	1.00	8.76E-35	1.25E-33
DPP3 (232510_s_at)	1.52	1.00	0.95	1.5E-34	2.01E-33
FKBP1B (206857_s_at)	1.52	1.00	1.00	1.52E-31	1.13E-30

CACNA2D3 (219714_s_at)	1.52	0.74	0.64	6.96E-35	1.03E-33
NDUFAF4 (219006_at)	1.52	1.00	1.00	1.02E-32	9.1E-32
TRMT6 (233970_s_at)	1.52	1.00	1.00	2.35E-33	2.35E-32
PTPRE (221840_at)	1.52	1.00	1.00	1.73E-36	6.15E-35
ORC1 (205085_at)	1.52	0.67	0.21	1.28E-33	1.36E-32
MIR17HG (232291_at)	1.52	0.85	0.71	1.95E-33	1.99E-32
DEDD2 (225434_at)	1.52	1.00	1.00	1.95E-35	3.65E-34
C6orf62 (213872_at)	1.52	1.00	1.00	1.69E-30	1.11E-29
EIF2B2 (202461_at)	1.52	1.00	1.00	4.84E-36	1.23E-34
CD27 (206150_at)	1.52	0.94	0.74	1.73E-27	8.39E-27
AK2 (208967_s_at)	1.52	1.00	1.00	4.58E-35	7.27E-34
SUV39H2 (1554572_a_at)	1.52	0.99	0.98	6.18E-34	7.07E-33
FLJ16734 (243077_at)	1.52	0.86	0.69	6.51E-32	5.08E-31
MIR31HG (1554097_a_at)	1.52	0.75	0.19	5.04E-33	4.72E-32
KCNG3 (1552897_a_at)	1.52	0.76	0.32	1.2E-33	1.28E-32
CCDC167 (225723_at)	1.52	1.00	0.96	7.05E-34	7.97E-33
CCNF (204826_at)	1.52	0.95	0.88	1.81E-34	2.37E-33
ZC3H12C (231899_at)	1.52	1.00	1.00	5.38E-33	5.01E-32
CXCL16 (223454_at)	1.52	1.00	1.00	1.43E-35	2.83E-34
GTPBP2 (221050_s_at)	1.51	0.93	0.84	4.69E-37	4.29E-35
TDP2 (202266_at)	1.51	1.00	1.00	4.22E-35	6.84E-34
SEC23B (201583_s_at)	1.51	1.00	1.00	2.85E-35	4.94E-34
MYO5B (225301_s_at)	1.51	1.00	1.00	5.34E-29	2.98E-28
LOC100134229 (232579_at)	1.51	1.00	1.00	1.15E-32	1.02E-31
FAM3D (227676_at)	1.51	0.60	0.52	2.16E-26	9.62E-26
FBXO10 (227222_at)	1.51	0.64	0.51	2.37E-36	7.66E-35
CLCA2 (217528_at)	1.51	1.00	1.00	4.52E-36	1.18E-34
CASP14 (231722_at)	1.51	0.98	0.91	3.96E-15	9.64E-15
RANBP1 (202483_s_at)	1.51	1.00	1.00	3.64E-28	1.88E-27
EME1 (234464_s_at)	1.51	1.00	1.00	6.96E-34	7.87E-33
MS4A7 (223343_at)	1.51	1.00	0.99	2.52E-21	8.19E-21
UBA6 (222602_at)	1.51	1.00	1.00	9.73E-36	2.08E-34
GTPBP4 (218239_s_at)	1.51	1.00	1.00	8.49E-36	1.87E-34
TMEM30B (213285_at)	1.51	1.00	1.00	1.81E-34	2.37E-33
LIN7C (219399_at)	1.51	1.00	1.00	4.13E-26	1.8E-25
VARS (201797_s_at)	1.51	1.00	0.98	8.16E-34	9.1E-33
NOP2 (214427_at)	1.51	1.00	1.00	2.15E-30	1.39E-29
GEN1 (228286_at)	1.50	1.00	0.96	3.31E-33	3.21E-32
MRPL4 (218105_s_at)	1.50	1.00	0.98	7.05E-34	7.97E-33
ERN1 (235745_at)	1.50	1.00	0.99	1.61E-32	1.39E-31
RPTN (1553454_at)	1.50	1.00	0.99	2.18E-13	4.9E-13
ISG20L2 (212766_s_at)	1.50	1.00	1.00	2.68E-36	8.28E-35
SH2D4A (219749_at)	1.50	0.97	0.93	8.97E-36	1.95E-34
IKZF3 (227030_at)	1.50	0.19	0.06	2.24E-34	2.85E-33
RPL26L1 (218830_at)	1.50	1.00	1.00	6.28E-37	4.29E-35
HMGB3 (203744_at)	1.50	1.00	1.00	3.2E-32	2.62E-31
STYK1 (221696_s_at)	1.50	0.95	0.89	2.12E-36	7.07E-35
TIMM10 (1555764_s_at)	1.50	1.00	1.00	5.24E-33	4.89E-32
GARS (208693_s_at)	1.50	1.00	1.00	7.95E-37	4.55E-35
