# Supplementary material for: Cellular dissection of psoriasis for transcriptome analyses and the post-GWAS era
Source: BMC Med Genomics. 2014 May 22;7:27. doi: 10.1186/1755-8794-7-27 (PMC4060870; doi:10.1186/1755-8794-7-27)
Supplement: Additional file 2 — Genes significantly decreased in psoriasis lesions ( n = 216 patients). The table lists the 885 differentially expressed genes (DEGs) significantly decreased in psoriasis lesions (FC < 0.67, FDR < 0.05). DEGs are sorted according to their median fold-change in lesional skin as compared to uninvolved skin (n = 216 patients). The table lists the frequency with which each gene was detected significantly above background among PP samples (third column) and PN samples (fourth column) (Wilcoxon signed rank test, P < 0.05). P-values for differential expression were calculated using the Wilcoxon rank sum test (fifth column) and FDR-adjusted p-values were calculated using the Benjamini-Hochberg method (final column). [file 1755-8794-7-27-S2.pdf]

Additional File 2. Genes significantly decreased in psoriasis lesions (*n* = 216 patients). The table lists the 885 differentially expressed genes (DEGs) significantly decreased in psoriasis lesions (FC < 0.67, FDR < 0.05). DEGs are sorted according to their median fold-change in lesional skin as compared to uninvolved skin (*n* = 216 patients). The table lists the frequency with which each gene was detected significantly above background among PP samples (third column) and PN samples (fourth column) (Wilcoxon signed rank test, P < 0.05). P-values for differential expression were calculated using the Wilcoxon rank sum test (fifth column) and FDR-adjusted p-values were calculated using the Benjamini-Hochberg method (final column).

**Symbol (Probe ID)**	**FC (PP/PN)**	**Freq (PP)**	**Freq (PN)**	**P-value**	**FDR**
BTC (241412_at)	0.091	0.82	1.00	4.08E-37	4.29E-35
WIF1 (204712_at)	0.124	0.83	0.99	7.42E-37	4.43E-35
THRSP (229476_s_at)	0.155	0.87	0.98	1.47E-30	9.73E-30
CCL27 (207955_at)	0.189	0.95	1.00	4.56E-37	4.29E-35
PM20D1 (239929_at)	0.209	0.64	0.85	2.78E-26	1.23E-25
KRT77 (237120_at)	0.218	1.00	1.00	3.55E-37	4.29E-35
HSD11B1 (205404_at)	0.219	0.99	1.00	1.82E-36	6.36E-35
WDR72 (227174_at)	0.225	0.94	1.00	5.17E-37	4.29E-35
MUC7 (217059_at)	0.238	0.53	0.88	7.86E-35	1.14E-33
MSMB (210297_s_at)	0.239	0.82	1.00	1.68E-36	6.07E-35
IL37 (221470_s_at)	0.24	0.89	0.98	1.19E-31	8.99E-31
FABP7 (205030_at)	0.263	0.99	1.00	1.14E-28	6.19E-28
TPPP (230104_s_at)	0.27	0.90	1.00	4.31E-37	4.29E-35
SCGB2A1 (205979_at)	0.28	0.70	0.92	1.82E-36	6.36E-35
ZBTB16 (205883_at)	0.285	0.76	0.99	4.31E-37	4.29E-35
C5orf46 (1554195_a_at)	0.286	0.90	1.00	8.26E-36	1.84E-34
GAL (214240_at)	0.294	0.43	0.77	7.82E-24	2.93E-23
RORC (228806_at)	0.305	0.10	0.89	6.92E-37	4.38E-35
CLDN8 (214598_at)	0.306	0.96	1.00	3.6E-37	4.29E-35
ZDHHC11 (221646_s_at)	0.315	0.95	1.00	1.27E-36	5.3E-35
TSPAN8 (203824_at)	0.317	0.97	0.99	4.91E-36	1.24E-34
ELOVL3 (234513_at)	0.321	0.44	0.76	7.48E-25	2.99E-24
FGFBP2 (223836_at)	0.327	0.76	0.96	3.3E-36	9.42E-35
PAMR1 (213661_at)	0.332	0.83	1.00	1.09E-36	4.99E-35
HMGCS2 (204607_at)	0.333	0.74	0.94	9.01E-37	4.75E-35
MACROD2 (235278_at)	0.333	0.84	1.00	5.86E-37	4.29E-35
CDHR1 (213369_at)	0.335	0.99	1.00	9.34E-36	2.02E-34
CHP2 (206149_at)	0.337	1.00	1.00	9.62E-35	1.36E-33
AQP9 (205568_at)	0.339	0.87	0.97	1.01E-28	5.5E-28
TNMD (220065_at)	0.344	0.71	0.93	2.93E-34	3.63E-33
HS3ST6 (239547_at)	0.346	0.86	1.00	1.43E-35	2.83E-34
ACADL (206068_s_at)	0.348	0.70	0.98	5.24E-37	4.29E-35
SLC14A1 (229151_at)	0.351	0.57	0.84	3.54E-35	5.91E-34
FA2H (219429_at)	0.352	0.97	1.00	6.35E-32	4.96E-31
SLC1A6 (1554593_s_at)	0.354	0.95	1.00	1.54E-29	9.04E-29
PLIN1 (205913_at)	0.355	0.82	0.91	3.24E-26	1.42E-25
GSTA3 (222102_at)	0.356	0.69	0.97	2.3E-35	4.14E-34
MFSD4 (229254_at)	0.359	0.99	1.00	5.24E-37	4.29E-35
TMEM56 (234980_at)	0.361	0.68	0.90	3.89E-32	3.15E-31

ANKRD33B (231963_at)	0.361	0.36	0.82	3.55E-37	4.29E-35
RAB3B (239202_at)	0.362	0.47	0.81	5.94E-37	4.29E-35
SCGB1D2 (206799_at)	0.375	0.96	0.99	1.42E-34	1.92E-33
PPP1R1A (205478_at)	0.379	0.44	0.81	3.4E-31	2.43E-30
CMAHP (205518_s_at)	0.379	1.00	1.00	2.3E-36	7.54E-35
ENPP5 (227803_at)	0.38	0.88	0.99	5.87E-36	1.42E-34
LEP (207092_at)	0.382	0.54	0.78	2.05E-25	8.49E-25
PIP (206509_at)	0.383	0.96	0.97	1.05E-33	1.14E-32
RBP4 (219140_s_at)	0.384	0.54	0.85	5.1E-30	3.16E-29
H19 (224646_x_at)	0.39	0.78	0.88	8.15E-36	1.82E-34
ATP1A2 (203296_s_at)	0.392	0.97	1.00	1.59E-35	3.09E-34
PAPLN (226435_at)	0.392	1.00	1.00	4.37E-37	4.29E-35
SP8 (237449_at)	0.392	0.56	0.92	1.42E-34	1.92E-33
CLDN23 (228707_at)	0.393	0.77	1.00	3.91E-37	4.29E-35
ACSBG1 (206465_at)	0.395	0.86	0.98	9.08E-28	4.52E-27
CA6 (206873_at)	0.396	0.93	0.99	4.84E-32	3.87E-31
PLLP (204519_s_at)	0.396	1.00	1.00	4.96E-37	4.29E-35
CRAT (209522_s_at)	0.398	0.97	0.99	5.43E-31	3.77E-30
LGR5 (213880_at)	0.398	0.97	0.99	3.95E-31	2.8E-30
PPARGC1A (219195_at)	0.399	0.86	0.97	9.52E-37	4.79E-35
LAMB4 (215516_at)	0.4	1.00	1.00	1.5E-36	5.71E-35
ANKFN1 (1553211_at)	0.4	0.21	0.77	2.46E-35	4.38E-34
LOC100507397 (214945_at)	0.4	0.89	0.99	9.32E-34	1.03E-32
TMEM116 (227172_at)	0.402	0.99	1.00	1.08E-36	4.97E-35
ADIPOQ (207175_at)	0.403	0.96	0.98	6.12E-23	2.18E-22
NR3C2 (205259_at)	0.404	0.98	1.00	7.95E-37	4.55E-35
KIAA1244 (228051_at)	0.404	0.67	0.86	1.57E-36	5.8E-35
PPP1R1B (225165_at)	0.404	0.88	0.98	2.65E-36	8.21E-35
C1QTNF7 (239349_at)	0.411	0.97	1.00	1.26E-34	1.72E-33
BTBD16 (1552566_at)	0.411	0.05	0.49	7.51E-36	1.71E-34
PCP4 (205549_at)	0.412	0.89	0.97	3.24E-32	2.66E-31
AGR3 (228241_at)	0.413	0.06	0.67	3.12E-36	9.13E-35
PDK4 (225207_at)	0.415	0.97	1.00	7.42E-37	4.43E-35
PRR4 (204919_at)	0.415	0.91	0.98	4.66E-31	3.26E-30
SNTB1 (226438_at)	0.416	0.98	1.00	9.01E-37	4.75E-35
LEPR (209894_at)	0.419	1.00	1.00	1.8E-36	6.3E-35
FAM189A2 (213900_at)	0.421	0.98	1.00	4.05E-36	1.09E-34
FAM70A (219895_at)	0.421	0.92	1.00	1.29E-36	5.35E-35
ADAMTSL3 (213974_at)	0.422	0.88	1.00	5.86E-37	4.29E-35
GPD1 (213706_at)	0.423	0.38	0.81	1.99E-30	1.29E-29
PHYHIP (205325_at)	0.423	0.38	0.93	4.75E-37	4.29E-35
ATP6V0A4 (220197_at)	0.424	0.89	0.95	6.6E-35	9.83E-34
CYP4B1 (210096_at)	0.425	0.96	1.00	1.69E-34	2.23E-33
SYT8 (232802_at)	0.425	0.04	0.54	1.31E-36	5.35E-35
AGTR1 (205357_s_at)	0.426	1.00	1.00	1.47E-35	2.89E-34
FADS2 (202218_s_at)	0.426	0.26	0.56	4.48E-26	1.95E-25
PLN (204939_s_at)	0.427	0.97	0.99	7.13E-32	5.52E-31
HIF3A (1555318_at)	0.428	0.70	0.89	4.98E-36	1.25E-34
BCAR3 (204032_at)	0.43	0.98	1.00	4.14E-37	4.29E-35

ZSCAN18 (218312_s_at)	0.43	0.95	1.00	1.54E-36	5.78E-35
APOC1 (204416_x_at)	0.431	0.64	0.76	7.59E-30	4.6E-29
ACTG2 (202274_at)	0.432	1.00	1.00	2.38E-32	1.99E-31
BHLHE41 (221530_s_at)	0.432	0.99	1.00	9.39E-37	4.79E-35
FADS1 (208964_s_at)	0.433	0.99	0.99	1.67E-25	6.97E-25
SERPINA12 (1552544_at)	0.434	1.00	1.00	7.41E-21	2.35E-20
C14orf64 (1559097_at)	0.434	0.74	0.97	4.31E-37	4.29E-35
EPCAM (201839_s_at)	0.435	1.00	1.00	4.34E-36	1.15E-34
SCN7A (228504_at)	0.435	0.91	0.98	7.42E-37	4.43E-35
WFDC3 (232602_at)	0.435	0.45	0.73	1.26E-35	2.58E-34
LPL (203549_s_at)	0.437	0.98	0.99	9.61E-30	5.78E-29
PGM5 (226303_at)	0.437	0.98	1.00	1.42E-36	5.56E-35
LOC284578 (1557474_at)	0.437	0.61	0.76	6.42E-35	9.61E-34
ADRB2 (206170_at)	0.438	1.00	1.00	4.96E-37	4.29E-35
LOC643792 (244065_at)	0.438	0.86	1.00	1.33E-33	1.41E-32
ADH1B (209613_s_at)	0.439	1.00	1.00	1.44E-34	1.94E-33
CRIP1 (205081_at)	0.439	1.00	1.00	3.8E-37	4.29E-35
CNKSR2 (229116_at)	0.439	0.58	0.88	4E-36	1.08E-34
GALNTL2 (228501_at)	0.439	0.97	0.99	2.11E-32	1.79E-31
FAR2 (220615_s_at)	0.441	0.94	0.99	4.42E-27	2.07E-26
C14orf132 (218820_at)	0.441	1.00	1.00	1.63E-36	5.95E-35
IGFL2 (231148_at)	0.441	0.23	0.91	1.81E-29	1.06E-28
RHPN2 (227196_at)	0.442	0.97	0.98	2.09E-33	2.11E-32
KRT79 (1569909_at)	0.442	0.78	0.84	9.29E-21	2.93E-20
RAI2 (219440_at)	0.443	0.98	1.00	9.01E-37	4.75E-35
MYOC (210155_at)	0.444	0.65	0.90	6.03E-32	4.74E-31
RNASE4 (205158_at)	0.445	0.96	1.00	4.22E-36	1.12E-34
ADCY2 (213217_at)	0.446	0.89	1.00	3.53E-36	9.84E-35
IGFBP6 (203851_at)	0.447	0.99	1.00	3.6E-32	2.93E-31
TNNI2 (206393_at)	0.447	0.19	0.69	1.54E-36	5.78E-35
FST (226847_at)	0.447	0.99	1.00	9.47E-36	2.04E-34
SCD5 (224901_at)	0.448	0.95	1.00	3.75E-37	4.29E-35
RERGL (220276_at)	0.45	0.88	0.98	1.91E-34	2.48E-33
PCDH20 (232054_at)	0.451	0.68	0.97	1.55E-35	3.03E-34
NCALD (211685_s_at)	0.452	1.00	1.00	3.18E-35	5.37E-34
TIMP4 (206243_at)	0.453	0.64	0.90	6.26E-29	3.47E-28
PLCB4 (203895_at)	0.455	0.94	0.99	2.36E-35	4.25E-34
C7 (202992_at)	0.456	0.95	0.99	2.3E-35	4.14E-34
CES1 (209616_s_at)	0.457	0.97	1.00	1.65E-34	2.18E-33
PCOLCE2 (219295_s_at)	0.457	1.00	0.99	1.31E-25	5.53E-25
CGNL1 (225817_at)	0.459	0.99	1.00	1.61E-36	5.91E-35
EMX2 (221950_at)	0.461	1.00	1.00	7.02E-37	4.4E-35
F3 (204363_at)	0.462	0.96	1.00	5.24E-37	4.29E-35
CNN1 (203951_at)	0.464	0.73	0.93	1.88E-33	1.92E-32
MYH11 (201497_x_at)	0.464	0.99	1.00	1E-35	2.12E-34
NRN1 (218625_at)	0.464	1.00	1.00	1.87E-36	6.46E-35
CIDEC (219398_at)	0.464	0.97	0.96	1.67E-26	7.49E-26
COBL (213050_at)	0.465	0.78	1.00	3.75E-37	4.29E-35
HAO2 (220801_s_at)	0.466	0.47	0.77	8.37E-23	2.96E-22

ESRRG (207981_s_at)	0.467	0.79	0.93	1.17E-33	1.25E-32
EDIL3 (225275_at)	0.467	1.00	1.00	1.48E-36	5.69E-35
CASQ2 (207317_s_at)	0.468	0.82	0.96	3.94E-32	3.19E-31
CRYAB (209283_at)	0.469	1.00	1.00	4.75E-37	4.29E-35
CYP2J2 (205073_at)	0.469	0.86	0.99	7.15E-35	1.05E-33
PRLR (227629_at)	0.47	0.97	1.00	6.11E-37	4.29E-35
ZNF91 (206059_at)	0.47	1.00	1.00	3.63E-36	1E-34
HSD3B1 (204515_at)	0.471	0.25	0.65	2.6E-24	1.01E-23
CLDN1 (222549_at)	0.471	1.00	1.00	4.56E-37	4.29E-35
RCAN2 (203498_at)	0.471	1.00	1.00	1.4E-36	5.54E-35
MYL9 (201058_s_at)	0.471	0.97	1.00	1.76E-32	1.51E-31
ATP13A5 (243585_at)	0.471	0.67	0.85	1.01E-35	2.14E-34
C2orf40 (223623_at)	0.472	0.96	1.00	6.6E-34	7.5E-33
FIBIN (226769_at)	0.472	0.98	1.00	9.6E-36	2.06E-34
LOC100130476 (243871_at)	0.472	0.97	1.00	1.33E-25	5.59E-25
LOC100996430 (228567_at)	0.472	1.00	1.00	1.1E-34	1.53E-33
GLDC (204836_at)	0.473	0.57	0.76	4.02E-24	1.54E-23
CDH19 (206898_at)	0.473	0.79	0.94	6.73E-36	1.57E-34
CHL1 (204591_at)	0.474	1.00	1.00	2.42E-35	4.34E-34
FOXC1 (213260_at)	0.475	1.00	1.00	1.2E-36	5.21E-35
SGCG (207302_at)	0.476	0.76	0.98	1.24E-32	1.09E-31
TMEM47 (209656_s_at)	0.477	1.00	1.00	1.95E-36	6.65E-35
ACTC1 (205132_at)	0.48	0.85	0.97	4.49E-25	1.82E-24
SSPN (204963_at)	0.48	0.98	1.00	2.52E-35	4.46E-34
CD207 (220428_at)	0.48	1.00	1.00	3.92E-28	2.01E-27
FRZB (203697_at)	0.481	1.00	1.00	7.95E-34	8.89E-33
LMOD1 (203766_s_at)	0.481	0.92	0.99	7.86E-35	1.14E-33
ANG (205141_at)	0.482	0.99	1.00	8.17E-37	4.58E-35
RAI14 (202052_s_at)	0.482	1.00	1.00	3.8E-37	4.29E-35
PCDHB16 (232099_at)	0.482	0.97	1.00	4.65E-36	1.2E-34
PRR15L (219127_at)	0.482	0.74	0.96	1.77E-36	6.25E-35
SLITRK6 (235976_at)	0.484	0.95	1.00	3.89E-35	6.38E-34
ABHD12B (237974_at)	0.484	0.94	1.00	1.8E-23	6.62E-23
RHOBTB3 (225202_at)	0.486	1.00	1.00	7.31E-37	4.43E-35
LOC100131564 (227074_at)	0.486	0.97	1.00	7.21E-37	4.43E-35
CRY2 (212695_at)	0.49	0.63	0.94	4.02E-37	4.29E-35
MAML2 (235457_at)	0.49	1.00	1.00	1.27E-36	5.3E-35
ADRB1 (229309_at)	0.491	0.85	0.95	4.31E-34	5.09E-33
UST (205139_s_at)	0.491	0.99	1.00	1.63E-35	3.16E-34
FABP4 (203980_at)	0.492	0.99	1.00	2.46E-29	1.41E-28
MAMDC2 (228885_at)	0.492	1.00	1.00	1.55E-35	3.03E-34
C9orf152 (229964_at)	0.492	0.90	0.99	8.84E-36	1.94E-34
GXYLT2 (235371_at)	0.492	0.98	1.00	4.38E-29	2.47E-28
SLC8A1 (235518_at)	0.493	0.98	1.00	1.57E-35	3.06E-34
DDAH1 (209094_at)	0.493	1.00	1.00	8.52E-37	4.67E-35
CDO1 (204154_at)	0.494	0.90	0.96	3.19E-31	2.29E-30
ERBB4 (214053_at)	0.494	0.60	0.71	1.18E-34	1.63E-33
ACACB (49452_at)	0.495	0.90	1.00	1.03E-34	1.44E-33
CLEC3B (205200_at)	0.495	0.98	1.00	1.79E-32	1.53E-31

LDB3 (213371_at)	0.495	0.87	0.96	5.48E-34	6.34E-33
SEMA3G (219689_at)	0.495	0.95	1.00	2.23E-35	4.08E-34
MMP28 (219909_at)	0.495	0.85	0.99	5.05E-36	1.26E-34
PRUNE2 (212805_at)	0.495	1.00	1.00	3.31E-33	3.21E-32
RGMB (226989_at)	0.496	1.00	1.00	3.65E-37	4.29E-35
OGN (222722_at)	0.497	0.94	0.98	1.12E-19	3.35E-19
RTN4 (1556049_at)	0.497	0.96	1.00	1.18E-34	1.63E-33
SCARA5 (229839_at)	0.498	0.99	1.00	2.89E-32	2.39E-31
GATA3 (209604_s_at)	0.499	1.00	1.00	1.73E-36	6.15E-35
NRXN3 (229649_at)	0.499	0.61	0.66	1.31E-36	5.35E-35
LRRC17 (205381_at)	0.501	0.96	1.00	1.98E-32	1.68E-31
FXYD6 (217897_at)	0.501	0.94	0.98	4.52E-36	1.18E-34
LOC338667 (1564786_at)	0.502	0.62	0.67	3.42E-32	2.79E-31
SYN2 (210247_at)	0.503	0.83	0.99	6.69E-35	9.94E-34
PTPN21 (226380_at)	0.503	1.00	1.00	1.37E-35	2.75E-34
FRMD3 (230645_at)	0.503	0.98	1.00	2.74E-35	4.78E-34
CORO2B (209789_at)	0.504	0.77	0.94	5.71E-36	1.39E-34
SYNM (212730_at)	0.504	0.98	1.00	3.78E-36	1.03E-34
CFH (213800_at)	0.505	0.99	1.00	1.51E-32	1.31E-31
ITM2A (202746_at)	0.505	1.00	1.00	4.08E-37	4.29E-35
GPRASP1 (204793_at)	0.507	0.97	1.00	1.35E-34	1.83E-33
CAMK2N1 (218309_at)	0.507	0.99	1.00	3.68E-36	1.01E-34
SORBS1 (222513_s_at)	0.508	0.95	0.99	3.39E-36	9.62E-35
OSR2 (213568_at)	0.508	1.00	1.00	2.04E-34	2.63E-33
TMEM99 (226565_at)	0.508	1.00	1.00	5.03E-32	4.01E-31
SUSD2 (227480_at)	0.509	0.99	1.00	6.63E-33	6.08E-32
DIXDC1 (214724_at)	0.509	0.99	1.00	2.23E-35	4.08E-34
CACNA2D1 (227623_at)	0.51	1.00	1.00	1.43E-35	2.83E-34
TPM2 (204083_s_at)	0.51	0.99	1.00	8.64E-35	1.24E-33
C7orf41 (226018_at)	0.51	1.00	1.00	4.02E-37	4.29E-35
TIMP3 (201150_s_at)	0.511	1.00	1.00	5.86E-37	4.29E-35
KIAA1377 (235956_at)	0.511	1.00	1.00	7.82E-36	1.76E-34
BTNL9 (228434_at)	0.511	0.93	1.00	3.1E-33	3.03E-32
SORBS2 (225728_at)	0.512	0.99	1.00	2.93E-35	5.05E-34
GPC6 (227059_at)	0.512	0.96	0.99	3.48E-34	4.23E-33
PDGFD (219304_s_at)	0.513	1.00	1.00	2.81E-32	2.33E-31
TAGLN (205547_s_at)	0.514	1.00	1.00	5.82E-33	5.38E-32
COL21A1 (208096_s_at)	0.514	1.00	1.00	5.1E-35	7.95E-34
HSPB7 (218934_s_at)	0.515	0.63	0.91	2.26E-33	2.27E-32
FBXO32 (225328_at)	0.515	0.99	1.00	1.92E-35	3.62E-34
SLITRK4 (232636_at)	0.515	0.94	0.99	6.03E-32	4.74E-31
PLIN4 (228409_at)	0.515	0.65	0.87	2.3E-25	9.48E-25
KRT15 (204734_at)	0.516	1.00	1.00	1.73E-35	3.31E-34
NTRK2 (221796_at)	0.516	0.99	1.00	2.43E-34	3.06E-33
PRELP (228224_at)	0.516	0.82	0.97	2.23E-35	4.08E-34
NPY1R (205440_s_at)	0.517	0.96	1.00	3.12E-36	9.13E-35
PAK3 (214607_at)	0.517	0.71	0.92	2.72E-36	8.34E-35
DTNA (227084_at)	0.519	0.85	0.98	1.87E-35	3.54E-34
GSTM5 (205752_s_at)	0.519	0.97	1.00	1.16E-34	1.61E-33

KRT31 (206677_at)	0.519	1.00	1.00	5.28E-17	1.39E-16
GATA6 (210002_at)	0.52	0.80	0.93	1.98E-32	1.68E-31
POU6F1 (229809_at)	0.52	0.83	0.98	2.04E-36	6.86E-35
C1orf95 (213925_at)	0.52	0.57	0.81	4.28E-36	1.14E-34
CEACAM7 (206199_at)	0.521	0.78	0.95	1.27E-32	1.12E-31
STXBP6 (220994_s_at)	0.521	0.74	0.98	1.16E-32	1.03E-31
EFCAB4B (228752_at)	0.521	0.99	0.98	1.45E-29	8.52E-29
PLP1 (210198_s_at)	0.522	1.00	1.00	3.82E-33	3.66E-32
CRISPLD1 (223475_at)	0.522	0.94	0.97	4.04E-32	3.26E-31
MYOCD (229339_at)	0.522	0.72	0.92	3.84E-32	3.11E-31
OSR1 (228399_at)	0.522	0.51	0.91	3.01E-34	3.71E-33
PELI2 (219132_at)	0.523	0.93	1.00	1.95E-36	6.65E-35
KIAA1467 (213234_at)	0.523	0.79	0.95	9.83E-34	1.08E-32
DKK1 (204602_at)	0.524	0.81	0.94	4.44E-30	2.77E-29
ABCA9 (239185_at)	0.525	0.91	0.98	6.87E-34	7.78E-33
POSTN (210809_s_at)	0.525	1.00	1.00	2.81E-32	2.33E-31
ZNF573 (217627_at)	0.526	1.00	1.00	2.54E-36	7.99E-35
EFHD1 (209343_at)	0.527	0.89	0.99	2.01E-32	1.7E-31
SPINK1 (206239_s_at)	0.528	0.37	0.91	8.15E-36	1.82E-34
NRXN1 (228547_at)	0.528	0.91	0.99	5.39E-35	8.28E-34
NTM (227566_at)	0.528	1.00	1.00	4.4E-36	1.15E-34
EMX2OS (232531_at)	0.528	0.98	1.00	2.95E-36	8.82E-35
GFRA1 (227550_at)	0.529	0.95	0.99	5.26E-36	1.3E-34
RNF128 (219263_at)	0.529	1.00	1.00	5.17E-35	8.03E-34
HLF (204754_at)	0.53	0.97	1.00	2.2E-35	4.05E-34
PCK1 (208383_s_at)	0.53	0.63	0.84	2.06E-24	8.01E-24
GPC3 (209220_at)	0.531	0.92	1.00	3.44E-33	3.33E-32
LIFR (225575_at)	0.531	0.99	1.00	8.17E-37	4.58E-35
AOC3 (204894_s_at)	0.531	0.98	0.99	2.66E-30	1.7E-29
KCNK5 (219615_s_at)	0.531	0.96	0.98	6.38E-33	5.87E-32
CNTNAP3 (223796_at)	0.531	0.99	1.00	2.12E-33	2.14E-32
COX7A1 (204570_at)	0.532	1.00	1.00	3.72E-34	4.49E-33
PHF17 (218517_at)	0.532	1.00	1.00	1.05E-36	4.96E-35
PCDH7 (228640_at)	0.533	1.00	1.00	3.27E-35	5.5E-34
PRKCB (209685_s_at)	0.533	1.00	1.00	1.25E-33	1.33E-32
PRODH (214203_s_at)	0.533	0.97	0.98	2.06E-35	3.83E-34
SH3BGRL2 (225354_s_at)	0.533	0.97	0.99	2.57E-36	8.05E-35
LYPD6B (228360_at)	0.535	0.98	1.00	5.92E-35	8.97E-34
SYNPO2 (225720_at)	0.535	0.98	1.00	2.24E-34	2.85E-33
CD34 (209543_s_at)	0.536	0.99	1.00	5.71E-36	1.39E-34
DLG2 (228973_at)	0.536	0.64	0.88	1.25E-33	1.33E-32
SYNE1 (209447_at)	0.536	0.94	1.00	6.46E-36	1.53E-34
SYBU (218692_at)	0.536	1.00	1.00	1.39E-35	2.77E-34
KIAA1324 (221874_at)	0.536	0.94	0.95	4.44E-30	2.77E-29
TNXB (208609_s_at)	0.537	0.96	1.00	3.02E-33	2.96E-32
ARHGEF26 (227197_at)	0.537	1.00	1.00	1.31E-36	5.35E-35
DSG2 (217901_at)	0.539	1.00	1.00	1.59E-36	5.87E-35
EFNB2 (202668_at)	0.539	1.00	1.00	1.03E-36	4.92E-35
NOVA1 (205794_s_at)	0.539	0.98	0.99	2.02E-30	1.31E-29

SLC18A2 (205857_at)	0.539	0.97	1.00	3.59E-35	5.97E-34
BCL2 (203685_at)	0.54	1.00	1.00	7.84E-37	4.55E-35
HPGDS (206726_at)	0.54	1.00	1.00	1.82E-31	1.34E-30
FAM117A (221249_s_at)	0.54	1.00	1.00	1.02E-36	4.9E-35
C11orf52 (238805_at)	0.54	0.90	1.00	4.31E-37	4.29E-35
KIAA1211L (228067_at)	0.54	0.86	1.00	1.31E-36	5.35E-35
ID4 (209291_at)	0.541	1.00	1.00	3.97E-37	4.29E-35
SOX8 (226913_s_at)	0.541	0.40	0.66	4.34E-36	1.15E-34
SOX6 (227498_at)	0.541	1.00	1.00	1.17E-33	1.25E-32
PHYH (203335_at)	0.542	1.00	1.00	4E-35	6.53E-34
LINC00312 (220244_at)	0.542	0.91	1.00	8.64E-35	1.24E-33
CHRDL1 (209763_at)	0.542	0.99	0.99	1.64E-28	8.76E-28
FLJ37644 (230419_at)	0.542	0.56	0.86	5.84E-35	8.88E-34
GSN (200696_s_at)	0.543	1.00	1.00	7.11E-37	4.43E-35
LSP1 (203523_at)	0.543	0.79	0.95	1.22E-36	5.24E-35
VIT (227899_at)	0.543	0.97	1.00	3.4E-31	2.43E-30
PDZRN4 (220595_at)	0.543	0.84	0.95	3.04E-28	1.58E-27
ATOH8 (228890_at)	0.544	0.73	0.94	1.61E-36	5.91E-35
AR (226197_at)	0.545	1.00	1.00	9.21E-36	2E-34
LYVE1 (219059_s_at)	0.545	0.91	0.95	8.19E-25	3.27E-24
DBN1 (202806_at)	0.546	0.95	0.99	8.08E-35	1.17E-33
PLA2G16 (209581_at)	0.546	0.87	0.94	3.7E-27	1.75E-26
IL17D (227401_at)	0.546	0.91	0.98	2.04E-31	1.49E-30
LONRF3 (242931_at)	0.546	0.98	1.00	4.08E-33	3.89E-32
PGM5-AS1 (231024_at)	0.546	0.98	1.00	3.63E-36	1E-34
TNS1 (221747_at)	0.547	0.99	1.00	1.63E-35	3.16E-34
SELENBP1 (214433_s_at)	0.547	1.00	1.00	1.62E-34	2.15E-33
C11orf67 (221599_at)	0.547	1.00	1.00	4.43E-37	4.29E-35
TPPP3 (218876_at)	0.547	0.98	1.00	8.86E-25	3.53E-24
CILP (206227_at)	0.548	1.00	1.00	1.54E-21	5.08E-21
C10orf116 (203571_s_at)	0.548	1.00	1.00	2.57E-36	8.05E-35
TMPRSS11E (220431_at)	0.548	0.41	0.76	4.48E-26	1.95E-25
CYP39A1 (220432_s_at)	0.548	1.00	1.00	1.6E-33	1.67E-32
PIK3C2G (215129_at)	0.549	0.96	1.00	3.01E-34	3.71E-33
FCGBP (203240_at)	0.549	0.98	1.00	6.41E-28	3.24E-27
CHPT1 (221675_s_at)	0.549	1.00	1.00	3.5E-37	4.29E-35
ACSS3 (229222_at)	0.549	0.89	0.98	2.56E-34	3.22E-33
PNPLA3 (233030_at)	0.549	0.79	0.96	1.22E-29	7.23E-29
ISM1 (235182_at)	0.549	0.99	1.00	1.01E-35	2.14E-34
BEX5 (229963_at)	0.549	0.80	0.98	1.37E-33	1.44E-32
GPC4 (204984_at)	0.55	0.78	0.90	3.58E-36	9.97E-35
PDK3 (228959_at)	0.55	1.00	1.00	1.35E-33	1.43E-32
PPFIBP1 (214375_at)	0.55	0.87	0.98	1.06E-33	1.16E-32
C1orf115 (218546_at)	0.55	0.99	1.00	1.24E-35	2.55E-34
MUC1 (213693_s_at)	0.551	0.72	0.81	7.4E-25	2.96E-24
PRKAA2 (227892_at)	0.551	0.89	0.98	9.52E-37	4.79E-35
NDNF (219747_at)	0.551	0.83	0.95	1.77E-36	6.25E-35
PAIP2B (221868_at)	0.551	0.66	0.92	5.71E-36	1.39E-34
SEMA3E (206941_x_at)	0.552	0.68	0.84	5.69E-35	8.66E-34

CLIC5 (213317_at)	0.552	0.93	0.97	1.89E-31	1.39E-30
GPR133 (232267_at)	0.552	0.82	0.95	2.09E-32	1.76E-31
AFF3 (227198_at)	0.553	0.96	1.00	2.49E-34	3.14E-33
RPL31 (200962_at)	0.553	0.97	1.00	1.92E-35	3.62E-34
SOBP (218974_at)	0.553	0.99	1.00	5.65E-32	4.47E-31
PDGFC (218718_at)	0.553	1.00	1.00	2.85E-35	4.94E-34
ITGB5 (201124_at)	0.554	1.00	1.00	1.92E-35	3.62E-34
ARHGEF28 (1554003_at)	0.554	0.99	1.00	2.2E-32	1.85E-31
LARP6 (218651_s_at)	0.556	0.91	0.98	8.72E-34	9.65E-33
XKR4 (237802_at)	0.556	0.40	0.76	1.54E-34	2.06E-33
TMEM132B (236824_at)	0.556	0.38	0.67	3.22E-35	5.44E-34
PSAPL1 (1564333_a_at)	0.556	0.98	0.95	9.31E-16	2.33E-15
FAM85A (227917_at)	0.557	1.00	1.00	1.27E-36	5.3E-35
LRP4 (212850_s_at)	0.558	1.00	1.00	2.23E-35	4.08E-34
NPTX2 (213479_at)	0.558	0.89	0.99	5.92E-30	3.63E-29
ABLIM2 (228132_at)	0.558	0.65	0.80	1.26E-36	5.3E-35
TMEM132C (232313_at)	0.558	0.59	0.72	9.62E-35	1.36E-33
PLEKHA6 (229245_at)	0.559	0.44	0.69	1.2E-33	1.28E-32
CBX7 (212914_at)	0.559	1.00	1.00	7.21E-37	4.43E-35
MYEOV (227342_s_at)	0.559	0.40	0.69	1.8E-35	3.42E-34
LONRF1 (226038_at)	0.559	1.00	1.00	5.54E-37	4.29E-35
EEF2K (225545_at)	0.56	1.00	1.00	8.52E-37	4.67E-35
NBEA (221207_s_at)	0.561	0.98	0.99	1.01E-32	8.99E-32
KCNMB4 (222857_s_at)	0.561	0.84	0.99	7.44E-34	8.36E-33
ZNF493 (1558486_at)	0.561	0.98	1.00	1.81E-34	2.37E-33
KANSL1-AS1 (230388_s_at)	0.561	0.94	0.98	5.06E-34	5.9E-33
ACTA1 (203872_at)	0.562	0.15	0.53	2.04E-28	1.08E-27
MATN2 (202350_s_at)	0.562	1.00	1.00	5.31E-37	4.29E-35
TGFBR3 (226625_at)	0.562	1.00	1.00	3.97E-37	4.29E-35
CCDC146 (227091_at)	0.562	0.99	1.00	3.2E-32	2.62E-31
MSRB3 (225782_at)	0.562	1.00	1.00	8.05E-34	9E-33
CST6 (206595_at)	0.563	1.00	1.00	5.33E-22	1.8E-21
MEOX2 (206201_s_at)	0.563	1.00	1.00	6.55E-33	6.01E-32
EPDR1 (223253_at)	0.563	1.00	1.00	1.26E-32	1.11E-31
MAP6 (228943_at)	0.564	0.42	0.66	5.41E-34	6.27E-33
FXYD1 (205384_at)	0.564	0.77	0.93	1.06E-33	1.16E-32
PRIMA1 (230087_at)	0.564	0.99	1.00	2.56E-34	3.22E-33
DPYSL3 (201431_s_at)	0.565	1.00	1.00	8.16E-34	9.1E-33
FMO5 (205776_at)	0.565	0.92	0.98	5.31E-33	4.95E-32
KANK4 (229125_at)	0.565	0.29	0.63	2.14E-33	2.16E-32
COL28A1 (239921_at)	0.565	0.66	0.76	3.98E-34	4.76E-33
PTGIS (208131_s_at)	0.566	0.99	1.00	1.66E-28	8.86E-28
TLE2 (40837_at)	0.566	0.84	1.00	1.31E-36	5.35E-35
TCF7L1 (221016_s_at)	0.566	0.99	1.00	3.64E-32	2.96E-31
GYG2 (215695_s_at)	0.567	0.82	0.90	1.4E-27	6.82E-27
ZNF423 (214761_at)	0.567	0.99	1.00	1.82E-31	1.34E-30
LRFN5 (230644_at)	0.567	0.74	0.91	3.68E-33	3.54E-32
MAOA (212741_at)	0.568	1.00	1.00	1.68E-36	6.07E-35
TNNT1 (213201_s_at)	0.568	0.35	0.63	2.26E-30	1.45E-29

ZNF84 (228630_at)	0.568	1.00	1.00	1.65E-34	2.18E-33
ALDH1A2 (207016_s_at)	0.568	0.66	0.94	1.74E-33	1.79E-32
MARC1 (218865_at)	0.568	0.11	0.43	6.57E-31	4.53E-30
TMEM139 (227753_at)	0.568	0.64	0.69	2.1E-34	2.7E-33
C7orf59 (224890_s_at)	0.568	1.00	1.00	1.87E-36	6.46E-35
MPPED2 (205413_at)	0.569	0.81	0.88	6.25E-35	9.37E-34
FHL1 (201540_at)	0.569	1.00	1.00	1E-35	2.12E-34
SNX1 (213364_s_at)	0.569	1.00	1.00	3.18E-35	5.37E-34
SLC2A13 (227176_at)	0.569	1.00	1.00	3.09E-34	3.8E-33
CFD (205382_s_at)	0.57	1.00	1.00	2.5E-31	1.82E-30
DACT1 (219179_at)	0.57	0.82	0.94	3.81E-31	2.7E-30
NEXN (1552309_a_at)	0.57	0.99	0.99	7.4E-30	4.49E-29
TMEM178A (229302_at)	0.57	0.91	0.99	5.8E-32	4.57E-31
APOD (201525_at)	0.571	1.00	1.00	1.01E-35	2.14E-34
EGF (206254_at)	0.571	0.68	0.83	1.67E-34	2.21E-33
ACOX2 (205364_at)	0.571	0.55	0.65	4.67E-30	2.91E-29
ATP6V1B1 (205473_at)	0.572	0.31	0.81	4.8E-34	5.61E-33
DES (202222_s_at)	0.572	0.47	0.86	4.42E-32	3.55E-31
MAGI1 (225465_at)	0.573	0.95	1.00	4.56E-37	4.29E-35
GDPD2 (220291_at)	0.573	0.68	0.95	1.06E-34	1.48E-33
IRX1 (230472_at)	0.573	0.85	0.94	2.12E-34	2.72E-33
CNGA1 (206417_at)	0.575	0.75	0.93	1.26E-34	1.72E-33
EPHB1 (230425_at)	0.575	0.93	1.00	8.21E-32	6.3E-31
TFAP2B (214451_at)	0.575	1.00	1.00	6.42E-35	9.61E-34
SLCO3A1 (219229_at)	0.575	1.00	1.00	1.96E-34	2.54E-33
MIR100HG (225381_at)	0.575	0.97	0.99	5.51E-32	4.37E-31
INHBB (205258_at)	0.576	0.70	0.95	9.92E-37	4.87E-35
FZD7 (203706_s_at)	0.576	1.00	1.00	8.49E-36	1.87E-34
ADAMTS9-AS2 (1556364_at)	0.576	0.44	0.74	3.35E-34	4.07E-33
PPARG (208510_s_at)	0.577	0.65	0.87	4.36E-28	2.23E-27
DDIT4L (228057_at)	0.577	0.89	0.97	1.54E-31	1.15E-30
CFTR (205043_at)	0.578	0.53	0.76	1.04E-28	5.7E-28
CADPS (239884_at)	0.578	0.57	0.80	3.01E-34	3.71E-33
ZNF652 (225266_at)	0.578	1.00	1.00	3.86E-37	4.29E-35
ZBTB10 (228562_at)	0.578	1.00	1.00	1.96E-34	2.54E-33
LOXL4 (227145_at)	0.578	0.94	1.00	4.04E-32	3.26E-31
OLFML2A (213075_at)	0.578	1.00	1.00	6.28E-37	4.29E-35
MIR181A2HG (232478_at)	0.578	0.32	0.47	1.18E-34	1.63E-33
BBS2 (223227_at)	0.579	1.00	1.00	1.35E-36	5.42E-35
SPRY2 (204011_at)	0.579	0.99	1.00	1.4E-36	5.54E-35
ARHGAP20 (228368_at)	0.579	0.87	0.94	5.63E-30	3.47E-29
EN1 (220559_at)	0.58	1.00	1.00	1.28E-35	2.61E-34
PPP1R14A (227006_at)	0.58	0.88	0.96	3.73E-30	2.34E-29
NEGR1 (243357_at)	0.58	0.99	1.00	7.84E-34	8.78E-33
HSPA12A (214434_at)	0.58	0.95	1.00	5.24E-33	4.89E-32
ALCAM (201952_at)	0.581	1.00	1.00	1.85E-35	3.5E-34
COL8A1 (226237_at)	0.581	0.94	0.98	4.91E-24	1.87E-23
RNF150 (227657_at)	0.581	0.84	0.86	2.17E-32	1.83E-31
C2orf88 (228195_at)	0.581	0.94	0.99	1.76E-34	2.31E-33

CNTN4 (229084_at)	0.581	0.82	0.94	1.92E-30	1.25E-29
GALNTL1 (230418_s_at)	0.582	0.93	0.99	2.44E-27	1.17E-26
PHYHD1 (226846_at)	0.582	0.33	0.59	5.62E-37	4.29E-35
PEG3 (209242_at)	0.583	0.99	1.00	1.09E-33	1.18E-32
DCLK1 (205399_at)	0.583	0.99	1.00	1.07E-29	6.43E-29
CDON (227526_at)	0.583	1.00	1.00	2.12E-33	2.14E-32
LMO3 (204424_s_at)	0.583	0.57	0.79	1.38E-32	1.2E-31
OLFML3 (218162_at)	0.583	1.00	1.00	8.38E-30	5.06E-29
TSPYL5 (213122_at)	0.583	1.00	1.00	4.62E-37	4.29E-35
TSHZ2 (238577_s_at)	0.583	0.94	1.00	1.39E-35	2.77E-34
ARHGAP6 (206167_s_at)	0.584	0.97	1.00	7.56E-33	6.88E-32
MPZ (210280_at)	0.584	0.65	0.74	3.01E-30	1.91E-29
USP34 (212980_at)	0.584	0.93	0.99	2.35E-30	1.51E-29
C10orf10 (209183_s_at)	0.584	0.71	0.90	1.91E-31	1.41E-30
ATP2A3 (213036_x_at)	0.585	0.81	0.90	4.55E-30	2.84E-29
NAP1L2 (219368_at)	0.585	0.99	1.00	7.55E-35	1.1E-33
SDC2 (212158_at)	0.585	1.00	1.00	5.67E-33	5.25E-32
SDPR (222717_at)	0.585	0.99	1.00	1.08E-32	9.56E-32
KANK2 (218418_s_at)	0.585	1.00	1.00	1.26E-35	2.58E-34
MEGF10 (232523_at)	0.585	0.85	0.94	1.9E-28	1.01E-27
LOC100507557 (241745_at)	0.585	0.90	0.99	5.74E-33	5.32E-32
ATL1 (223340_at)	0.586	1.00	1.00	1.57E-36	5.8E-35
C3orf70 (242447_at)	0.586	1.00	1.00	1.88E-33	1.92E-32
LOC100131541 (1558569_at)	0.586	0.87	0.96	3.84E-35	6.33E-34
KLF9 (203543_s_at)	0.587	1.00	1.00	2.27E-34	2.88E-33
CDH12 (207149_at)	0.587	0.44	0.74	1.26E-34	1.72E-33
NAP1L3 (204749_at)	0.587	0.94	0.99	5.84E-30	3.59E-29
ENPP4 (204160_s_at)	0.587	0.99	0.99	9.02E-32	6.89E-31
MAP1B (212233_at)	0.588	1.00	1.00	1.8E-36	6.3E-35
PIK3R1 (212239_at)	0.588	1.00	1.00	9.52E-37	4.79E-35
SNCA (236081_at)	0.588	0.85	0.99	8.64E-35	1.24E-33
ARHGEF10 (216620_s_at)	0.588	1.00	1.00	2.09E-36	6.98E-35
ZBTB20 (222357_at)	0.588	0.82	0.94	1.08E-32	9.56E-32
ZNF415 (205514_at)	0.588	0.88	0.98	2.14E-33	2.16E-32
ACADSB (226030_at)	0.589	1.00	1.00	4.03E-33	3.84E-32
TBC1D24 (227908_at)	0.589	0.81	0.95	2.49E-35	4.43E-34
MYADM (225673_at)	0.589	1.00	1.00	1.49E-35	2.92E-34
LOC100127983 (228107_at)	0.589	0.79	0.99	6.08E-35	9.18E-34
ETV1 (221911_at)	0.59	0.80	0.92	3.29E-30	2.08E-29
ISLR (207191_s_at)	0.59	1.00	1.00	3.97E-29	2.24E-28
FZD4 (218665_at)	0.59	0.97	1.00	5.67E-33	5.25E-32
EBF2 (244885_at)	0.59	0.83	0.94	1.9E-30	1.23E-29
CPED1 (228728_at)	0.59	0.94	0.97	1.27E-32	1.12E-31
LONRF2 (225996_at)	0.59	0.71	0.95	2.78E-34	3.45E-33
FAM171B (227370_at)	0.59	1.00	1.00	4.91E-33	4.61E-32
FAM228B (230435_at)	0.59	0.97	1.00	2.03E-33	2.07E-32
CCND1 (208712_at)	0.591	1.00	1.00	4.56E-37	4.29E-35
SCGB2A2 (206378_at)	0.591	0.96	0.99	2.09E-33	2.11E-32
CUX2 (213920_at)	0.591	0.59	0.71	4E-22	1.36E-21

CTNNBIP1 (203081_at)	0.591	1.00	1.00	6.6E-34	7.5E-33
ZNF559 (224518_s_at)	0.591	0.99	1.00	2.04E-31	1.49E-30
TRAM1L1 (244334_at)	0.591	1.00	1.00	1.36E-34	1.85E-33
SGCE (204688_at)	0.592	1.00	1.00	2.23E-32	1.87E-31
DKK2 (219908_at)	0.592	1.00	1.00	5.27E-21	1.69E-20
EPB41L4B (220161_s_at)	0.592	1.00	1.00	3.12E-36	9.13E-35
LOC286272 (238536_at)	0.592	1.00	1.00	3.77E-34	4.55E-33
GLDN (230360_at)	0.592	1.00	1.00	1.12E-32	9.91E-32
HLA-DQB2 (215536_at)	0.593	1.00	1.00	3.67E-23	1.32E-22
FHL5 (220170_at)	0.593	0.93	0.98	1.1E-25	4.67E-25
RBMS3 (235570_at)	0.593	1.00	1.00	3.4E-33	3.29E-32
CLMP (228082_at)	0.593	0.93	0.97	6.18E-29	3.44E-28
ZC3H6 (227809_at)	0.593	0.94	1.00	1.38E-36	5.52E-35
EBF1 (227646_at)	0.594	1.00	1.00	6.86E-32	5.33E-31
FGF13 (205110_s_at)	0.594	0.99	0.99	1.65E-27	8.01E-27
HSD11B2 (204130_at)	0.594	0.44	0.68	4.04E-32	3.26E-31
CD1A (210325_at)	0.595	1.00	0.99	1.14E-23	4.21E-23
SLC26A2 (224959_at)	0.595	1.00	1.00	3.98E-34	4.76E-33
RORA (226682_at)	0.595	1.00	1.00	1.76E-32	1.51E-31
ZNF204P (214823_at)	0.595	0.93	1.00	4.21E-31	2.97E-30
MFAP5 (209758_s_at)	0.595	1.00	1.00	3.79E-23	1.37E-22
TSPAN2 (227236_at)	0.595	0.93	1.00	1.66E-29	9.71E-29
ZNF540 (238454_at)	0.595	0.65	0.83	2.58E-29	1.48E-28
GRB14 (206204_at)	0.596	0.81	0.90	2.6E-25	1.07E-24
KIT (205051_s_at)	0.596	1.00	1.00	7.63E-37	4.46E-35
SDC4 (202071_at)	0.596	1.00	1.00	2.52E-35	4.46E-34
SAMD4A (212845_at)	0.596	1.00	1.00	5.69E-35	8.66E-34
BOC (225990_at)	0.596	1.00	1.00	5.79E-36	1.4E-34
CA13 (231270_at)	0.596	1.00	1.00	3.35E-34	4.07E-33
DACH1 (228915_at)	0.597	0.92	0.99	7.61E-29	4.19E-28
TSPAN7 (202242_at)	0.597	1.00	1.00	5.32E-35	8.19E-34
FBLN5 (203088_at)	0.597	0.99	0.99	3.27E-31	2.34E-30
KLF8 (230986_at)	0.597	1.00	1.00	6.69E-34	7.59E-33
MYO5C (218966_at)	0.597	1.00	1.00	3.09E-35	5.26E-34
GPAM (225424_at)	0.597	1.00	1.00	1.21E-20	3.8E-20
PRKAG2-AS1 (229156_s_at)	0.597	0.77	0.94	4.4E-35	7.04E-34
SOX10 (209842_at)	0.598	0.93	0.98	3.57E-31	2.54E-30
SPON2 (218638_s_at)	0.598	1.00	1.00	9.61E-30	5.78E-29
MLXIP (225157_at)	0.598	1.00	1.00	8.88E-37	4.75E-35
TENC1 (212494_at)	0.598	0.93	0.99	8.97E-36	1.95E-34
LOC100132046 (1568780_at)	0.598	0.97	0.99	3.14E-33	3.07E-32
MFAP4 (212713_at)	0.599	0.98	0.98	4.75E-24	1.81E-23
PECR (221142_s_at)	0.599	0.67	0.81	1.28E-27	6.29E-27
LOC100128252 (244740_at)	0.599	0.76	0.96	9.36E-35	1.32E-33
FZD8 (227405_s_at)	0.6	0.95	1.00	7.61E-36	1.73E-34
TRIM2 (202342_s_at)	0.6	1.00	1.00	9.2E-34	1.01E-32
ZFPM2 (219778_at)	0.6	0.98	1.00	3.4E-31	2.43E-30
NANOG (220184_at)	0.6	0.62	0.72	6.82E-36	1.59E-34
ZNF704 (223366_at)	0.6	1.00	1.00	7.86E-35	1.14E-33

PDLIM3 (209621_s_at)	0.601	0.33	0.52	1.51E-32	1.31E-31
MAP1A (203151_at)	0.602	0.97	1.00	2.84E-31	2.05E-30
TNNT2 (215389_s_at)	0.602	0.65	0.88	2.26E-35	4.11E-34
MAGI2 (209737_at)	0.602	1.00	1.00	8.08E-35	1.17E-33
PLCB1 (213222_at)	0.602	1.00	1.00	4.6E-32	3.68E-31
LPHN3 (236264_at)	0.602	0.84	0.96	6.26E-29	3.47E-28
CNRIP1 (226751_at)	0.602	0.99	1.00	2.89E-32	2.39E-31
CYBRD1 (222453_at)	0.602	1.00	1.00	1.01E-35	2.14E-34
SCUBE3 (228407_at)	0.602	0.68	0.83	1.87E-30	1.22E-29
LOC100506098 (227985_at)	0.602	0.73	0.89	1.62E-31	1.2E-30
CFL2 (224352_s_at)	0.603	1.00	1.00	9.69E-33	8.67E-32
PCM1 (228905_at)	0.603	1.00	1.00	1.04E-35	2.19E-34
FLJ13197 (219871_at)	0.603	0.78	0.95	2.6E-34	3.25E-33
FAM150B (238018_at)	0.603	0.95	0.96	1.4E-23	5.16E-23
FAM221A (228600_x_at)	0.603	0.98	0.99	7.17E-33	6.56E-32
WFS1 (202908_at)	0.604	1.00	1.00	5.62E-37	4.29E-35
FERMT2 (209210_s_at)	0.604	1.00	1.00	7.31E-32	5.66E-31
ASPN (219087_at)	0.604	1.00	1.00	5.63E-30	3.47E-29
EDA (206217_at)	0.605	0.57	0.82	2.21E-36	7.28E-35
PER1 (36829_at)	0.605	0.95	1.00	6.2E-36	1.48E-34
SVEP1 (213247_at)	0.605	0.97	0.99	3.53E-27	1.67E-26
TTC18 (229169_at)	0.605	0.64	0.82	2.54E-33	2.53E-32
MAT1A (205813_s_at)	0.606	0.02	0.31	2.47E-32	2.06E-31
TCEAL3 (227279_at)	0.606	1.00	1.00	4.19E-37	4.29E-35
DCD (1553946_at)	0.606	0.96	0.97	2.14E-33	2.16E-32
CCBE1 (229641_at)	0.606	0.93	1.00	1.56E-28	8.38E-28
ZNF677 (1569039_s_at)	0.606	0.87	0.99	6.17E-35	9.28E-34
CADM3 (221921_s_at)	0.607	0.57	0.73	9.75E-35	1.37E-33
VKORC1L1 (224881_at)	0.607	1.00	1.00	6.69E-35	9.94E-34
C1orf132 (228528_at)	0.607	1.00	1.00	1.09E-29	6.5E-29
TMPRSS2 (226553_at)	0.608	0.55	0.64	6.68E-32	5.2E-31
FNBP1L (215017_s_at)	0.608	1.00	1.00	1.42E-36	5.56E-35
AK7 (1553734_at)	0.608	0.66	0.89	6.74E-31	4.63E-30
USP54 (227334_at)	0.608	1.00	1.00	1.49E-35	2.92E-34
RUNX1T1 (228827_at)	0.609	1.00	1.00	1.54E-34	2.06E-33
DPT (213071_at)	0.609	1.00	1.00	4.47E-28	2.29E-27
HSPA2 (211538_s_at)	0.609	1.00	1.00	8.73E-33	7.85E-32
LAMC2 (202267_at)	0.609	0.98	1.00	4.11E-36	1.1E-34
MRVI1 (226047_at)	0.609	0.94	0.99	3.17E-30	2.01E-29
GPD1L (212510_at)	0.609	1.00	1.00	4.65E-36	1.2E-34
SERHL2 (217276_x_at)	0.609	0.63	0.81	4.9E-32	3.91E-31
LOC285419 (1563462_at)	0.609	0.57	0.78	8.1E-32	6.23E-31
RBPMS2 (228802_at)	0.609	0.68	0.82	1.02E-31	7.76E-31
PIP5K1B (205632_s_at)	0.61	0.83	0.93	5.56E-30	3.43E-29
SCEL (206884_s_at)	0.61	1.00	1.00	4.69E-28	2.4E-27
AKAP12 (227530_at)	0.61	1.00	1.00	4.14E-33	3.93E-32
MERTK (206028_s_at)	0.61	0.31	0.56	8.98E-32	6.86E-31
PLXNA4 (232317_at)	0.61	0.86	0.97	9.6E-36	2.06E-34
ADRA2A (209869_at)	0.611	1.00	1.00	1.05E-27	5.18E-27

MCC (226225_at)	0.611	1.00	1.00	1.48E-36	5.69E-35
TLE1 (203221_at)	0.611	1.00	1.00	7.71E-36	1.75E-34
WISP2 (205792_at)	0.611	0.98	1.00	2.6E-25	1.07E-24
KIAA0895 (213424_at)	0.611	0.95	1.00	3.3E-34	4.03E-33
FAM149A (214890_s_at)	0.611	0.54	0.82	1.06E-33	1.16E-32
CLSTN2 (219414_at)	0.611	0.56	0.82	4.36E-33	4.13E-32
C14orf28 (235369_at)	0.611	0.99	1.00	6.78E-34	7.68E-33
STK32A (229866_at)	0.611	0.77	0.88	7.31E-32	5.66E-31
RNF180 (242033_at)	0.611	0.95	0.99	1.79E-31	1.33E-30
AGR2 (209173_at)	0.612	0.61	0.76	2.68E-23	9.71E-23
CREBRF (235556_at)	0.612	1.00	1.00	3.87E-34	4.65E-33
LOC157562 (213776_at)	0.612	0.71	0.92	4.9E-35	7.69E-34
TRIM73 (1554250_s_at)	0.612	0.99	1.00	4.8E-34	5.61E-33
SRGAP2B (242471_at)	0.612	1.00	1.00	6.18E-29	3.44E-28
NTN1 (227816_at)	0.613	0.91	0.98	4.73E-34	5.55E-33
MCF2L (212935_at)	0.614	0.70	0.96	2.95E-36	8.82E-35
SRGAP2 (213329_at)	0.614	0.86	1.00	6.09E-34	6.98E-33
LRRN4CL (1556427_s_at)	0.614	1.00	1.00	5.8E-24	2.19E-23
LPAR1 (204037_at)	0.615	1.00	1.00	4.66E-33	4.39E-32
ENO2 (201313_at)	0.615	0.69	0.85	4.55E-34	5.35E-33
SMURF2 (227489_at)	0.615	1.00	1.00	6.73E-37	4.35E-35
ACVR1C (1552519_at)	0.615	0.75	0.89	1.35E-28	7.28E-28
FGD5 (226985_at)	0.615	0.98	1.00	6.34E-34	7.25E-33
RHOB (212099_at)	0.616	1.00	1.00	7.63E-37	4.46E-35
ANKRD36B (220940_at)	0.616	1.00	1.00	2.32E-33	2.32E-32
WDFY3-AS2 (238081_at)	0.616	0.95	1.00	1.47E-32	1.28E-31
CAT (201432_at)	0.617	1.00	1.00	5.78E-37	4.29E-35
ECM2 (206101_at)	0.617	1.00	1.00	9.19E-28	4.57E-27
HSPB2 (205824_at)	0.617	0.64	0.90	1.15E-33	1.24E-32
TBX15 (230438_at)	0.617	0.93	0.98	1.33E-29	7.85E-29
TGFB1I1 (209651_at)	0.617	1.00	1.00	8.42E-32	6.45E-31
GKAP1 (234192_s_at)	0.617	0.94	0.98	5.53E-29	3.09E-28
CYSTM1 (224707_at)	0.617	1.00	1.00	6.87E-35	1.02E-33
SLAIN1 (225619_at)	0.617	0.87	0.96	2.93E-35	5.05E-34
LINC00667 (228160_at)	0.617	0.99	1.00	1.52E-33	1.59E-32
APBA1 (228101_at)	0.618	0.70	0.90	2.03E-33	2.07E-32
GPR64 (206002_at)	0.618	0.31	0.70	1.02E-29	6.13E-29
ABCA8 (204719_at)	0.619	1.00	1.00	6.41E-29	3.55E-28
PEG10 (212094_at)	0.619	0.92	0.95	5.88E-29	3.28E-28
TCEAL2 (211276_at)	0.619	0.57	0.87	4.54E-33	4.29E-32
CYP1B1 (202437_s_at)	0.62	1.00	1.00	2.49E-25	1.02E-24
AQPEP (235382_at)	0.62	0.85	0.95	3.61E-27	1.71E-26
MXRA7 (212509_s_at)	0.62	1.00	1.00	3.83E-36	1.04E-34
LOC100170939 (214850_at)	0.62	0.99	1.00	1.63E-35	3.16E-34
RELN (205923_at)	0.621	0.95	0.98	7.18E-31	4.91E-30
PTCH1 (209815_at)	0.621	1.00	1.00	1.4E-33	1.48E-32
SYT17 (205613_at)	0.621	0.51	0.78	2.15E-34	2.75E-33
ACAT1 (205412_at)	0.622	1.00	1.00	6.55E-37	4.29E-35
GFRA3 (229936_at)	0.622	0.44	0.77	4.44E-29	2.5E-28

DAAM2 (212793_at)	0.622	0.96	1.00	9.7E-34	1.06E-32
SLC22A15 (228497_at)	0.622	0.95	1.00	3.2E-32	2.62E-31
CTGF (209101_at)	0.624	1.00	1.00	9.5E-27	4.34E-26
SMAD9 (227719_at)	0.624	1.00	1.00	8.87E-32	6.79E-31
SLC12A2 (225835_at)	0.624	1.00	1.00	3.62E-31	2.57E-30
ITGA8 (235666_at)	0.624	0.96	0.98	1.87E-23	6.83E-23
ABLIM3 (205730_s_at)	0.624	0.80	0.94	1.7E-32	1.46E-31
DGAT2 (224327_s_at)	0.624	1.00	1.00	2.99E-26	1.32E-25
MR1 (235352_at)	0.625	0.94	1.00	7.13E-32	5.52E-31
ATP8A1 (213106_at)	0.625	0.98	1.00	9.83E-34	1.08E-32
KCNMA1 (221584_s_at)	0.626	0.87	0.94	1.07E-29	6.43E-29
CYP4F8 (210576_at)	0.626	0.55	0.59	2.18E-14	5.11E-14
MRAS (225185_at)	0.626	0.96	0.99	1.45E-26	6.54E-26
UACA (238868_at)	0.626	0.96	1.00	5.92E-35	8.97E-34
MCOLN3 (229797_at)	0.626	1.00	1.00	8.29E-29	4.56E-28
ADCK3 (218168_s_at)	0.626	1.00	1.00	1.87E-36	6.46E-35
ASPA (228807_at)	0.627	0.61	0.63	7.27E-31	4.97E-30
GRIA2 (205358_at)	0.627	0.35	0.63	4.17E-29	2.35E-28
PSD3 (203355_s_at)	0.627	1.00	1.00	1.26E-32	1.11E-31
NTN4 (223315_at)	0.627	1.00	1.00	2.79E-28	1.46E-27
KLHDC1 (1552733_at)	0.627	0.99	1.00	2.37E-29	1.37E-28
CSRP1 (200621_at)	0.628	1.00	1.00	4.99E-34	5.84E-33
GPR12 (1559603_at)	0.628	0.43	0.78	1.57E-32	1.35E-31
MB (204179_at)	0.628	0.84	0.94	1.28E-29	7.58E-29
LAMC3 (219407_s_at)	0.628	0.69	0.94	9.44E-33	8.46E-32
DIP2C (212504_at)	0.628	0.94	1.00	4.77E-35	7.53E-34
SCCPDH (201825_s_at)	0.628	0.98	1.00	6.01E-31	4.15E-30
DZIP1L (239785_at)	0.628	0.76	0.91	7.65E-35	1.11E-33
LOC654342 (242133_s_at)	0.628	0.95	0.98	3.01E-34	3.71E-33
SHANK2 (213308_at)	0.629	0.70	0.90	8.92E-29	4.89E-28
FRMD4A (225163_at)	0.629	1.00	1.00	1.3E-35	2.63E-34
STARD9 (227108_at)	0.629	0.86	0.98	9.12E-35	1.29E-33
PALM (203859_s_at)	0.63	0.49	0.80	1.35E-35	2.71E-34
INMT (224061_at)	0.63	0.82	0.95	1.86E-31	1.37E-30
FILIP1 (1556325_at)	0.63	0.87	0.93	4.21E-27	1.98E-26
PCGF5 (226326_at)	0.63	1.00	1.00	1.87E-36	6.46E-35
UBE2QL1 (226612_at)	0.63	0.97	0.98	2.82E-18	7.89E-18
AHNAK2 (212992_at)	0.631	1.00	1.00	4E-36	1.08E-34
CDKN1C (213348_at)	0.632	1.00	1.00	1.71E-33	1.77E-32
MAL (204777_s_at)	0.632	1.00	1.00	2.8E-30	1.78E-29
PPP2R2B (213849_s_at)	0.632	1.00	1.00	7.08E-33	6.48E-32
NPHP3 (235410_at)	0.632	1.00	1.00	2.13E-26	9.52E-26
BCHE (205433_at)	0.633	1.00	1.00	1.88E-24	7.34E-24
CYP3A5 (205765_at)	0.633	0.99	1.00	2.59E-27	1.24E-26
FBP1 (209696_at)	0.633	0.76	0.92	3.08E-24	1.19E-23
SYNE2 (202761_s_at)	0.633	1.00	1.00	5.32E-35	8.19E-34
OSBPL1A (209485_s_at)	0.633	1.00	1.00	1.36E-36	5.47E-35
SVIP (226278_at)	0.633	1.00	1.00	1.94E-30	1.26E-29
CA3 (204865_at)	0.634	0.59	0.76	4.38E-31	3.08E-30

CPE (201117_s_at)	0.634	1.00	1.00	3.29E-30	2.08E-29
INPP5A (203006_at)	0.634	1.00	1.00	2.44E-36	7.77E-35
CNTN3 (229831_at)	0.634	0.98	1.00	1.27E-17	3.43E-17
GABARAPL1 (208869_s_at)	0.634	1.00	1.00	1.9E-36	6.5E-35
C19orf42 (221988_at)	0.634	0.97	1.00	4.2E-34	4.97E-33
CAB39L (225914_s_at)	0.634	1.00	1.00	4.4E-35	7.04E-34
ESPN (223549_s_at)	0.634	0.64	0.74	1.34E-32	1.17E-31
PNPLA7 (228383_at)	0.634	0.20	0.53	7.86E-33	7.13E-32
PGR (228554_at)	0.635	0.88	0.94	8.21E-32	6.3E-31
PPOX (204788_s_at)	0.635	1.00	1.00	9.09E-36	1.97E-34
WNT2 (205648_at)	0.635	0.17	0.72	2.85E-29	1.63E-28
TMEM55A (226338_at)	0.635	1.00	1.00	5.56E-30	3.43E-29
ADAM33 (232570_s_at)	0.635	0.81	0.93	4.16E-31	2.94E-30
FAM201A (1557014_a_at)	0.635	0.23	0.66	3.4E-31	2.43E-30
TAC1 (206552_s_at)	0.636	0.62	0.88	6.44E-27	2.98E-26
ZNF177 (207417_s_at)	0.636	0.83	0.95	1.74E-30	1.14E-29
NUAK1 (204589_at)	0.636	1.00	1.00	6.69E-34	7.59E-33
ZNF273 (243661_at)	0.636	0.92	0.97	3.16E-32	2.6E-31
PDZD2 (209493_at)	0.636	1.00	1.00	3.24E-32	2.66E-31
LOC100289187 (230277_at)	0.636	0.55	0.73	2.33E-35	4.19E-34
MYLK (224823_at)	0.637	1.00	1.00	8.61E-34	9.55E-33
SLIT3 (203812_at)	0.637	0.81	0.91	2.14E-28	1.13E-27
PPM1L (228108_at)	0.637	1.00	1.00	2.3E-35	4.14E-34
ZNF320 (229614_at)	0.637	0.99	1.00	5.99E-30	3.68E-29
PLXNA2 (213030_s_at)	0.638	1.00	1.00	3.59E-35	5.97E-34
SH3BGR (204979_s_at)	0.638	0.71	0.86	1.9E-35	3.59E-34
TPM1 (206117_at)	0.638	0.82	0.97	2.81E-32	2.33E-31
YBX2 (219704_at)	0.638	0.60	0.64	7.08E-27	3.26E-26
LOC202181 (220609_at)	0.638	0.87	0.96	1.01E-32	8.99E-32
ALDOC (202022_at)	0.639	0.99	1.00	1.74E-32	1.5E-31
ITGA9 (227297_at)	0.639	1.00	1.00	3.35E-34	4.07E-33
TTC28 (213058_at)	0.639	1.00	1.00	6.96E-34	7.87E-33
SEPT11 (201307_at)	0.639	1.00	1.00	2.02E-29	1.17E-28
PLD6 (227037_at)	0.639	0.99	1.00	8.26E-36	1.84E-34
SDCBP2-AS1 (236520_at)	0.639	0.92	0.98	4.9E-35	7.69E-34
CRIP2 (208978_at)	0.64	0.66	0.92	3.35E-34	4.07E-33
EPHX1 (202017_at)	0.64	0.99	1.00	6.17E-35	9.28E-34
MFI2 (235911_at)	0.64	0.96	0.97	4.35E-24	1.66E-23
NCAM1 (227394_at)	0.64	0.90	0.99	2.12E-34	2.72E-33
PRKAR2B (203680_at)	0.64	1.00	1.00	8.25E-27	3.78E-26
SORL1 (212560_at)	0.64	1.00	1.00	1.2E-27	5.88E-27
TCEA3 (226388_at)	0.64	1.00	1.00	1.17E-36	5.15E-35
TGFBI (201506_at)	0.64	1.00	1.00	8.61E-36	1.9E-34
CREB5 (229228_at)	0.64	0.98	1.00	2.06E-28	1.09E-27
P4HTM (222125_s_at)	0.64	1.00	1.00	9.85E-30	5.92E-29
PARVA (213675_at)	0.64	1.00	1.00	5.33E-36	1.31E-34
TRAPPC6A (204985_s_at)	0.64	0.47	0.68	1E-35	2.12E-34
N4BP2L1 (243843_at)	0.64	0.80	0.94	3.76E-31	2.67E-30
NOSTRIN (226992_at)	0.64	0.86	0.94	9.95E-29	5.44E-28

HOTAIR (239153_at)	0.64	0.93	0.99	5.44E-22	1.83E-21
HOXB3 (228904_at)	0.641	0.99	1.00	2.89E-32	2.39E-31
IRS1 (204686_at)	0.641	1.00	1.00	1.52E-36	5.75E-35
CRYZL1 (1552347_at)	0.642	1.00	1.00	9.88E-35	1.39E-33
PLEKHG5 (227142_at)	0.642	0.82	0.98	9.7E-34	1.06E-32
CAMSAP3 (1568617_a_at)	0.642	0.89	0.99	2.03E-33	2.07E-32
ADSSL1 (226325_at)	0.642	0.73	0.95	5.67E-33	5.25E-32
LOC400043 (226582_at)	0.642	0.99	1.00	1.22E-28	6.63E-28
LHX2 (206140_at)	0.643	0.85	0.90	8.24E-15	1.98E-14
MYOT (219728_at)	0.643	0.77	0.91	5.59E-33	5.19E-32
ZNF83 (236429_at)	0.643	0.98	1.00	1.62E-33	1.69E-32
TMEM108 (223524_s_at)	0.643	0.65	0.87	3.4E-33	3.29E-32
GAL3ST4 (219815_at)	0.643	0.93	0.98	5.86E-37	4.29E-35
SGSM1 (230287_at)	0.643	0.62	0.75	3.22E-35	5.44E-34
PKP2 (207717_s_at)	0.644	0.95	0.99	9.96E-27	4.54E-26
MYRIP (214156_at)	0.644	0.95	1.00	1.44E-25	6.03E-25
PPP1R3B (222662_at)	0.644	1.00	1.00	5.33E-34	6.2E-33
IGSF10 (230670_at)	0.644	0.96	0.99	9.2E-20	2.75E-19
FREM2 (230964_at)	0.644	0.81	0.94	1.5E-28	8.09E-28
DYNC1I1 (205348_s_at)	0.645	0.80	0.92	1.47E-28	7.91E-28
SLC27A2 (205769_at)	0.645	0.57	0.78	3.47E-26	1.52E-25
CMYA5 (233520_s_at)	0.645	0.77	0.92	8.04E-31	5.47E-30
DDR2 (225442_at)	0.646	1.00	1.00	6.57E-26	2.82E-25
IL17RD (227997_at)	0.646	1.00	1.00	3.1E-33	3.03E-32
ABHD6 (45288_at)	0.646	1.00	1.00	1.74E-34	2.28E-33
MLPH (218211_s_at)	0.646	1.00	1.00	5.16E-31	3.59E-30
C17orf108 (213195_at)	0.646	0.77	0.92	9.92E-37	4.87E-35
SMAD4 (235725_at)	0.647	1.00	1.00	7.31E-37	4.43E-35
PFN2 (204992_s_at)	0.647	1.00	1.00	3.27E-35	5.5E-34
CDK14 (204604_at)	0.647	0.99	1.00	7.76E-33	7.05E-32
TMEM63A (228549_at)	0.647	0.97	1.00	1.05E-33	1.14E-32
HEPH (203903_s_at)	0.647	0.98	0.99	3.64E-28	1.88E-27
G0S2 (213524_s_at)	0.647	1.00	0.97	6.55E-13	1.45E-12
LRRN1 (226884_at)	0.647	0.82	0.94	1.29E-31	9.68E-31
WDSUB1 (226668_at)	0.647	1.00	1.00	2.12E-34	2.72E-33
FGF1 (205117_at)	0.648	0.64	0.71	2.35E-30	1.51E-29
PCDH9 (219737_s_at)	0.648	0.66	0.88	2.81E-34	3.5E-33
C19orf18 (236847_at)	0.648	0.62	0.66	3.64E-32	2.96E-31
EFNA5 (214036_at)	0.649	1.00	1.00	6.91E-36	1.6E-34
NPY5R (207400_at)	0.649	0.54	0.83	2.26E-32	1.89E-31
PPAP2B (212230_at)	0.649	1.00	1.00	2.19E-28	1.16E-27
PIEZO2 (219602_s_at)	0.649	0.81	0.92	4.8E-27	2.24E-26
GPR98 (223582_at)	0.649	0.35	0.63	8.45E-28	4.22E-27
ANTXR2 (225524_at)	0.649	1.00	1.00	2.47E-28	1.3E-27
GCNT2 (230788_at)	0.65	0.79	0.96	2.58E-33	2.56E-32
PDE9A (205593_s_at)	0.65	0.55	0.80	1.06E-30	7.12E-30
SPTAN1 (215235_at)	0.65	1.00	1.00	5.69E-28	2.88E-27
HIBADH (224812_at)	0.65	1.00	1.00	3.73E-36	1.02E-34
ZNF608 (229817_at)	0.65	0.91	0.95	2.23E-33	2.24E-32

MYCT1 (231947_at)	0.65	0.99	1.00	4.55E-31	3.19E-30
FAM13C (1554547_at)	0.65	1.00	1.00	1.06E-29	6.35E-29
SLCO4C1 (222071_s_at)	0.65	0.60	0.78	2.95E-20	9.04E-20
HRCT1 (235496_at)	0.65	0.22	0.56	2.03E-32	1.72E-31
NRTN (210683_at)	0.651	0.36	0.69	3.45E-19	1E-18
EFEMP2 (206580_s_at)	0.651	1.00	1.00	3.04E-28	1.58E-27
TCEAL7 (227705_at)	0.651	0.91	0.95	3.39E-26	1.49E-25
IL31RA (243541_at)	0.651	0.69	0.95	2.83E-28	1.47E-27
HOXC9 (231936_at)	0.652	0.87	0.99	2.74E-34	3.41E-33
UCHL1 (201387_s_at)	0.652	0.73	0.87	5.19E-24	1.97E-23
PDE5A (227088_at)	0.652	0.99	1.00	2.39E-28	1.25E-27
RALGPS1 (204199_at)	0.652	0.94	0.99	8.35E-31	5.67E-30
KIF21A (226003_at)	0.652	1.00	1.00	5.1E-32	4.06E-31
SPAG16 (219109_at)	0.653	1.00	1.00	2.15E-36	7.13E-35
FHOD3 (218980_at)	0.653	0.77	0.94	1.55E-30	1.02E-29
ZKSCAN3 (235840_at)	0.653	0.62	0.80	1.16E-34	1.61E-33
DYNLRB2 (238116_at)	0.653	0.69	0.87	6.77E-32	5.26E-31
USP30 (227572_at)	0.653	1.00	1.00	3.84E-35	6.33E-34
FLNC (207876_s_at)	0.654	1.00	1.00	3.94E-32	3.19E-31
PHF15 (212660_at)	0.654	0.99	1.00	4.97E-35	7.77E-34
ZDHHC2 (222731_at)	0.654	1.00	1.00	2.88E-27	1.38E-26
HS6ST2 (1552767_a_at)	0.654	0.68	0.85	5.23E-32	4.16E-31
ZNF862 (226808_at)	0.654	0.97	0.99	4.55E-29	2.56E-28
LOC100505783 (1558906_a_at)	0.654	0.73	0.87	4.91E-36	1.24E-34
FGFR1 (226705_at)	0.655	0.98	0.99	2.27E-27	1.09E-26
LOC440993 (1557293_at)	0.655	1.00	1.00	4.08E-33	3.89E-32
MAGI2-AS3 (227554_at)	0.655	1.00	1.00	4.11E-24	1.57E-23
ADRBK2 (228771_at)	0.656	0.99	1.00	3.64E-28	1.88E-27
MTR (226969_at)	0.656	0.94	1.00	5.46E-27	2.54E-26
ROR1 (205805_s_at)	0.656	0.97	1.00	3.81E-31	2.7E-30
SGCB (226112_at)	0.656	1.00	1.00	2.01E-36	6.79E-35
SOX13 (38918_at)	0.656	0.92	0.96	2.4E-34	3.02E-33
AS3MT (223652_at)	0.656	0.97	0.99	1.54E-36	5.78E-35
WLS (221958_s_at)	0.656	1.00	1.00	1.08E-36	4.97E-35
ZNF766 (227284_at)	0.656	1.00	1.00	1.61E-36	5.91E-35
GPR146 (228770_at)	0.656	0.53	0.85	5.39E-35	8.28E-34
FLG2 (1569410_at)	0.656	1.00	1.00	7.96E-27	3.65E-26
LOC730102 (241607_at)	0.656	0.96	1.00	3.45E-35	5.78E-34
TNNC1 (209904_at)	0.657	0.02	0.19	1.66E-31	1.23E-30
CAND2 (213547_at)	0.657	0.78	0.91	6.09E-34	6.98E-33
KLF15 (231015_at)	0.657	0.57	0.63	1.4E-34	1.9E-33
CLMN (213839_at)	0.657	0.90	0.95	1.34E-31	1E-30
CERS6 (212446_s_at)	0.657	1.00	1.00	4.22E-36	1.12E-34
GUSBP1 (1556742_at)	0.657	0.64	0.70	8.25E-31	5.6E-30
CDR2 (209501_at)	0.658	0.72	0.94	2.53E-27	1.21E-26
FGF2 (204422_s_at)	0.658	0.93	0.98	2.95E-31	2.13E-30
STON1 (213413_at)	0.658	0.97	1.00	4.17E-29	2.35E-28
BEX4 (215440_s_at)	0.658	1.00	1.00	2.59E-35	4.56E-34
ZMAT1 (226344_at)	0.658	0.89	0.97	5.65E-26	2.44E-25

LINC00663 (1555363_s_at)	0.658	0.60	0.62	1.48E-29	8.73E-29
EFHA2 (238458_at)	0.658	0.93	0.97	4.59E-24	1.75E-23
CKMT2 (205295_at)	0.659	0.76	0.88	7.07E-29	3.91E-28
DNM1 (215116_s_at)	0.659	0.86	0.98	1.12E-24	4.44E-24
GHR (205498_at)	0.659	1.00	1.00	5.36E-30	3.31E-29
MGP (202291_s_at)	0.659	1.00	1.00	1.53E-30	1.01E-29
MYH3 (205940_at)	0.659	0.20	0.50	4.9E-32	3.91E-31
ZNF137P (207394_at)	0.659	0.86	0.96	1.42E-30	9.4E-30
STK32B (219686_at)	0.659	0.62	0.66	7.03E-32	5.47E-31
TRIM45 (242056_at)	0.659	0.99	1.00	5.78E-34	6.66E-33
LOC100049716 (228647_at)	0.659	1.00	1.00	4.11E-28	2.11E-27
LOC100130429 (241851_x_at)	0.659	0.81	0.89	1.46E-33	1.53E-32
RFX3 (230403_at)	0.66	0.96	1.00	8.18E-30	4.94E-29
XIST (224588_at)	0.66	0.45	0.65	1.76E-33	1.81E-32
RABGAP1L (203020_at)	0.66	1.00	1.00	7.21E-37	4.43E-35
MARC2 (221636_s_at)	0.66	0.98	1.00	2.32E-31	1.69E-30
GPRASP2 (228027_at)	0.66	0.94	1.00	2.27E-36	7.46E-35
AGAP11 (1557380_at)	0.66	0.71	0.84	1.7E-31	1.26E-30
GIMAP8 (235306_at)	0.66	1.00	1.00	3.33E-30	2.1E-29
SBSPON (235210_s_at)	0.66	0.94	0.99	1.3E-31	9.79E-31
KIAA1324L (235301_at)	0.66	0.97	1.00	2.23E-31	1.63E-30
NPTX1 (204684_at)	0.661	0.46	0.84	1.32E-30	8.74E-30
ABCA6 (217504_at)	0.661	0.86	0.97	9.73E-27	4.44E-26
SLC47A1 (219525_at)	0.661	1.00	1.00	2.85E-29	1.63E-28
RBP7 (238066_at)	0.661	1.00	1.00	4.26E-32	3.42E-31
FAT3 (236029_at)	0.661	0.64	0.83	1.25E-28	6.79E-28
HSPB6 (226304_at)	0.661	0.63	0.72	7.22E-30	4.39E-29
ABAT (209459_s_at)	0.662	1.00	1.00	2.4E-25	9.9E-25
TRPC1 (205802_at)	0.662	0.98	1.00	5.36E-30	3.31E-29
SERF2 (226692_at)	0.662	1.00	1.00	1.18E-34	1.63E-33
ABI3BP (223395_at)	0.662	1.00	1.00	8.08E-26	3.45E-25
IL20RA (219115_s_at)	0.662	1.00	1.00	8.73E-24	3.26E-23
CYP26B1 (219825_at)	0.662	1.00	1.00	3.29E-32	2.69E-31
VWA5A (205011_at)	0.663	1.00	1.00	3.54E-35	5.91E-34
ASMTL (36553_at)	0.663	1.00	1.00	7.95E-34	8.89E-33
SIK2 (223430_at)	0.663	1.00	1.00	1.82E-35	3.46E-34
EIF4E3 (225941_at)	0.663	1.00	1.00	2.15E-34	2.75E-33
ZNF506 (221626_at)	0.663	1.00	1.00	7.25E-35	1.06E-33
WIPF3 (229849_at)	0.663	0.62	0.66	1.33E-29	7.85E-29
LOC100130097 (241376_at)	0.663	0.43	0.67	1.1E-28	5.98E-28
HES1 (203394_s_at)	0.664	1.00	1.00	8.49E-36	1.87E-34
URI1 (222266_at)	0.664	1.00	1.00	8.87E-35	1.27E-33
OXGR1 (1553319_at)	0.664	0.52	0.80	8.25E-31	5.6E-30
FAM3B (227194_at)	0.664	0.73	0.86	1.17E-28	6.33E-28
COLEC12 (221019_s_at)	0.664	1.00	1.00	6.61E-25	2.65E-24
LOC202781 (235587_at)	0.664	1.00	1.00	2.96E-32	2.45E-31
FBXL13 (1556770_a_at)	0.664	0.55	0.78	2.75E-33	2.72E-32
CKB (200884_at)	0.665	1.00	1.00	7.4E-36	1.69E-34
CPM (235019_at)	0.665	1.00	1.00	6.32E-31	4.36E-30

LIF (205266_at)	0.665	0.34	0.76	1.04E-28	5.7E-28
PDZK1 (205380_at)	0.665	0.64	0.79	3.38E-19	9.85E-19
TACC2 (202289_s_at)	0.665	1.00	1.00	9.65E-37	4.82E-35
PID1 (219093_at)	0.665	1.00	1.00	7.68E-28	3.85E-27
ST6GAL2 (228821_at)	0.665	0.57	0.88	5.7E-30	3.51E-29
ZNF790-AS1 (235779_at)	0.665	0.72	0.92	4.52E-35	7.19E-34
KIAA2022 (244370_at)	0.665	0.86	0.99	3.48E-31	2.49E-30
C18orf1 (207996_s_at)	0.666	1.00	1.00	3.33E-24	1.28E-23
EZH1 (32259_at)	0.666	1.00	1.00	7.13E-32	5.52E-31
GLI2 (228537_at)	0.666	0.78	0.96	1.09E-35	2.28E-34
EDAR (220048_at)	0.666	0.71	0.89	1.3E-30	8.64E-30
PPP1R9A (228494_at)	0.666	0.82	0.91	1.14E-25	4.82E-25
JAZF1 (225798_at)	0.666	1.00	1.00	8.84E-33	7.95E-32
