# Supplementary figures and images for: Cellular dissection of psoriasis for transcriptome analyses and the post-GWAS era
Source: BMC Med Genomics. 2014 May 22;7:27. doi: 10.1186/1755-8794-7-27 (PMC4060870; doi:10.1186/1755-8794-7-27)

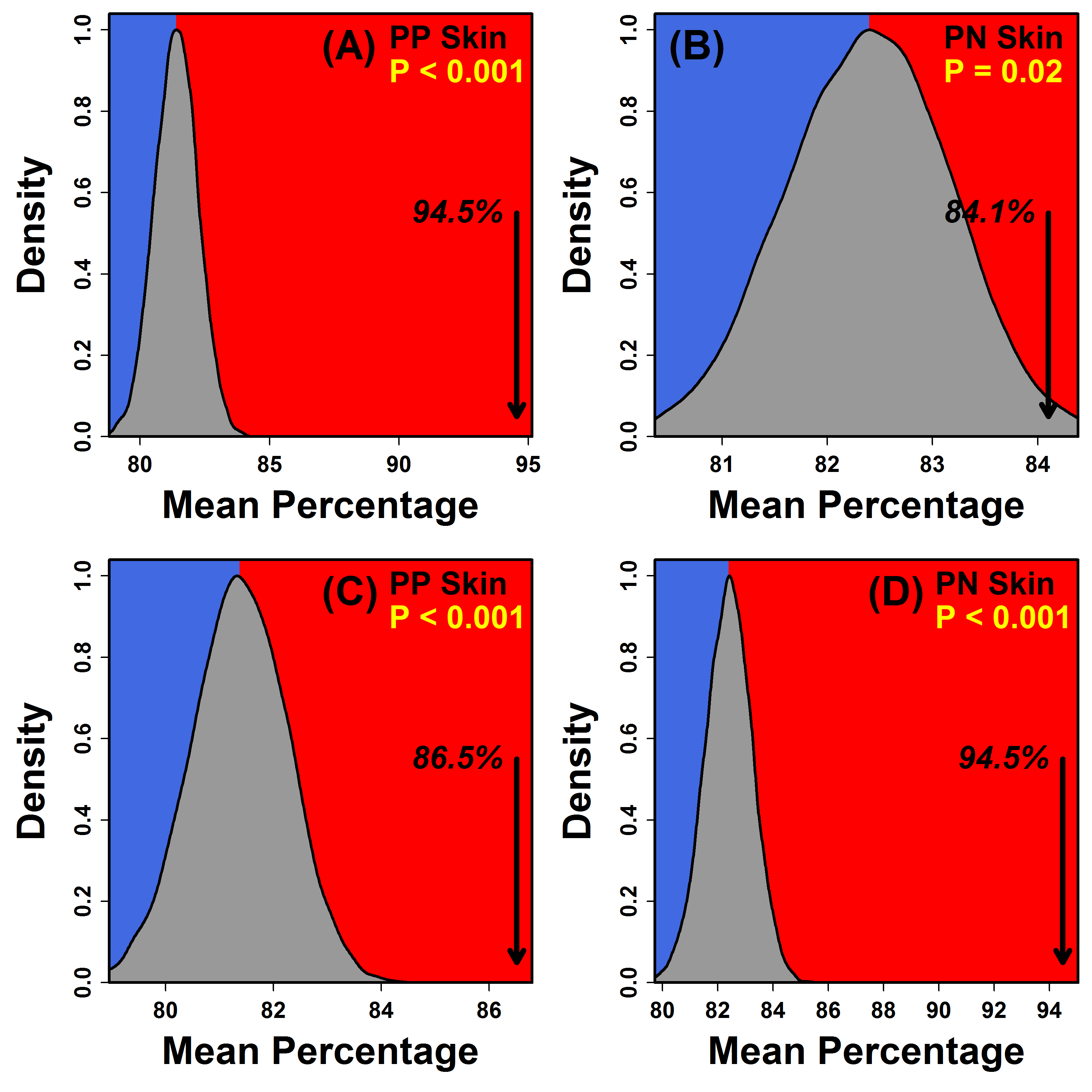

Supplement: Additional file 3 — Psoriasis DEGs are (on average) detected in most (>84%) PP and PN skin samples (P ≤ 0.02). Figures (A) – (D) show the average detection frequency of psoriasis DEGs (black arrow) as compared to a null distribution generated by random sampling from all skin-expressed genes. (A) The 1019 PP-increased DEGs were detected in 94.5% of PP samples on average. This percentage value was significantly larger than expected based upon 10000 random samples of 1019 genes. (B) The 1019 PP-increased DEGs were detected in 84.1% of PN samples on average (P = 0.02, 10000 random samples of 1019 genes). (C) The 885 PP-decreased DEGs were detected in 86.5% of PP samples on average (P < 0.001, 10000 random samples of 885 genes). (D) The 885 PP-decreased DEGs were detected in 94.5% of PN samples on average (P < 0.001, 10000 random samples of 885 genes). [file 1755-8794-7-27-S3.tiff]

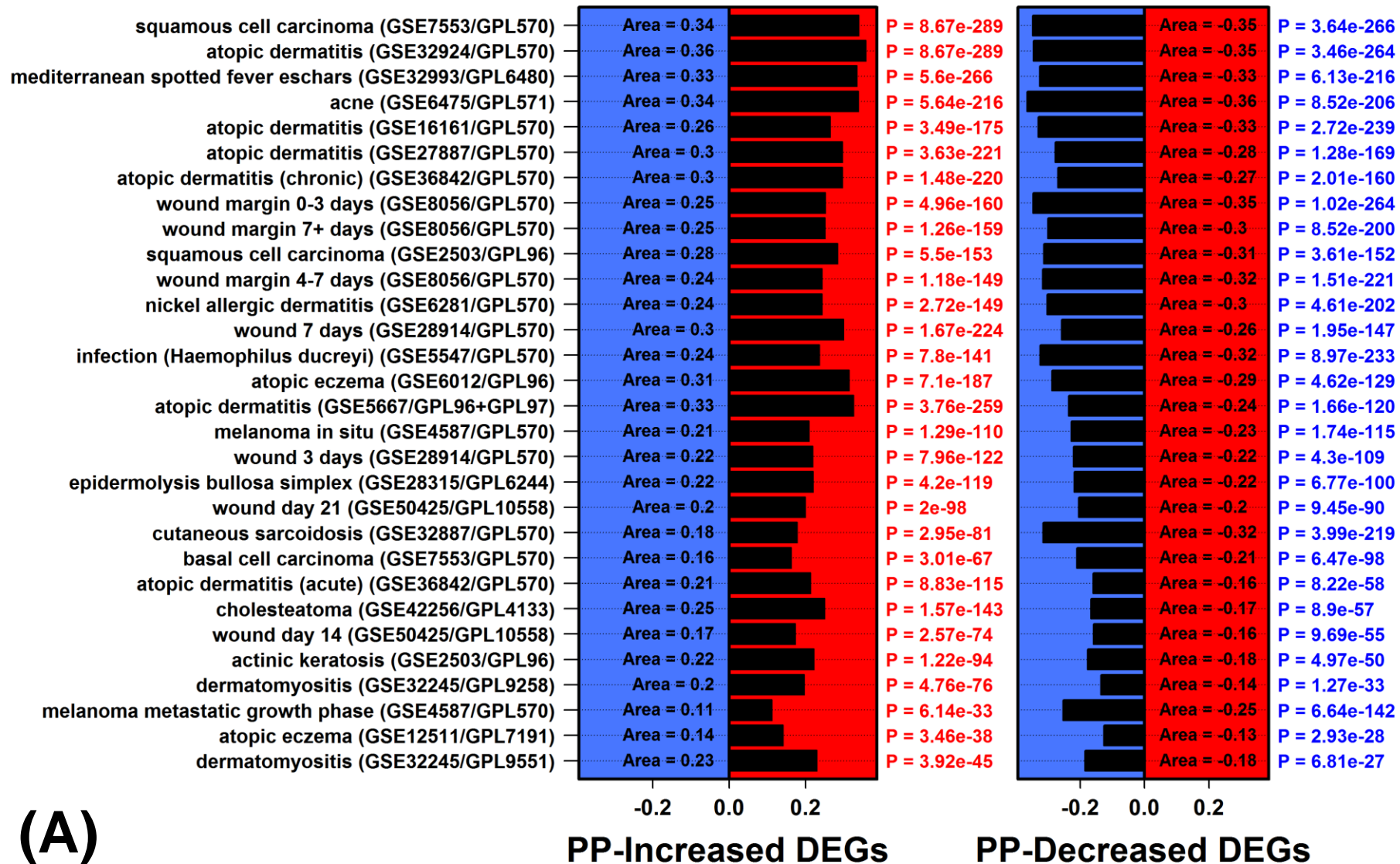

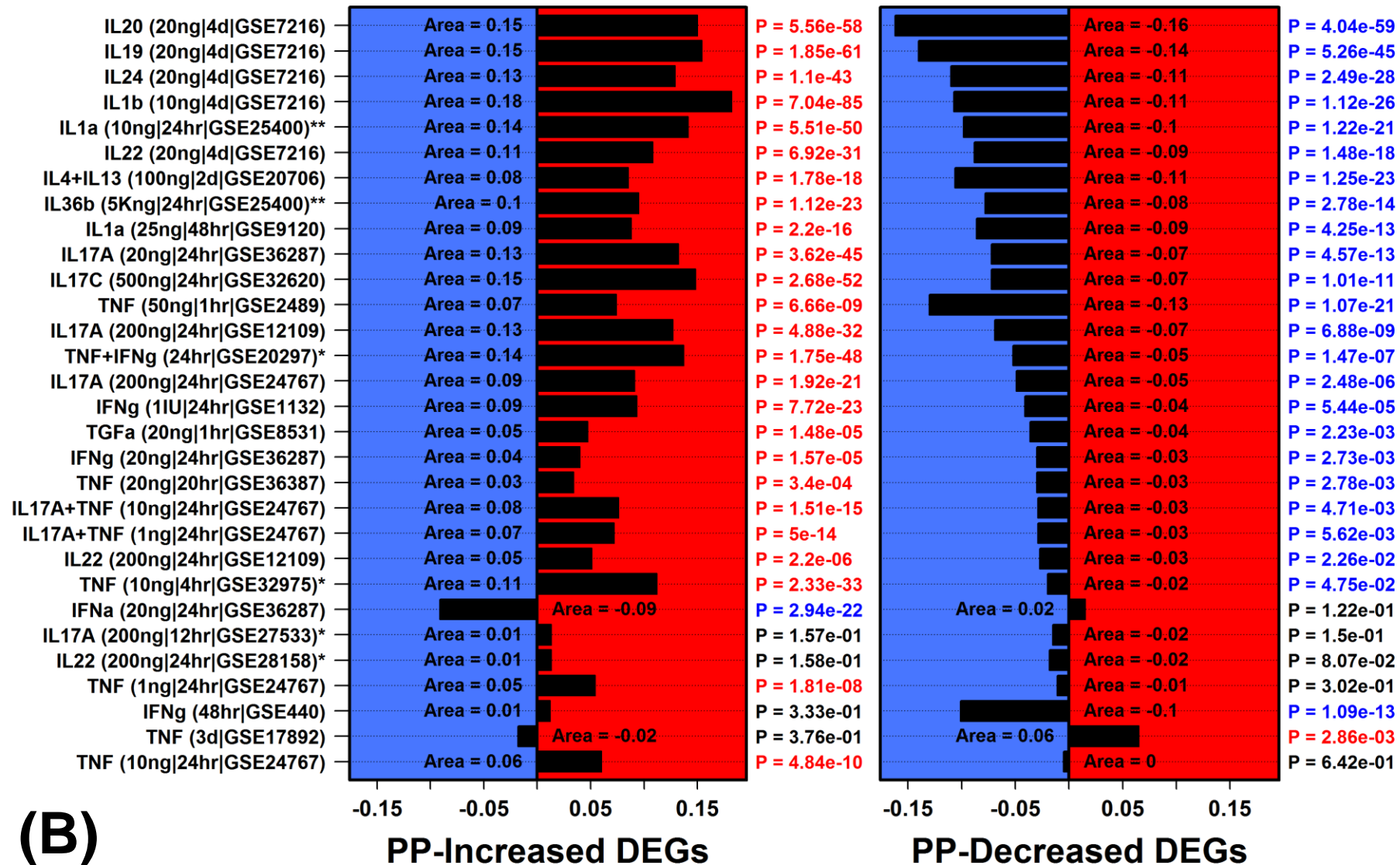

(C)

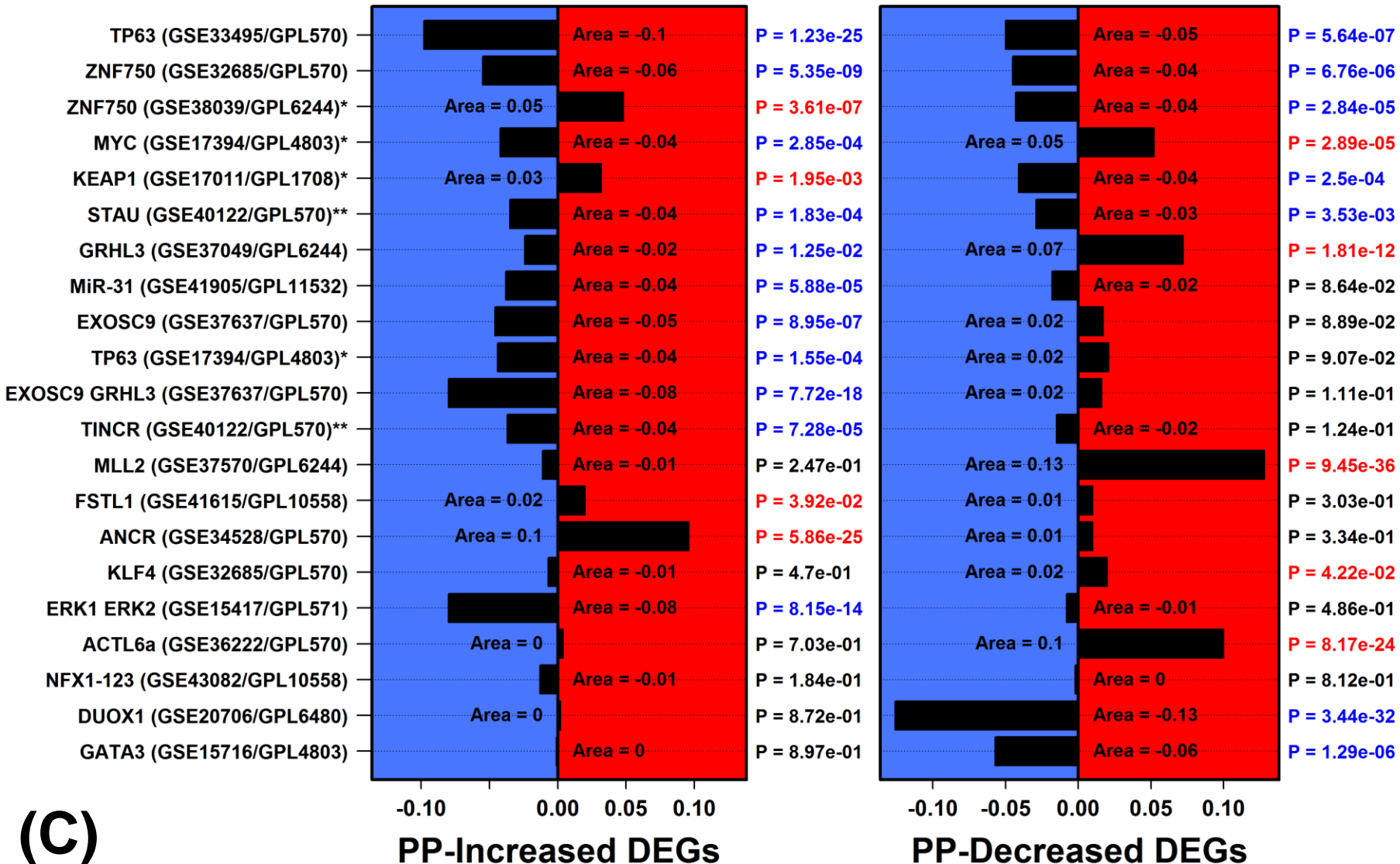

Supplement: Additional file 4 — Gene set enrichment analysis: Psoriasis DEGs overlap significantly with genes altered in various skin conditions, cytokine-treated KCs, and RNAi-treated KCs. (A) Comparison with skin diseases. Expanded results from Figure 1A with the top-ranked 30 comparisons (of 51 included in our screen). (B) Comparison with cytokine-treated KCs. Expanded results from Figure 1B with the top-ranked 30 comparisons (of 46 included in our screen). (C) RNAi experiments. Ranked gene lists were generated from experiments in which microarrays were used to evaluate gene expression in KCs following RNAi. The RNAi target is listed in the label for each experiment (left margin). In both (B) and (C), one asterisk (*) denotes experiments performed with HaCaT KCs, while two asterisks (**) denotes experiments performed using reconstituted epidermis. All other experiments were performed with primary NHEKs. [file 1755-8794-7-27-S4.pdf]

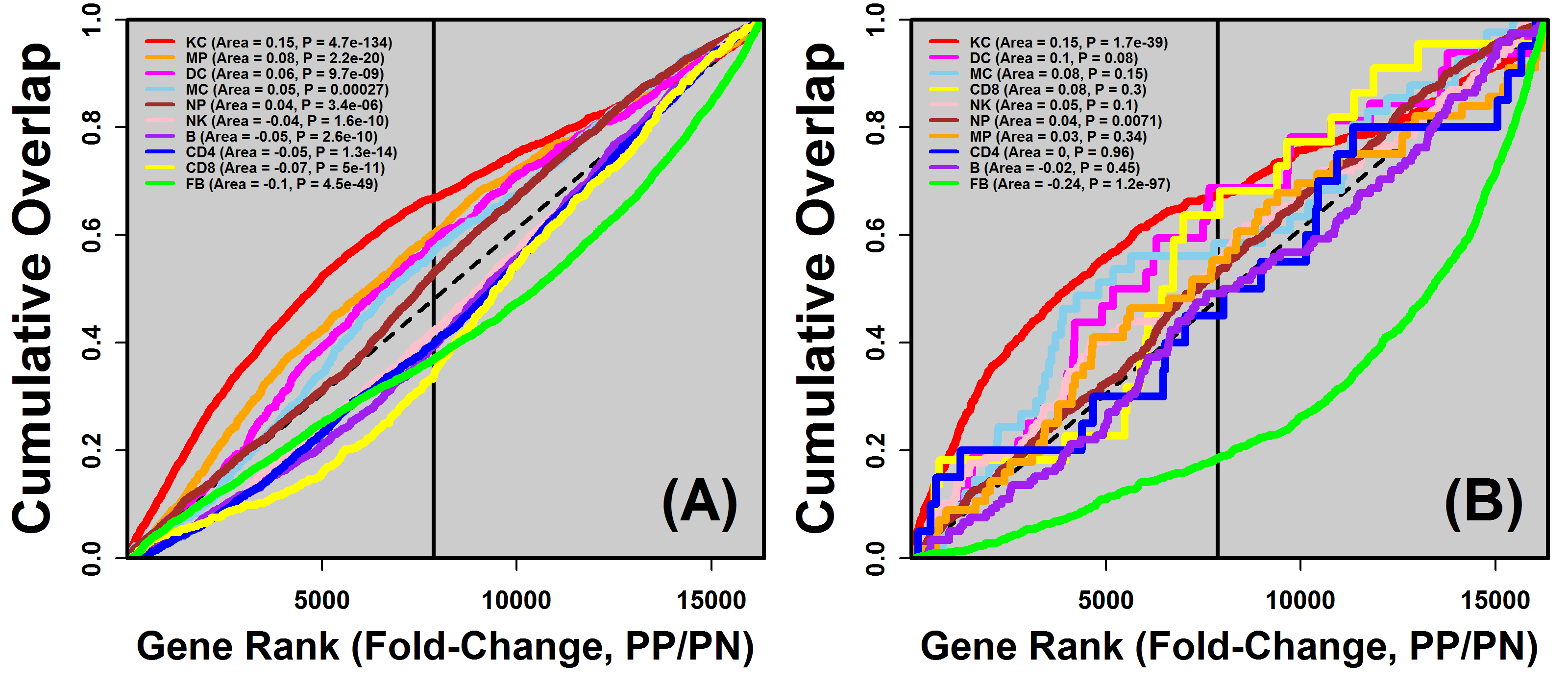

Supplement: Additional file 6 — Genes assigned to each of 10 cell types show bias towards PP-increased or PP-decreased expression. (A) The 16358 skin-expressed genes were ranked according to the estimated median PP/PN fold-change (n = 216 patients, horizontal axis). For each cell type, the line tracks the cumulative overlap between top-ranked genes and the set of genes assigned to that cell type. The area calculated for each cell type represents the region between the diagonal line (random expectation) and the line drawn for each cell type, respectively. P-values were generated from the Wilcoxon rank sum test. (B) The same analysis was repeated, except only genes showing a cell type-specific expression pattern were included, with expression in the assigned cell type significantly higher than expression in each of the other 9 cell types (FDR < 0.05; Wilcoxon rank sum test). [file 1755-8794-7-27-S6.tiff]

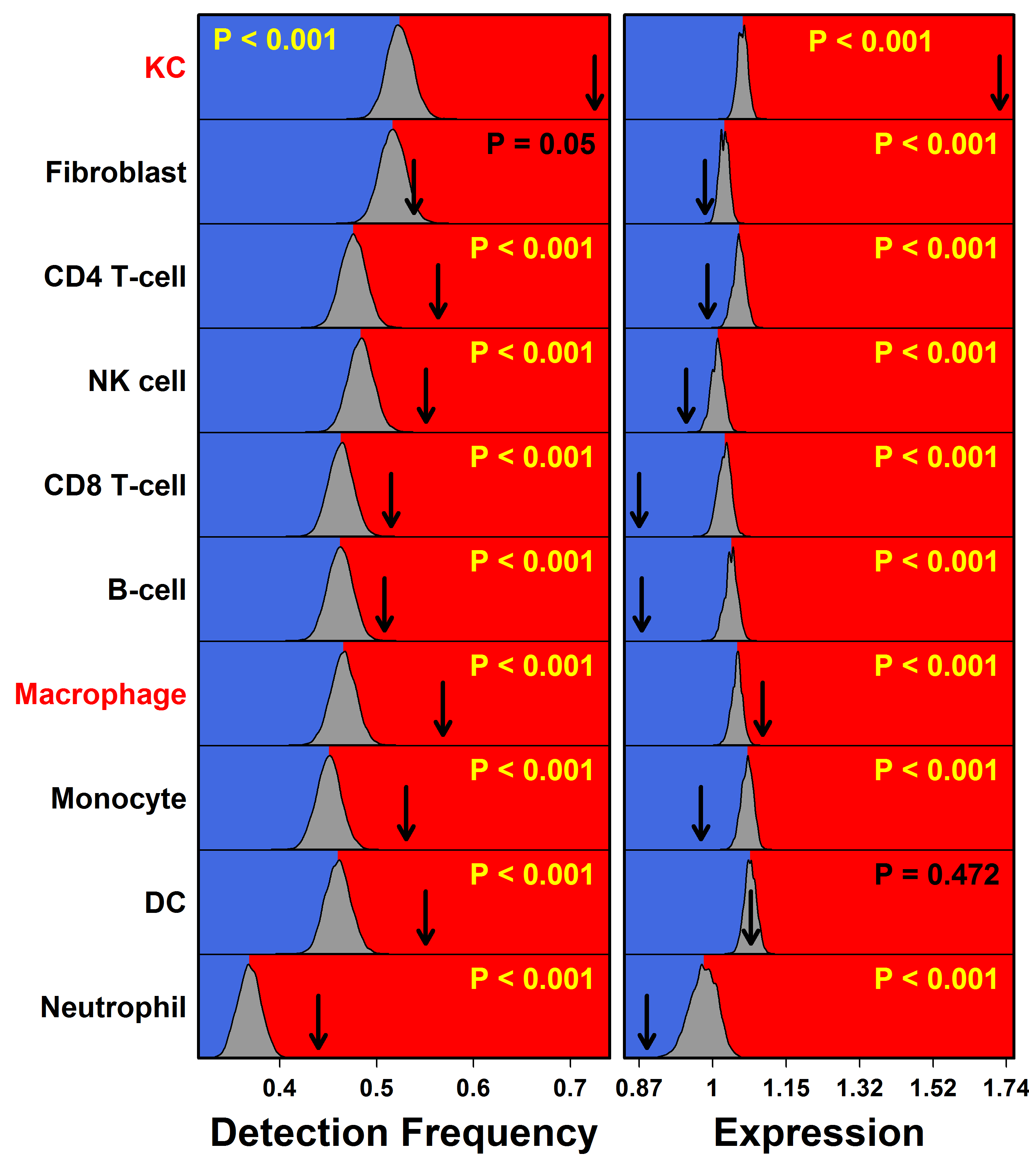

Supplement: Additional file 7 — PP-increased DEGs show aberrantly high expression in KCs and macrophages. We identified 1019 PP-increased DEGs (median FC > 1.50, FDR < 0.05; Wilcoxon rank sum test; n = 216 patients). The average detection frequency among the 1019 DEGs was calculated for each cell type (black arrows, left column). Likewise, the median expression among the 1019 DEGs was calculated with respect to each cell type (black arrows, right column). Null distributions for the average detection frequency and median expression level were generated based upon 10000 samples of 1019 genes chosen randomly from 16358 skin-expressed genes. P-values were calculated using these empirical null distributions and are shown in the figure. Expression values (right column) are normalized to normal human skin, such that values greater than 1 indicate that expression is greater than that in normal skin, while values less than 1 indicate that expression is lower than that in normal skin. [file 1755-8794-7-27-S7.tiff]

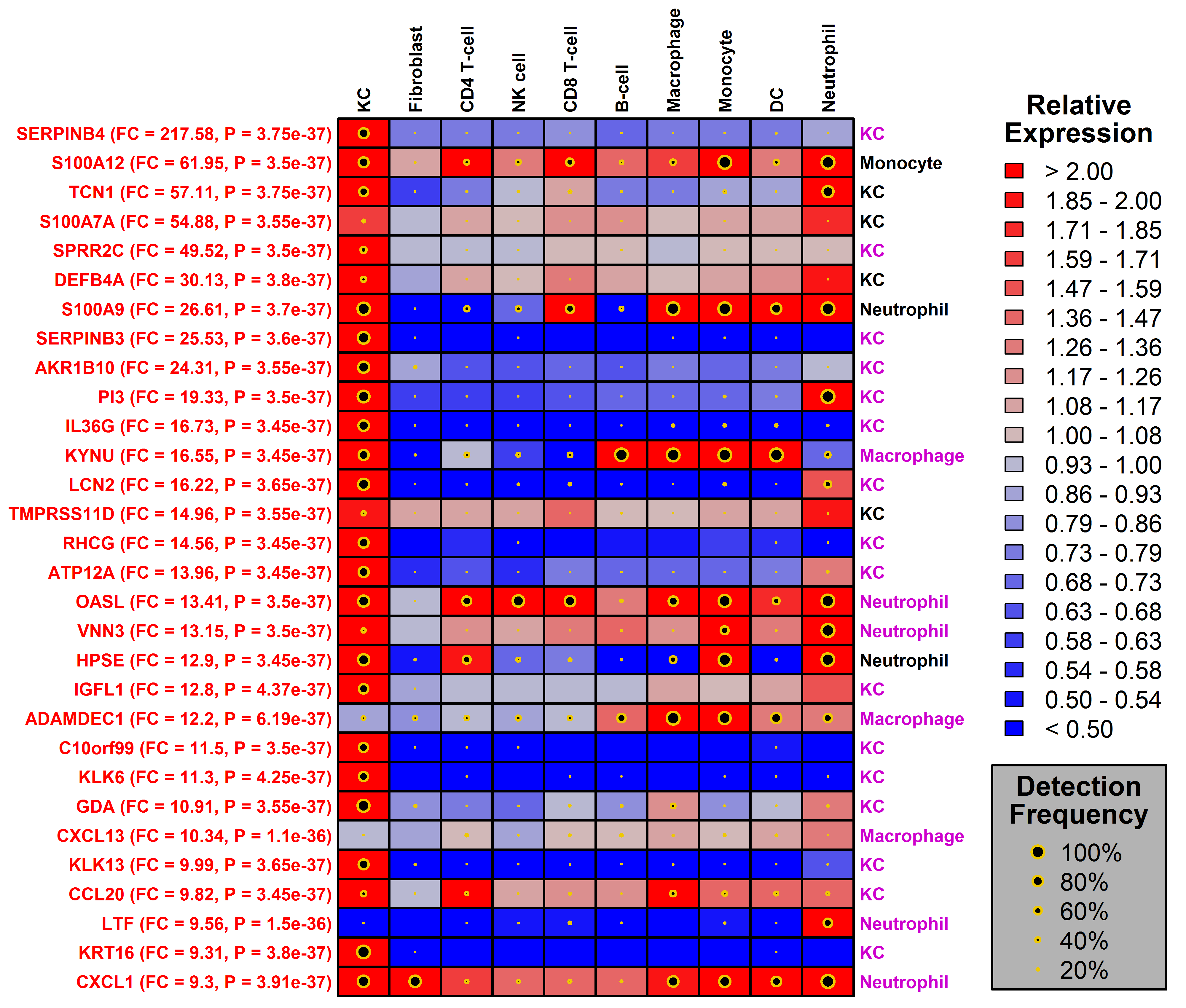

Supplement: Additional file 8 — Top 30 genes most strongly elevated in psoriasis lesions ( n = 216 patients) and their expression across 10 cell types. The table lists the 30 DEGs most strongly elevated in psoriasis lesions (i.e., highest median PP/PN fold-change, with FDR < 0.05). The color scale denotes median expression of each gene, as compared to the gene’s median expression in normal human skin. Red colors thus denote expression that is higher in comparison to normal human skin, while blue colors indicate that expression is lower in comparison to normal human skin. For each gene and cell type, black circles indicate the percentage of microarray samples for which the gene’s expression was detected above background (P < 0.05, Wilcoxon signed-rank test). The cell type assigned to each gene is listed in the right margin. Magenta labels denote cases in which the gene’s expression in the assigned cell type is significantly higher than its expression in any of the other 9 cell types (FDR < 0.05, Wilcoxon rank sum test). [file 1755-8794-7-27-S8.tiff]

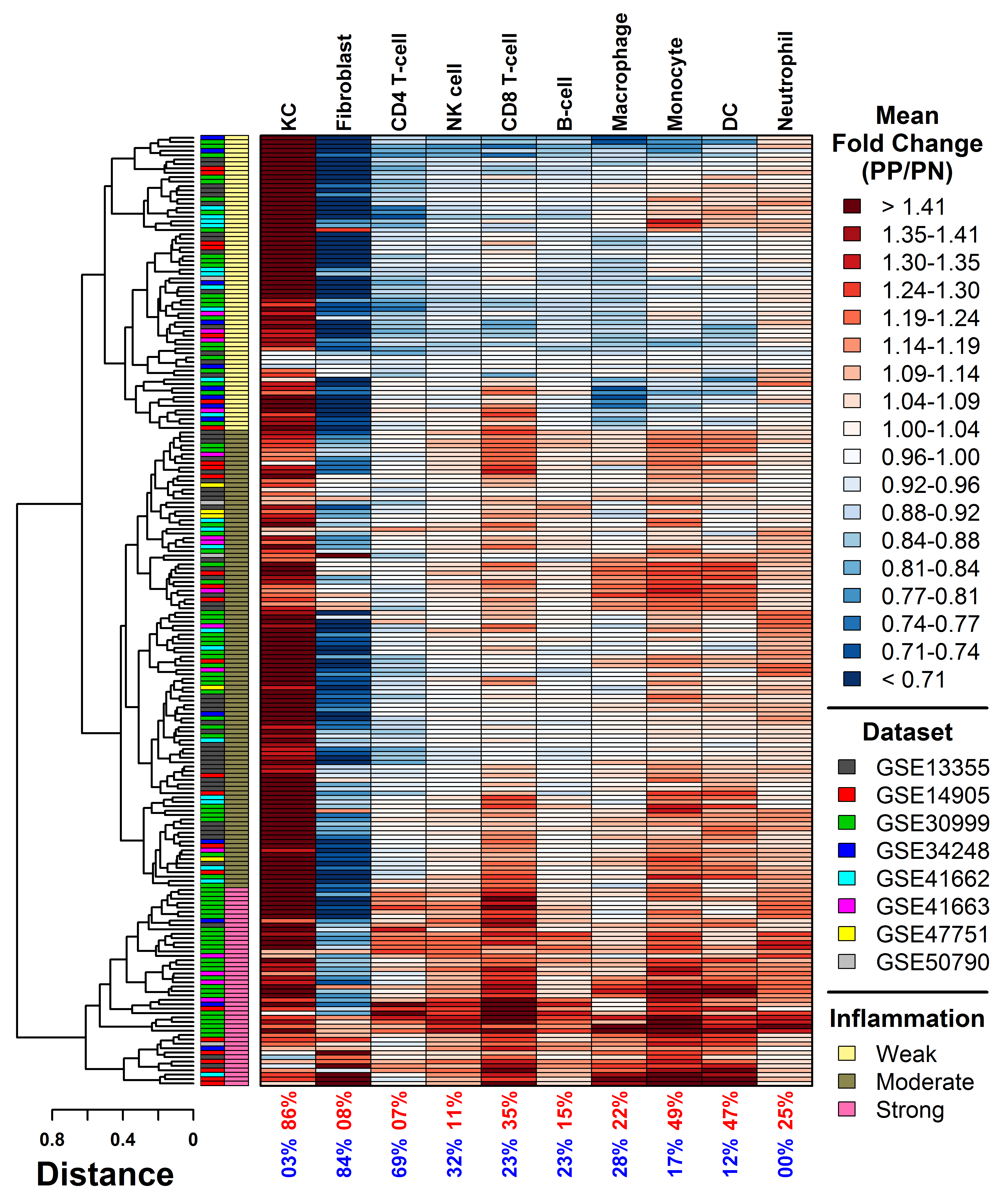

Supplement: Additional file 9 — Psoriasis lesions from 216 patients vary with respect to the expression of genes specifically expressed by immune cell types. Cell type-specific signature scores were calculated for each patient and each cell type. Signature scores for a cell type were calculated based upon a set of 250 genes with high expression in that cell type (as compared to the other 9 cell types), with scores equal to the weighted average of fold-change values (PP/PN) among the 250 genes for a given patient (weighted arithmetic mean). Genes showing the most cell type-specific expression pattern were assigned the greatest weight in these calculations. Patients were then clustered based upon signature scores across the 10 cell types (Euclidean distance with complete linkage). Values in the bottom margin indicate the percentage of patients for which signature scores were significantly large (red, P < 0.05) or significantly small (blue, P < 0.05). Significance of scores was evaluated based upon whether PP/PN fold-changes for the 250 signature genes differed from those of all other skin-expressed genes (Wilcoxon rank sum test). Patients were assigned to one of three groups based upon the patterns observed for immune cell types (weak inflammation, 67/216; moderate inflammation, 104/216; strong inflammation, 45/216). [file 1755-8794-7-27-S9.tiff]

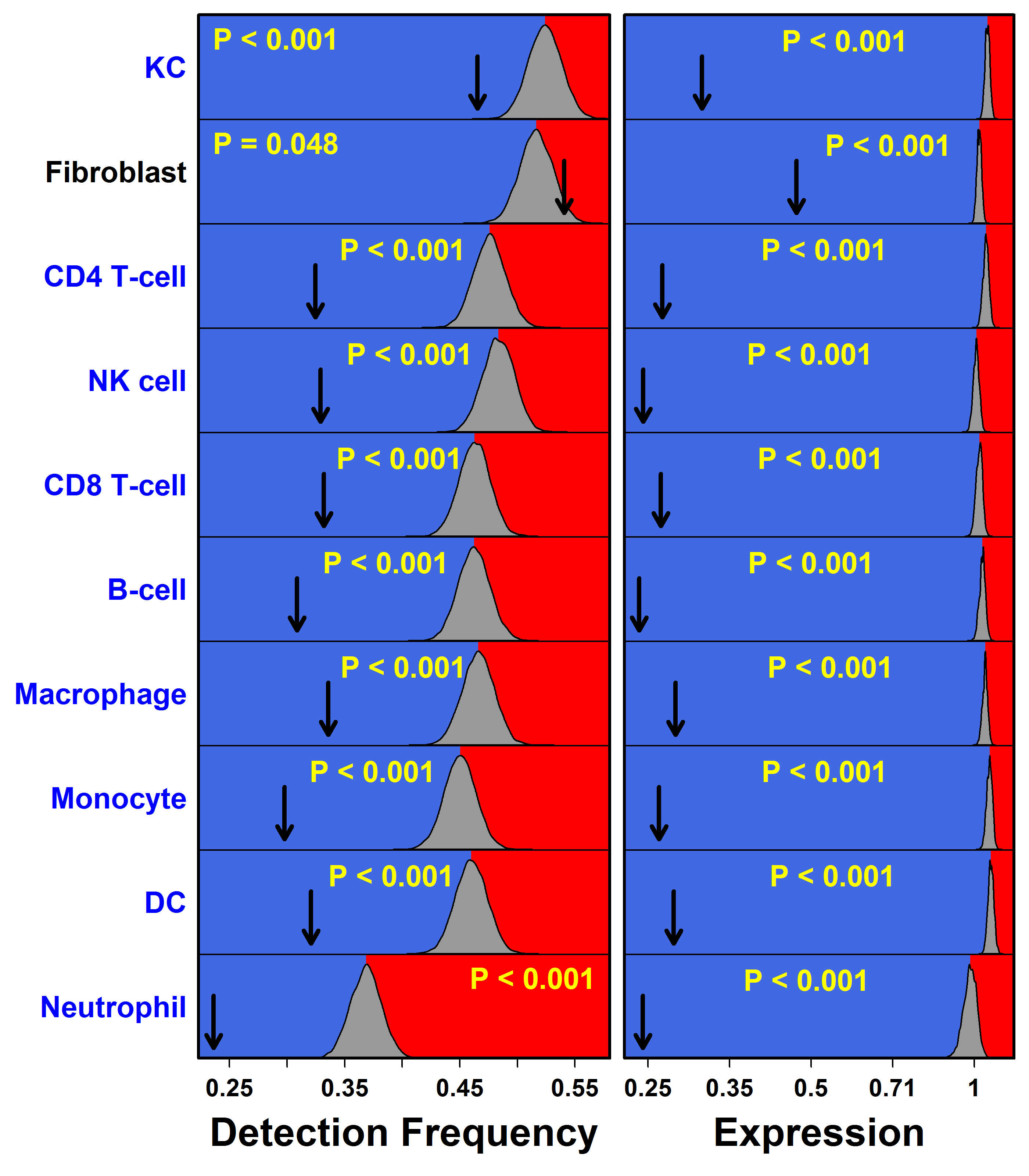

Supplement: Additional file 10 — PP-decreased DEGs show aberrantly low expression in all cell types except fibroblasts. We identified 885 PP-decreased DEGs (median FC > 1.50, FDR < 0.05; Wilcoxon rank sum test; n = 216 patients). The average detection frequency among the 885 DEGs was calculated for each cell type (black arrows, left column). Likewise, the median expression among the 885 DEGs was calculated with respect to each cell type (black arrows, right column). Null distributions for the average detection frequency and median expression level were generated based upon 10000 samples of 885 genes chosen randomly from 16358 skin-expressed genes. P-values were calculated using these empirical null distributions and are shown in the figure. Expression values (right column) are normalized to normal human skin, such that values greater than 1 indicate that expression is greater than that in normal skin, while values less than 1 indicate that expression is lower than that in normal skin. [file 1755-8794-7-27-S10.tiff]

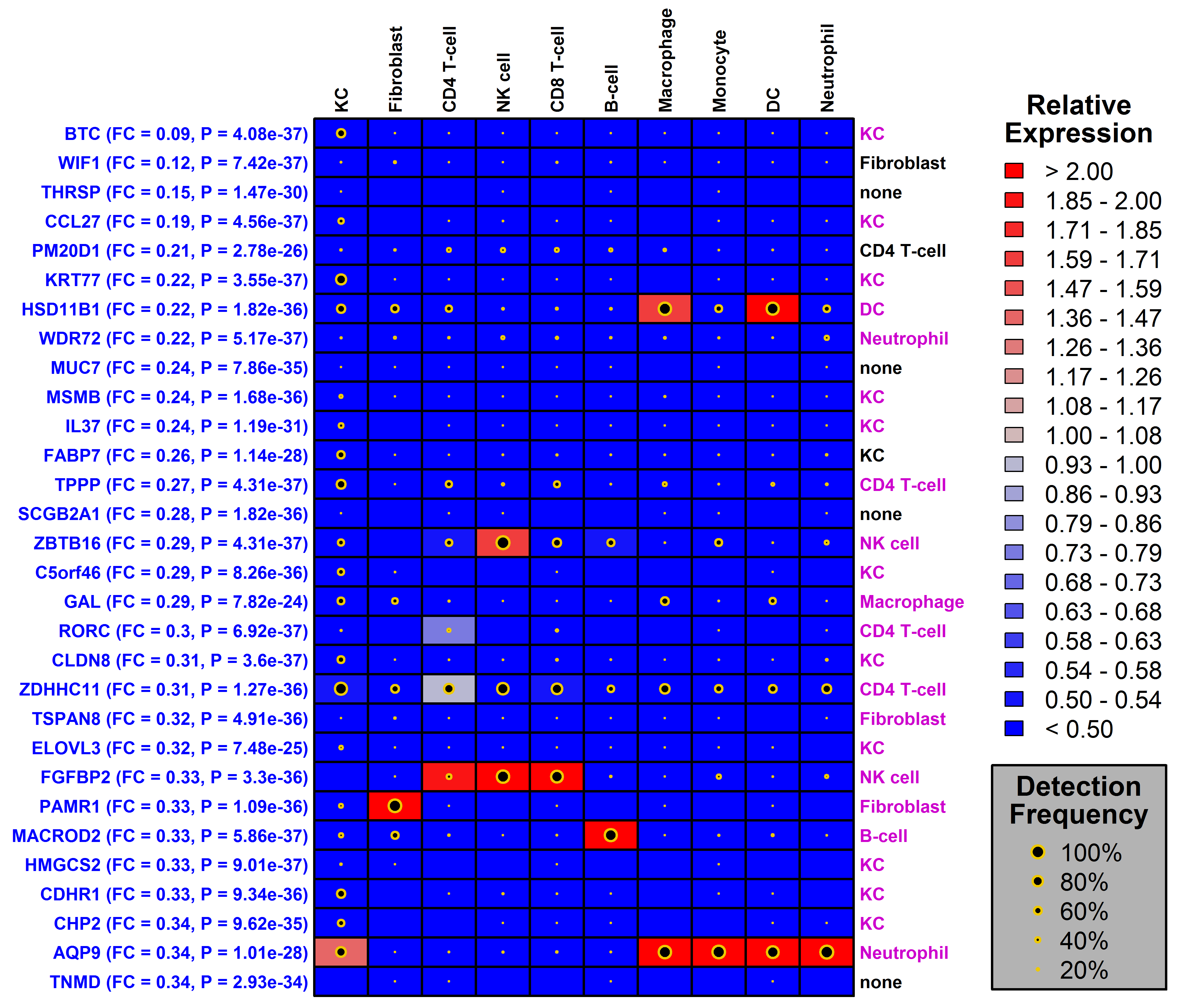

Supplement: Additional file 11 — Top 30 genes most strongly decreased in psoriasis lesions ( n = 216 patients) and their expression across 10 cell types. The table lists the 30 DEGs most strongly decreased in psoriasis lesions (i.e., lowest median PP/PN fold-change, with FDR < 0.05). The color scale denotes median expression of each gene, as compared to the gene’s median expression in normal human skin. Red colors thus denote expression that is high in comparison to normal human skin, while blue colors indicate that expression is low in comparison to normal human skin. For each gene and cell type, black circles indicate the percentage of microarray samples for which the gene’s expression was detected above background (Wilcoxon signed-rank test; P < 0.05). The cell type assigned to each gene is listed in the right margin. This is the cell type for which the gene’s median expression level was the highest, provided that the gene’s detection frequency for that cell type was greater than 10%. Magenta labels denote cases in which the gene’s expression in the assigned cell type is significantly higher than its expression in each of the other 9 cell types, respectively (FDR < 0.05, Wilcoxon rank sum test). [file 1755-8794-7-27-S11.tiff]

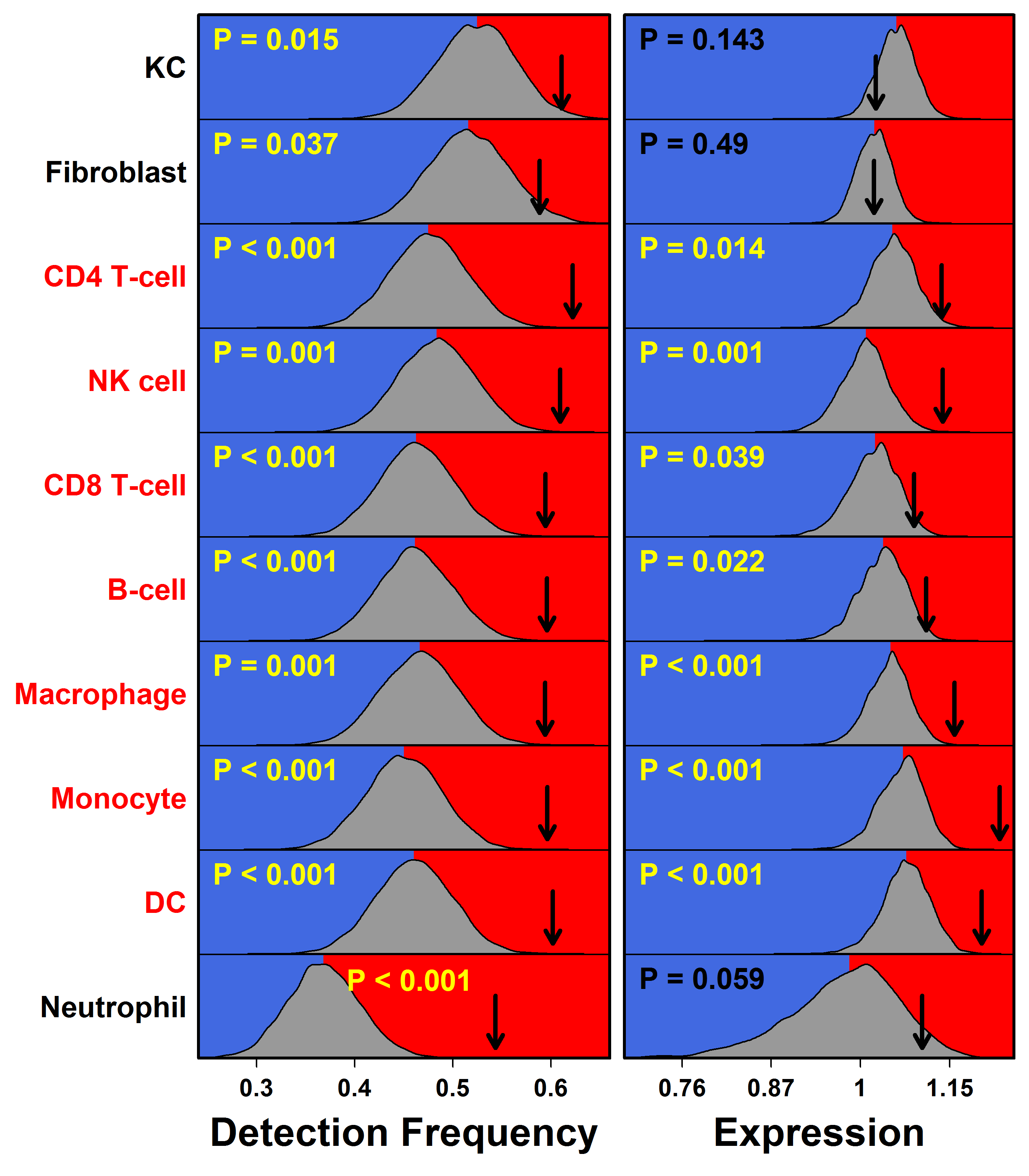

Supplement: Additional file 12 — Candidate genes from psoriasis GWAS studies show aberrantly high expression in immune cell populations. We identified 117 candidate genes near GWAS loci. The average detection frequency among these 117 genes was calculated for each cell type (black arrows, left column). Likewise, the median expression among the 117 genes was calculated with respect to each cell type (black arrows, right column). Null distributions for the average detection frequency and median expression level were generated based upon 10000 samples of 117 genes chosen randomly from the 20184 human genes represented on the Affymetrix Human Genome U133 Plus 2.0 array. P-values were calculated using these empirical null distributions and are shown in the figure. Expression values (right column) are normalized to normal human skin, such that values greater than 1 indicate that expression is greater than that in normal skin, while values less than 1 indicate that expression is lower than that in normal skin. [file 1755-8794-7-27-S12.tiff]

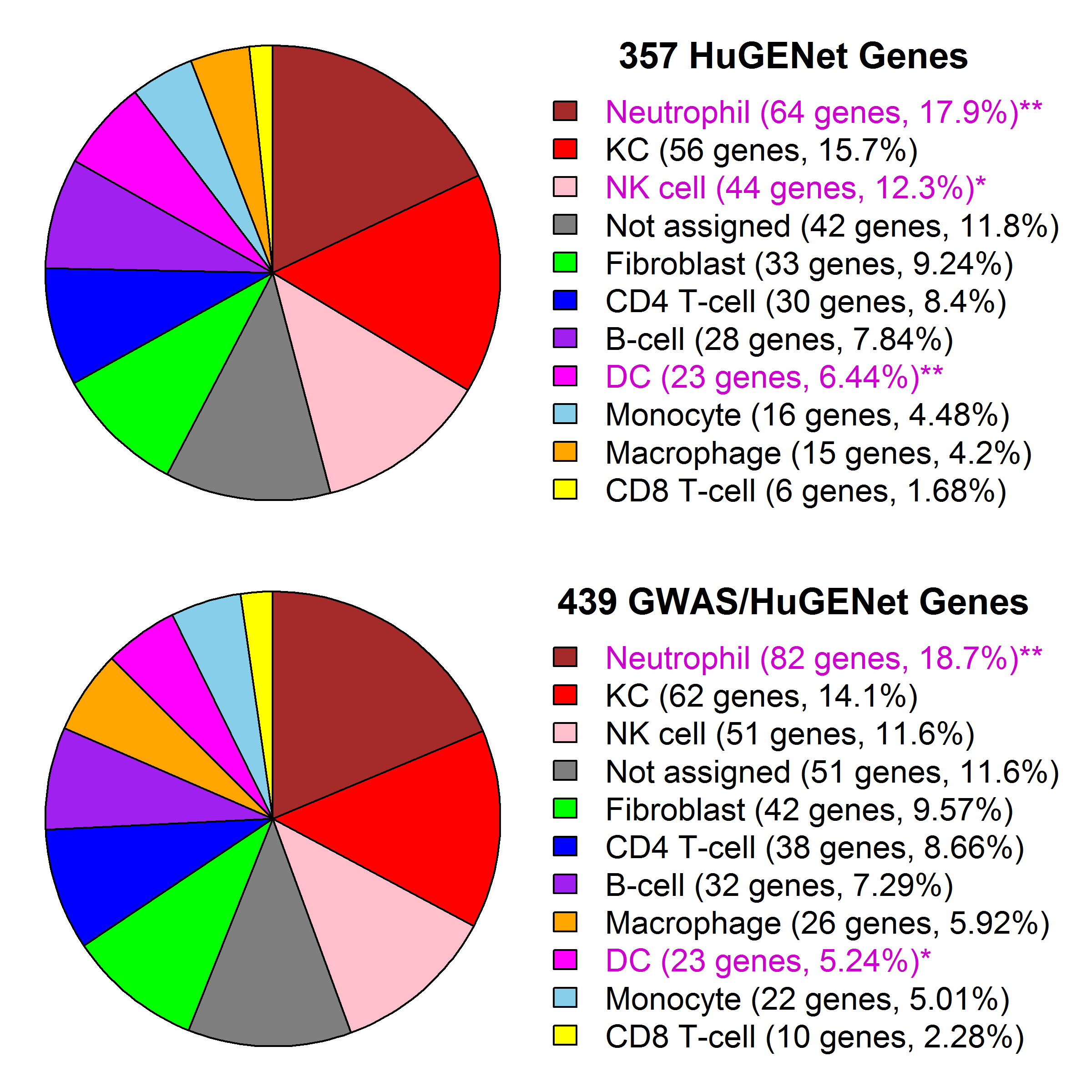

Supplement: Additional file 13 — Assignment of gene candidates from genetic studies to cell types present in lesional skin (HuGENet database). Human genes were assigned to one of 10 cell types present in psoriasis lesions (see Results and Methods). Top: Analysis of 357 genes associated with psoriasis in either GWAS or non-GWAS population-based studies (HuGENet database). Bottom: Combined analysis of the 357 genes associated with psoriasis from the HuGENet database and the set of 117 GWAS-only gene candidates (439 unique genes total). Magenta labels denote those cell types for which the number of assigned genes was significantly large in comparison to known human genes (i.e., all genes represented on the Affymetrix Human Genome U133 Plus 2.0 array platform) (one asterisk, P < 0.05; two asterisks, FDR < 0.05; Fisher’s Exact Test). [file 1755-8794-7-27-S13.tiff]

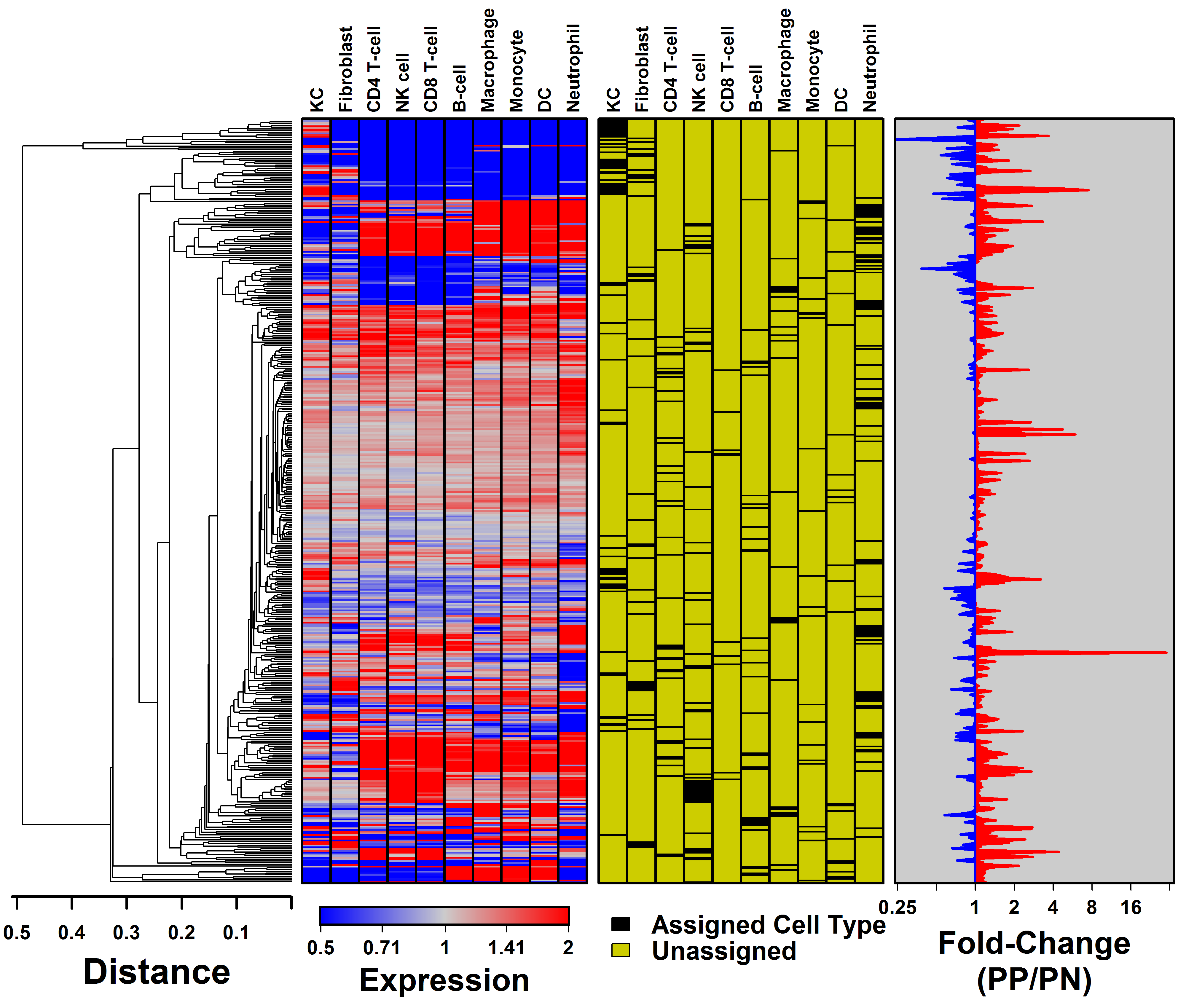

Supplement: Additional file 14 — Cluster analysis of 439 genes genetically associated with psoriasis in population-based genetic studies and their expression across 10 cell types. We identified 117 genes near candidate loci identified from GWAS studies and combined these with a set of 357 genes associated with psoriasis (GWAS or non-GWAS studies) in the HuGENet database (439 genes total). These genes were clustered based upon their expression pattern across 10 cell types (Euclidean distance and average linkage). The red-blue heatmap shows the expression of genes in each cell type, with red colors indicating relatively high expression (compared to normal human skin) and blue colors indicating relatively low expression (compared to normal human skin). The yellow-black heatmap shows the cell type assigned to each gene (i.e., the cell type for which the gene’s median expression was highest, with detection frequency greater than 10%). The chart on the far right shows the estimated median fold-change (PP/PN) for each gene (n = 216 patients). [file 1755-8794-7-27-S14.tiff]

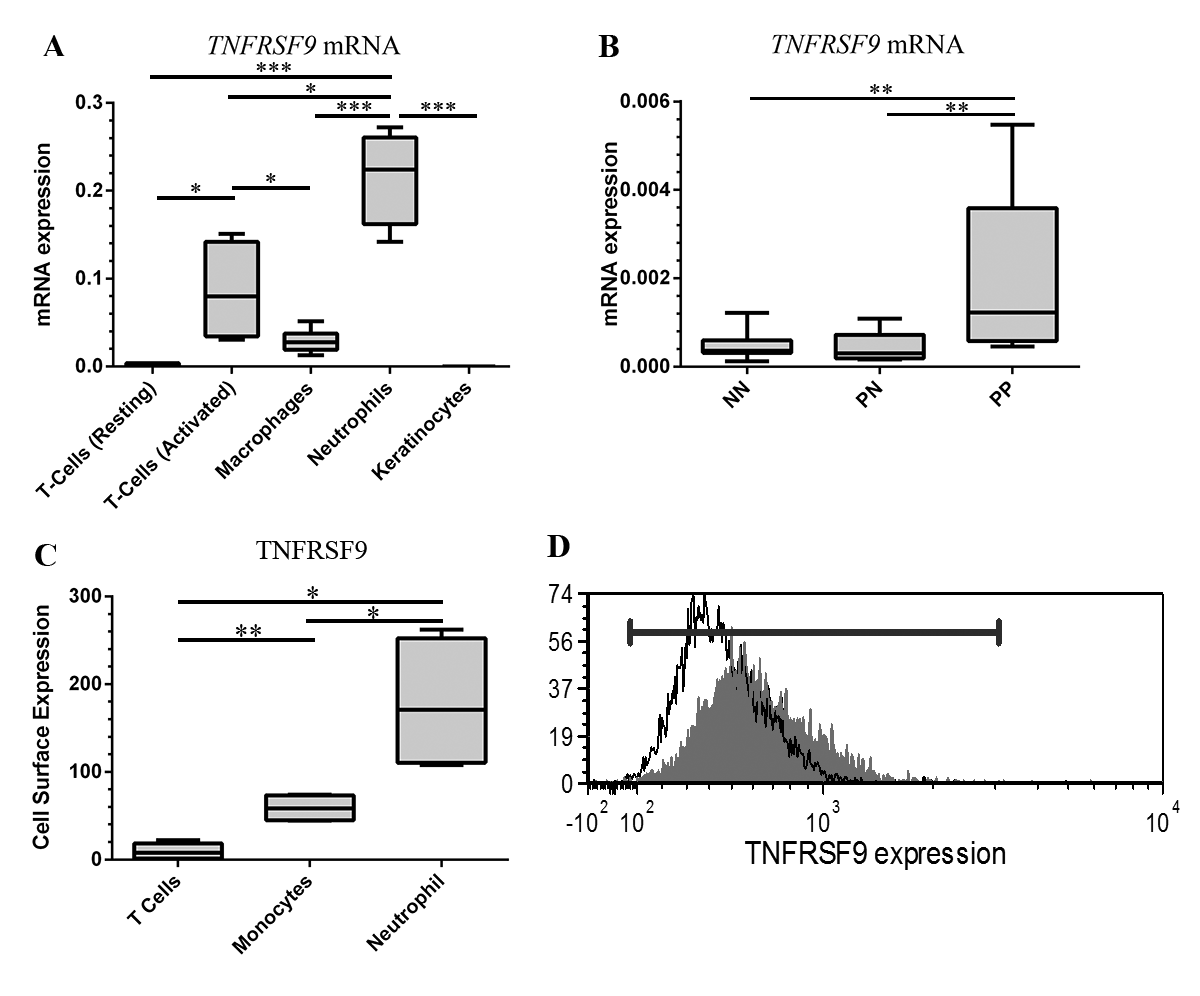

Supplement: Additional file 15 — TNFRSF9 mRNA is elevated in psoriasis lesions and TNFRSF9 mRNA and protein levels are highest in neutrophils as compared to other cell types. (A) TNFRSF9 mRNA expression in five cell types (n > 4 for each cell type). (B) TNFRSF9 mRNA is significantly elevated in psoriasis lesions as compared to uninvolved and normal skin (n = 11). (C) TNFRS9 (CD137) protein expression is highest in neutrophils as compared to T-cells and monocytes isolated from the peripheral blood. (D) Flow cytometric analysis shows TNFRSF9-positive neutrophils over isotype control (negative cells). In parts (A) – (C), expression is normalized to the housekeeping gene large ribosomal protein P0 (RPLP0). Bars represent mean ± 1SD (*P < 0.05, **P < 0.01, ***P < 0.001). [file 1755-8794-7-27-S15.tiff]

# (A) *TNFRSF9* (rs11121129, Chr 1, 8268095)

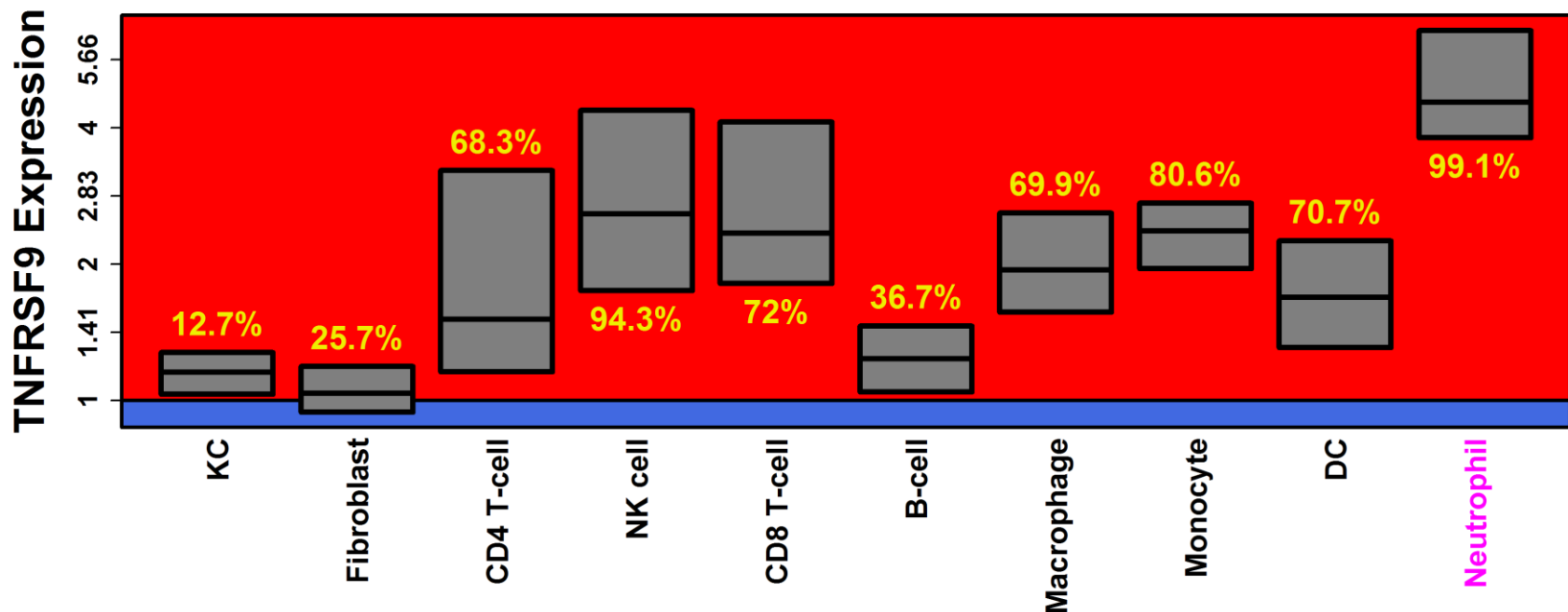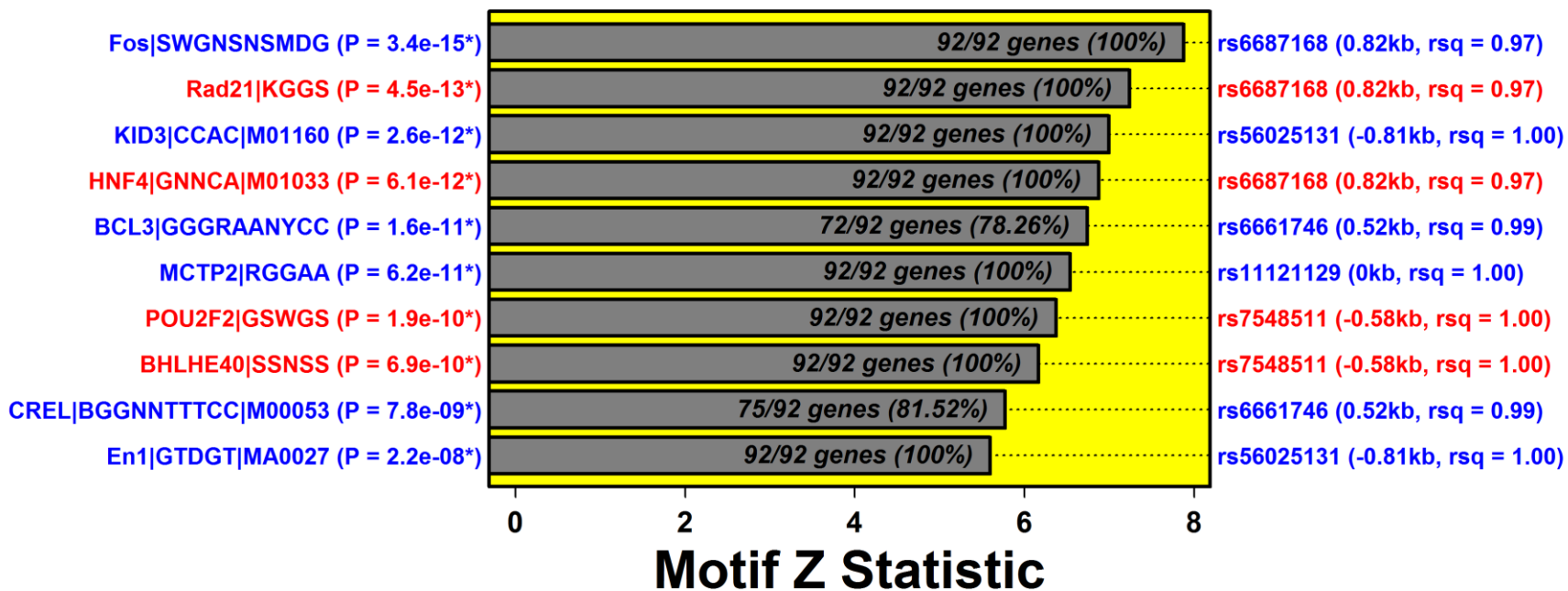

**(B) *B3GNT2* (rs10865331, Chr 2, 62551472)**

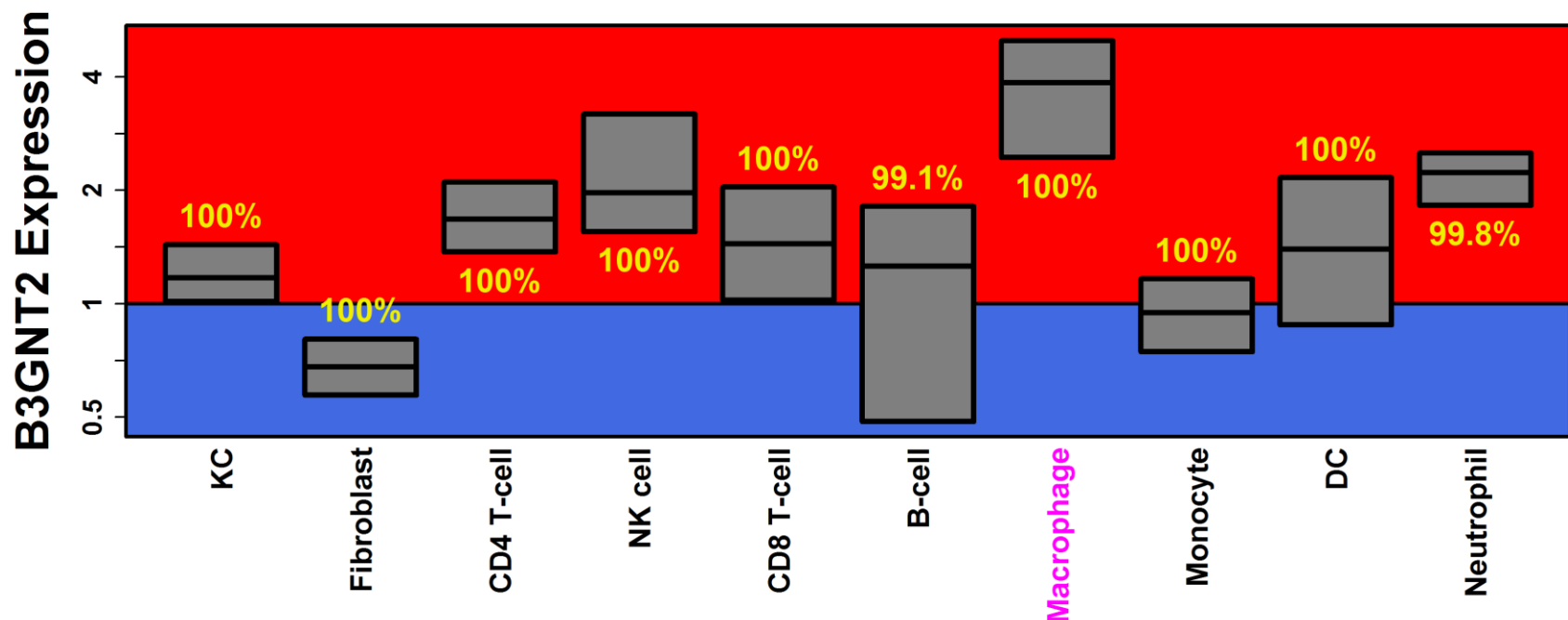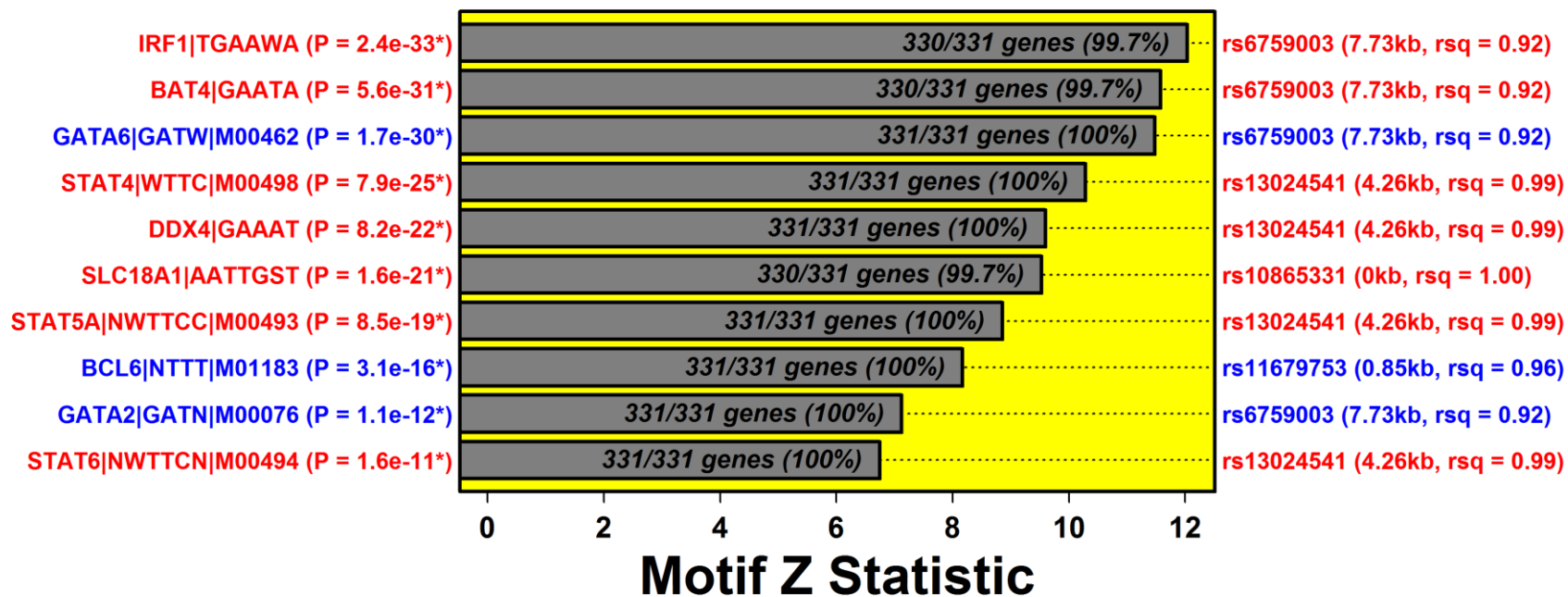

# (C) IL12B (rs12188300, Chr 5, 158829527)

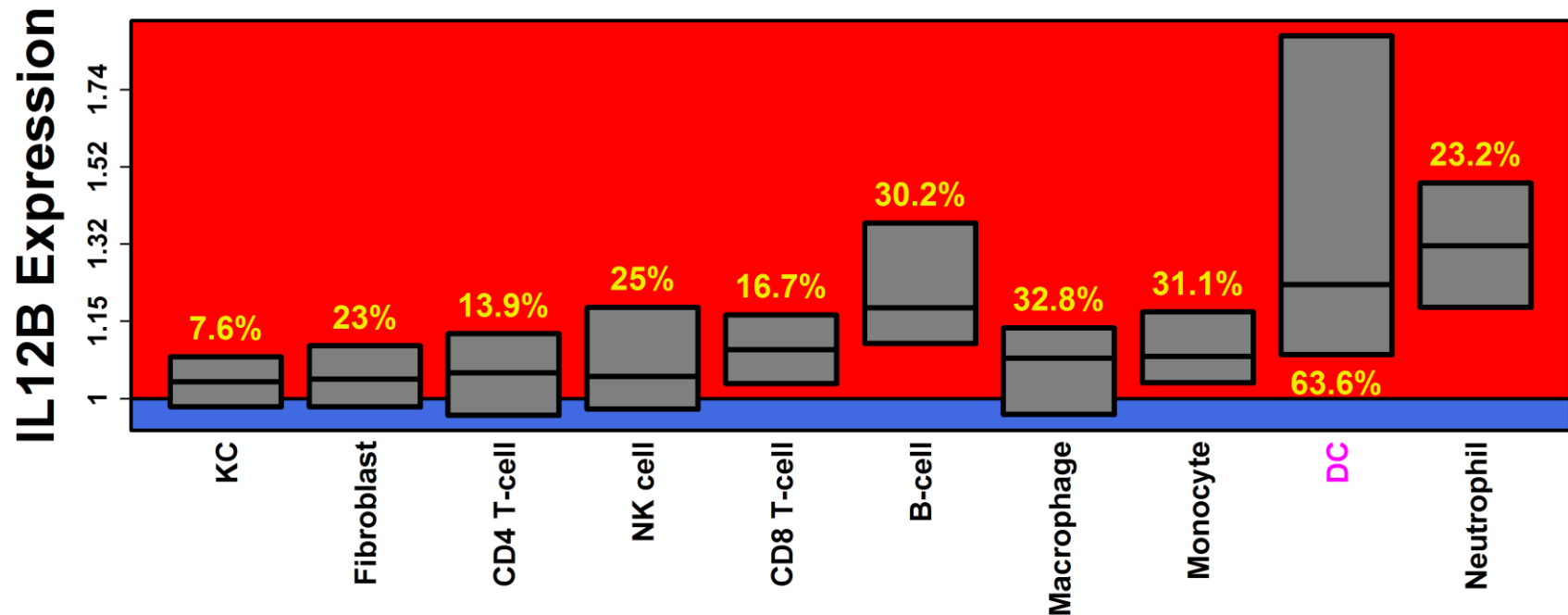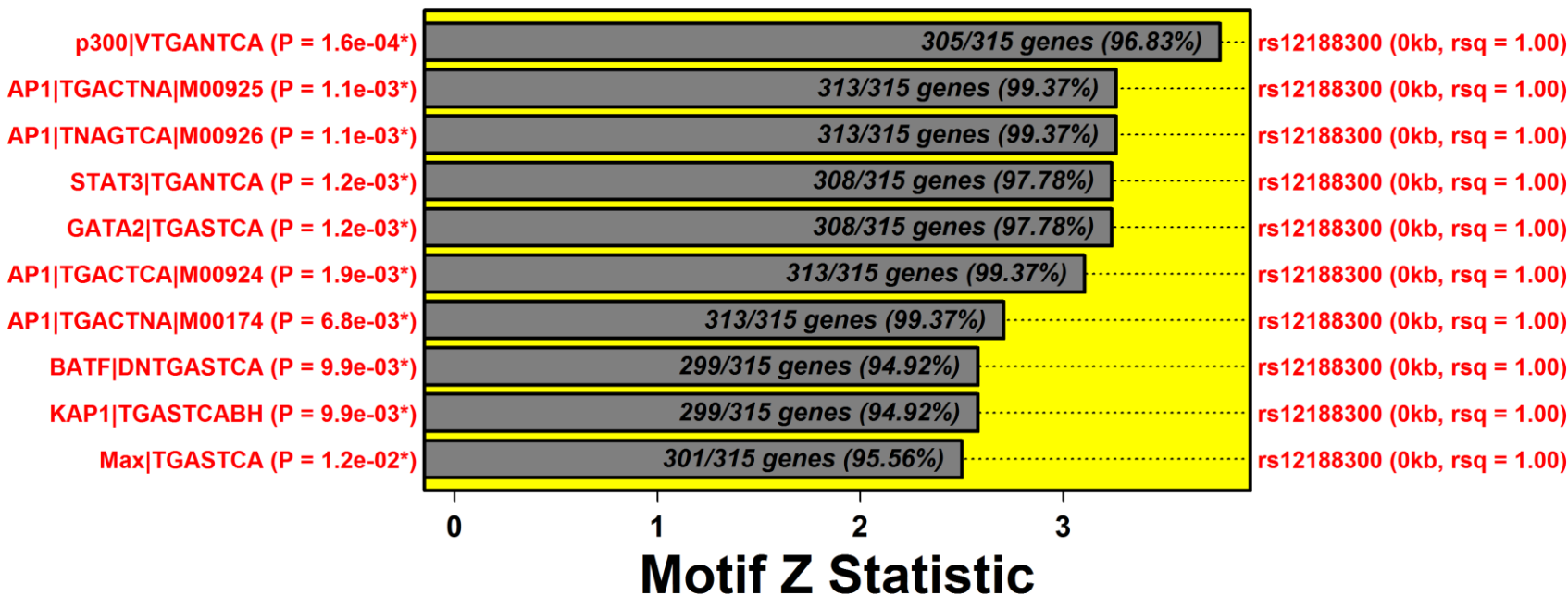

(D) *TAGAP* (rs2451258, Chr 6, 159506600)

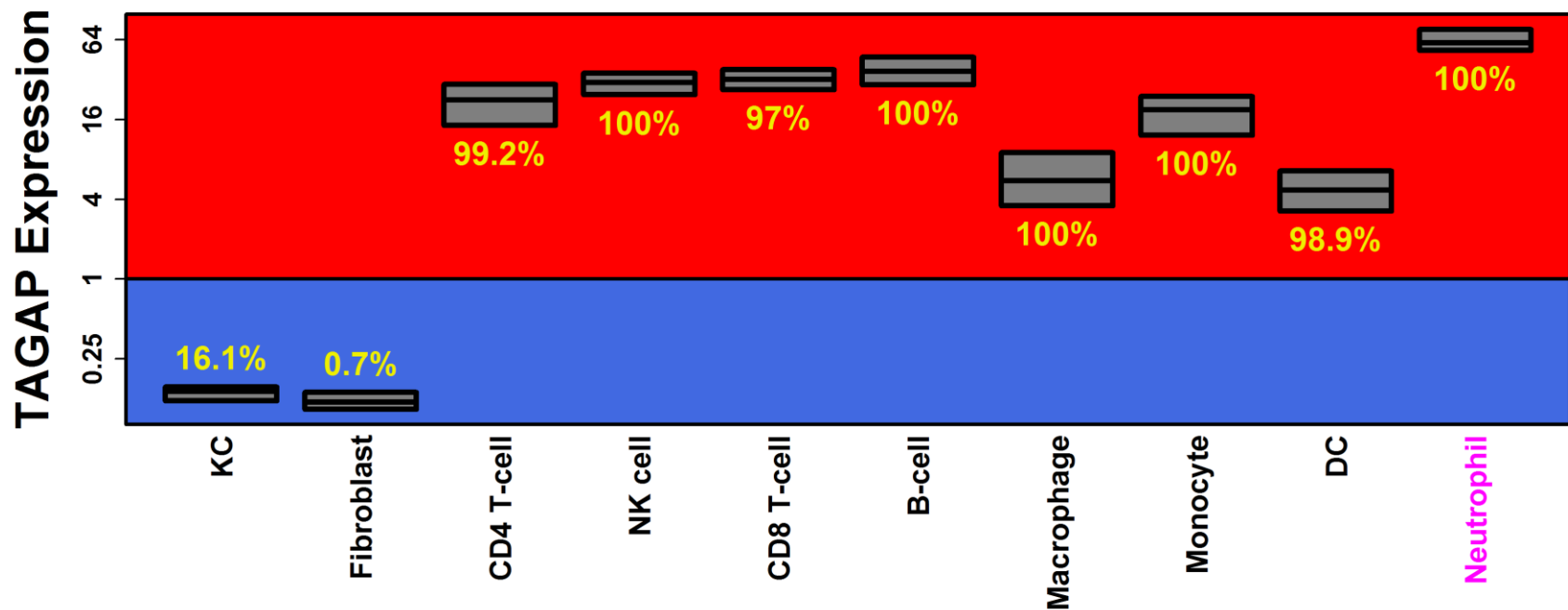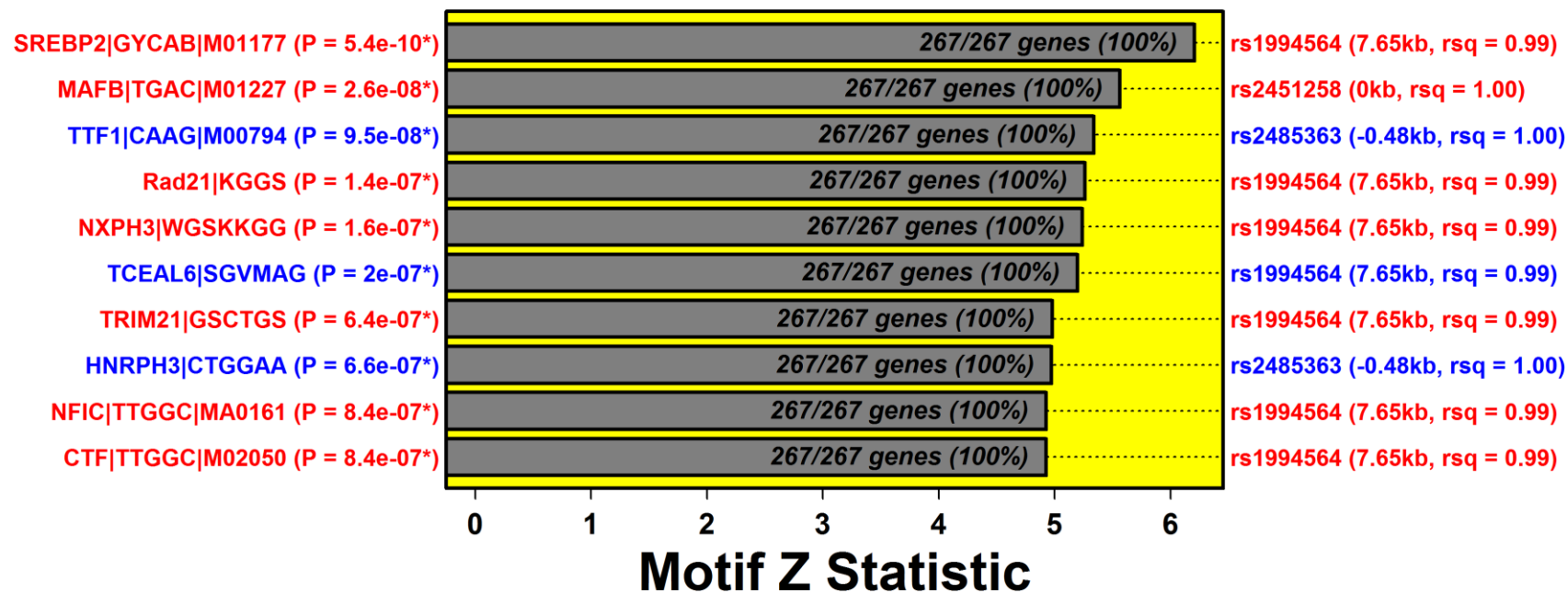

# (E) *KLF4* (rs10979182, Chr 9, 110817020)

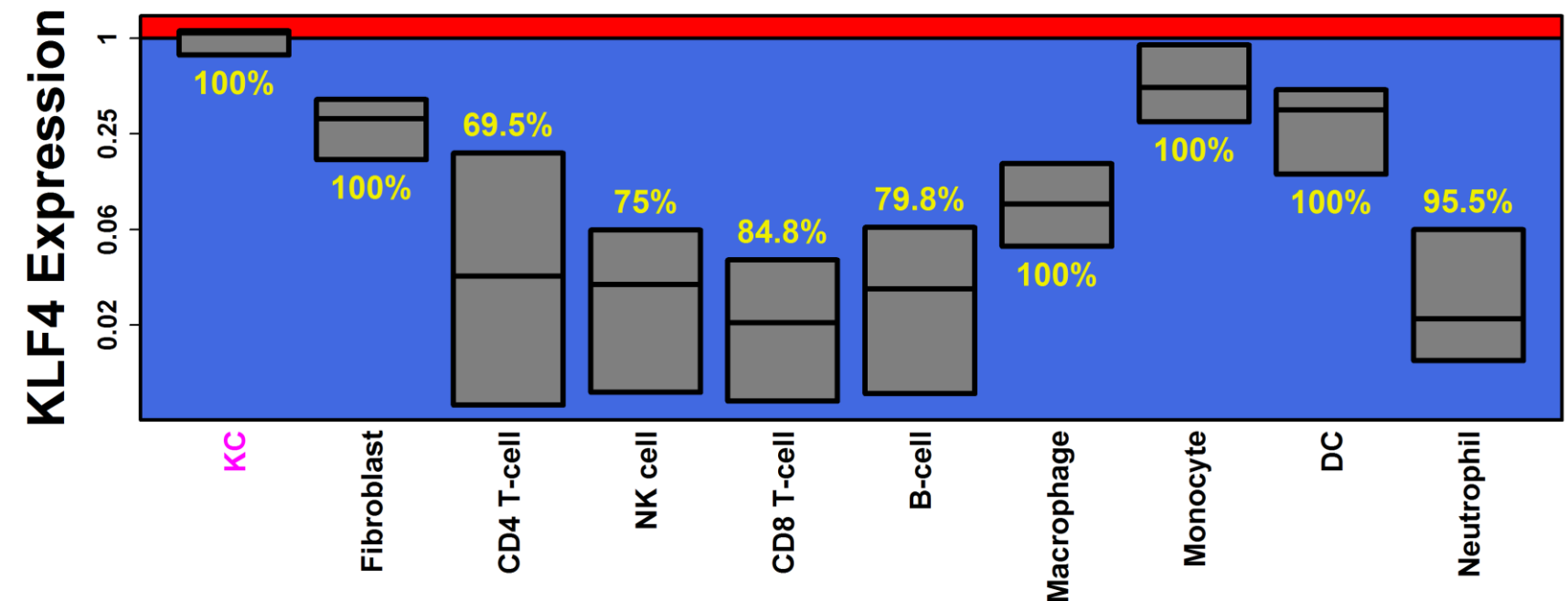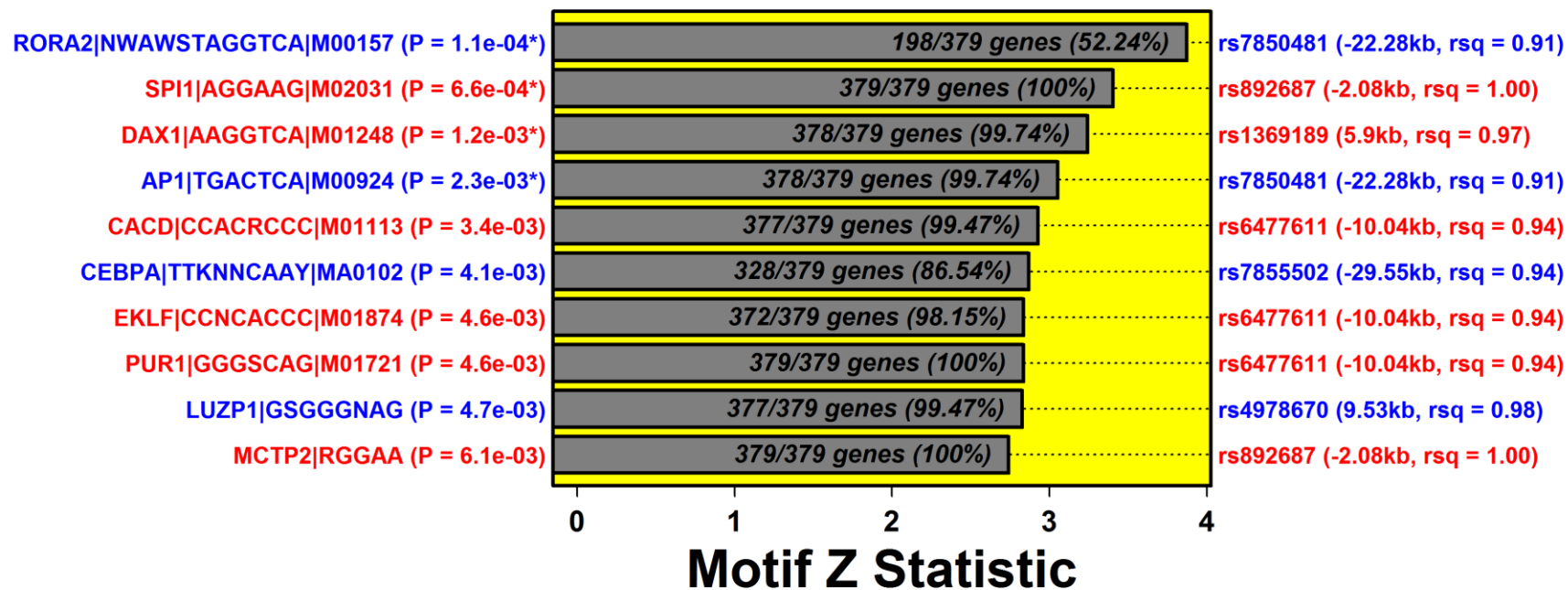

(F) *NFKBIA* (rs8016947, Chr 14, 35832666)

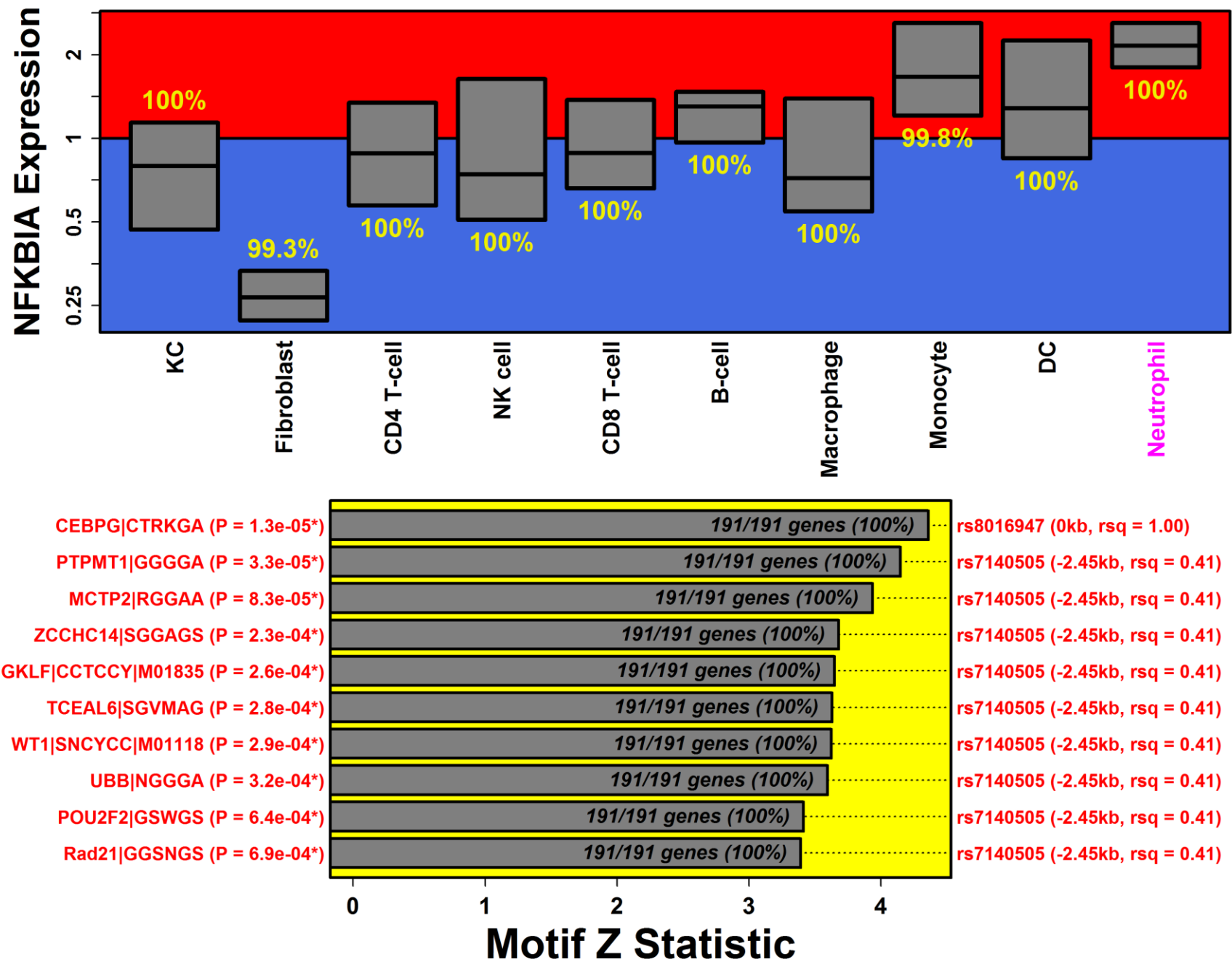

Supplement: Additional file 16 — Identification and prioritization of transcription factor binding sites sensitive to susceptibility-associated variation near psoriasis intergenic risk loci. Procedures outlined in Figures 7 and 8 were repeated with respect to six genes near intergenic risk loci (TNFRSF9, B3GNT2, IL12B, TAGAP, KLF4 and NFKBIA). Top: Expression of each gene was evaluated across 10 cell types (n = 50 microarray samples per cell type). Expression is normalized to that observed in normal human skin and values in yellow denote the gene’s detection frequency. The candidate cell type for each gene is shown in magenta font. Bottom: Top 10 binding sites most strongly enriched among sequences near co-expressed genes identified with respect to the candidate cell type. The listed binding sites include only those that are sensitive to variation at a SNP locus in linkage disequilibrium with the lead SNP (r2 > 0.90). The chart shows the Z statistic quantifying the degree to which each binding site is enriched among sequences adjacent to co-expressed genes. Associated p-values are listed in the left margin, where an asterisk symbol is used to denote FDR < 0.05. The right margin lists the SNP for which variation is predicted to influence binding. Blue font denotes binding sites for which the risk variant abrogates a match to the binding site, while red font denotes binding sites for which the risk variant engenders a match to the binding site. [file 1755-8794-7-27-S16.pdf]

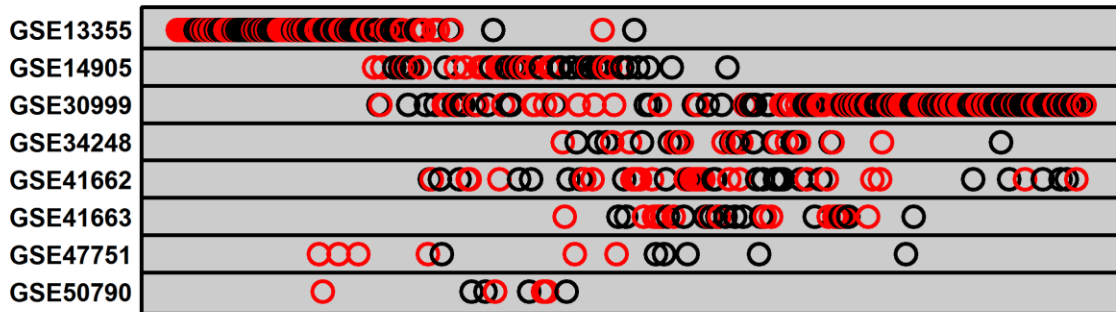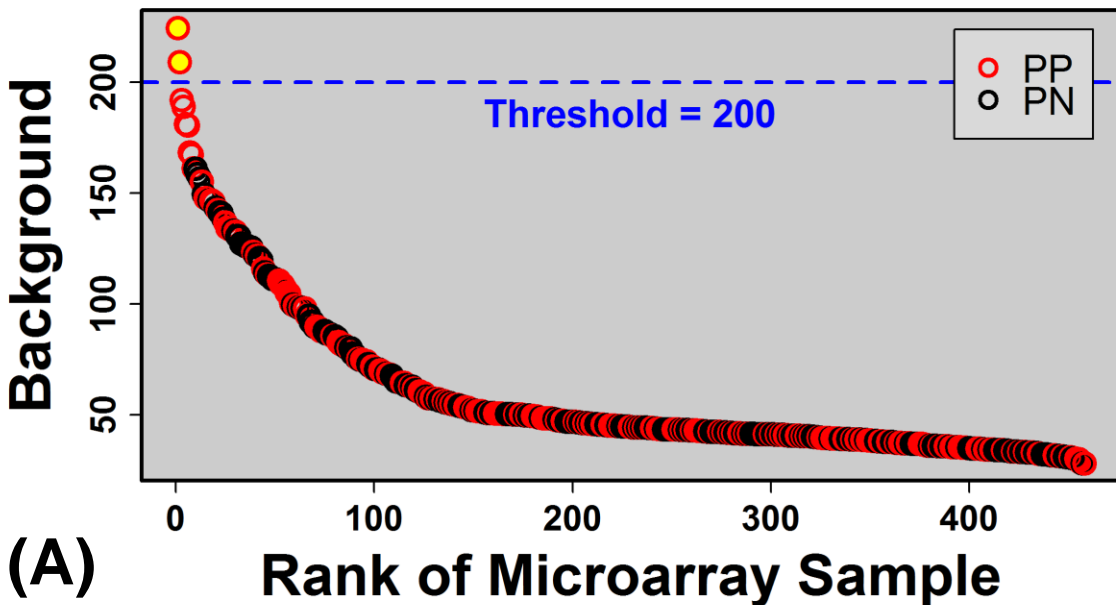

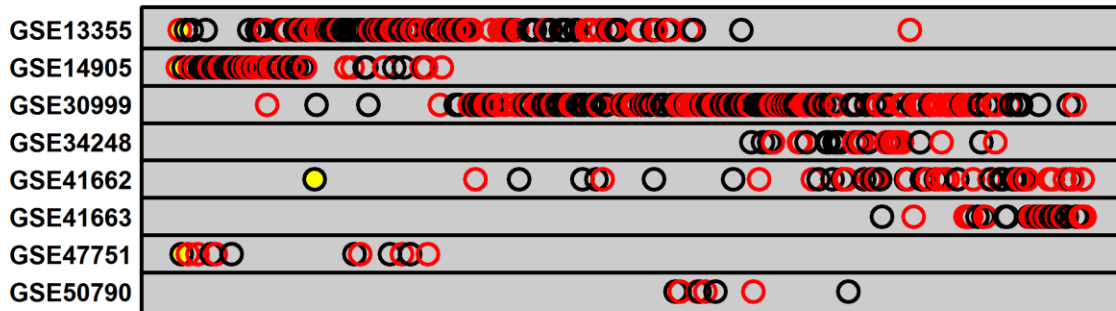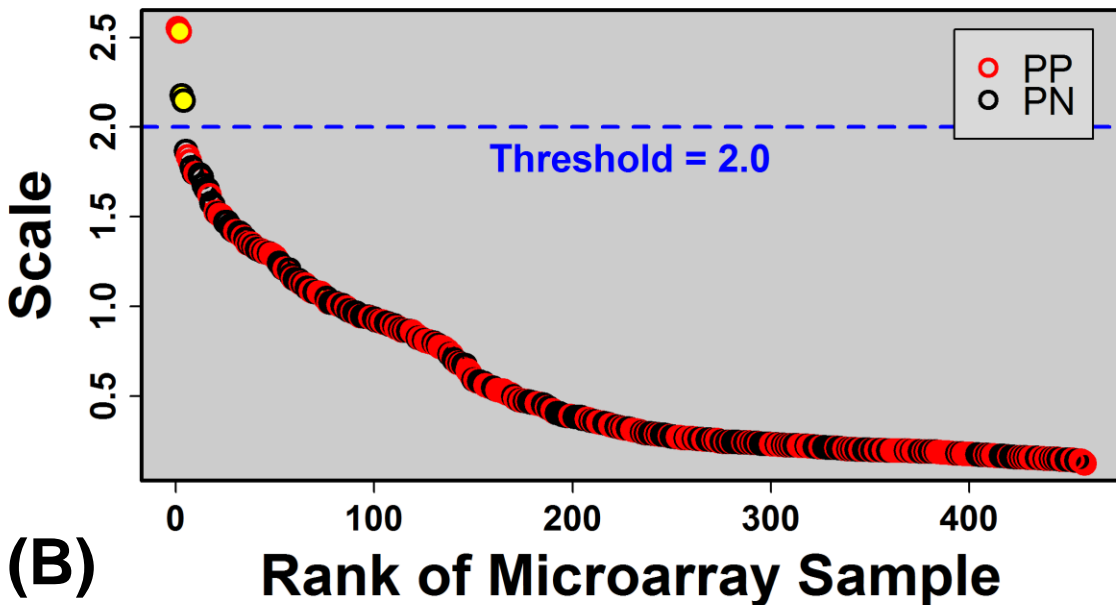

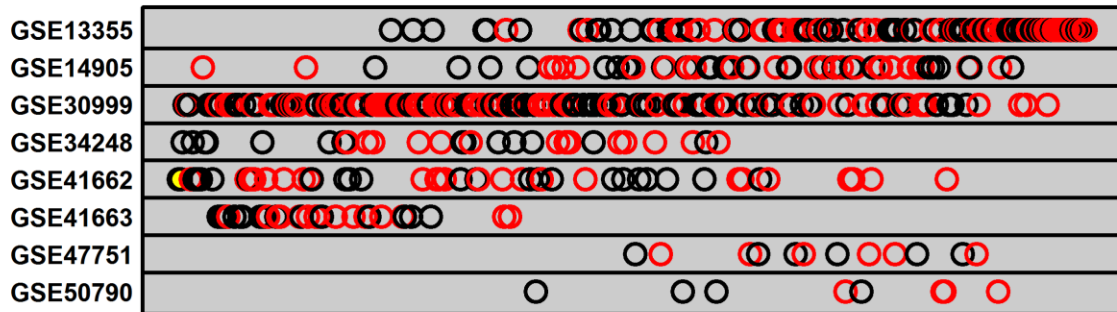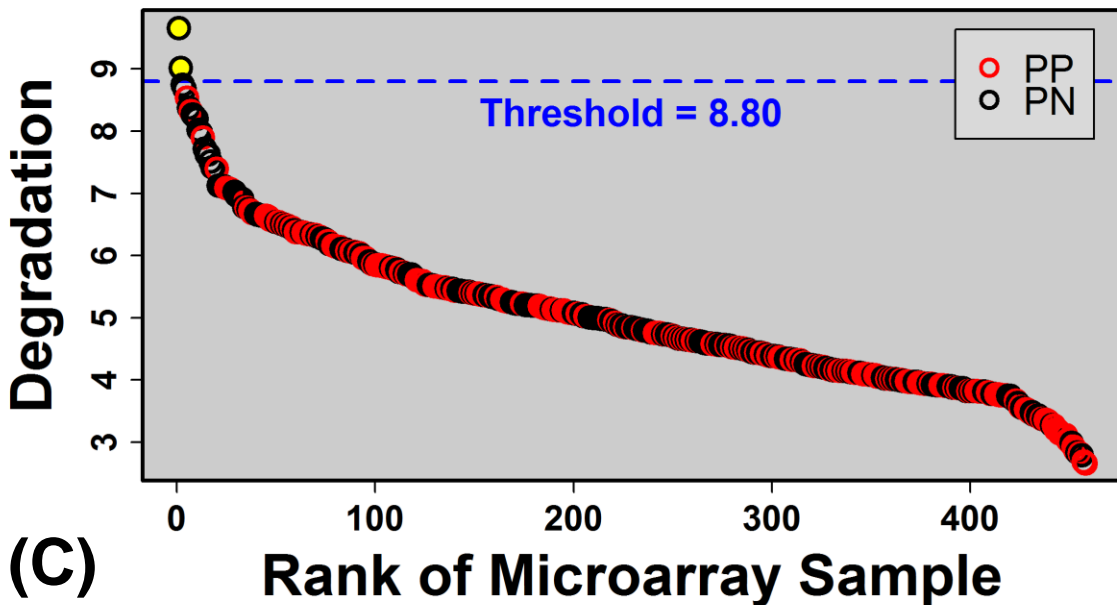

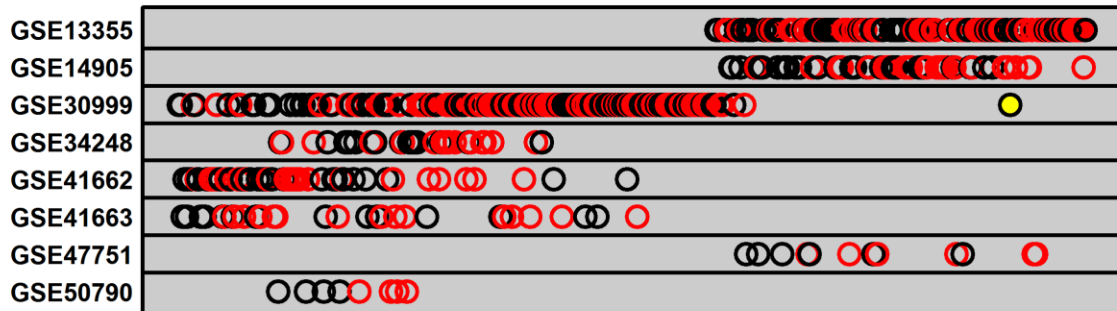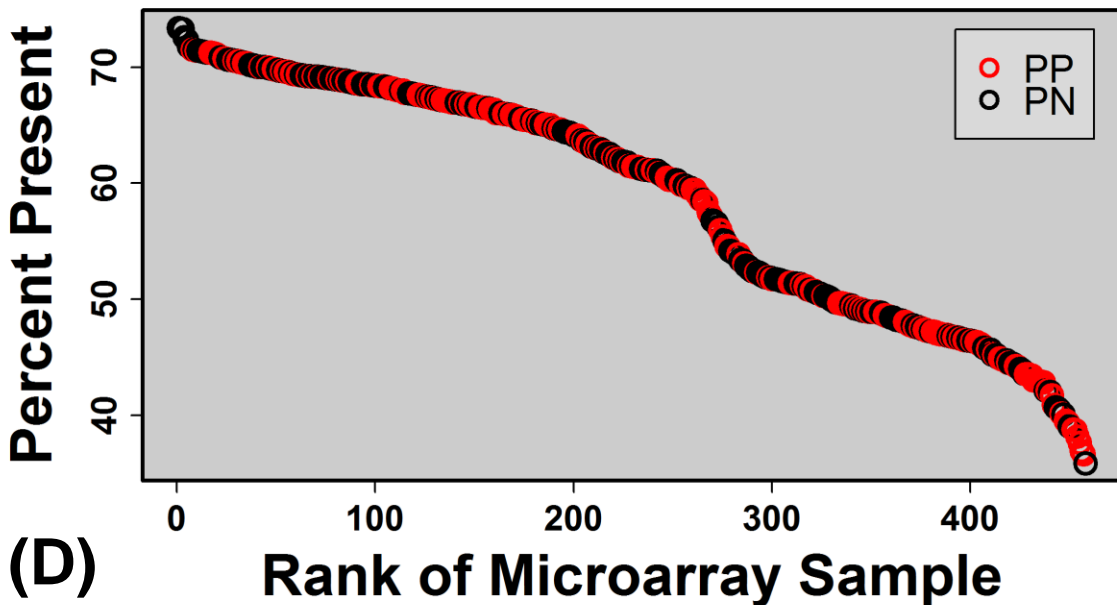

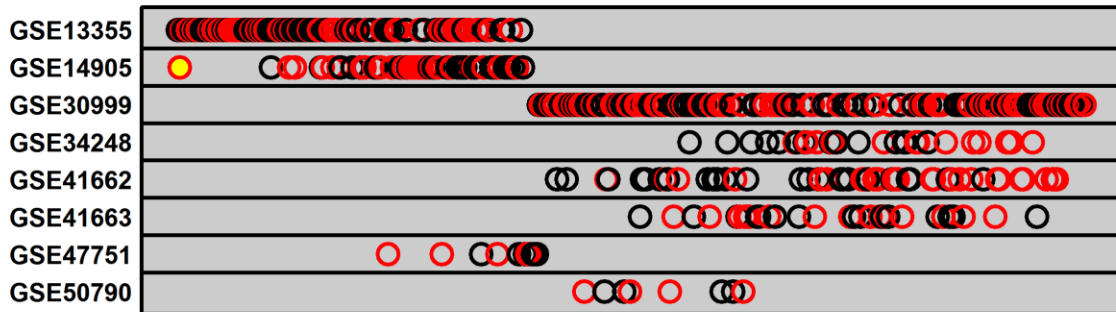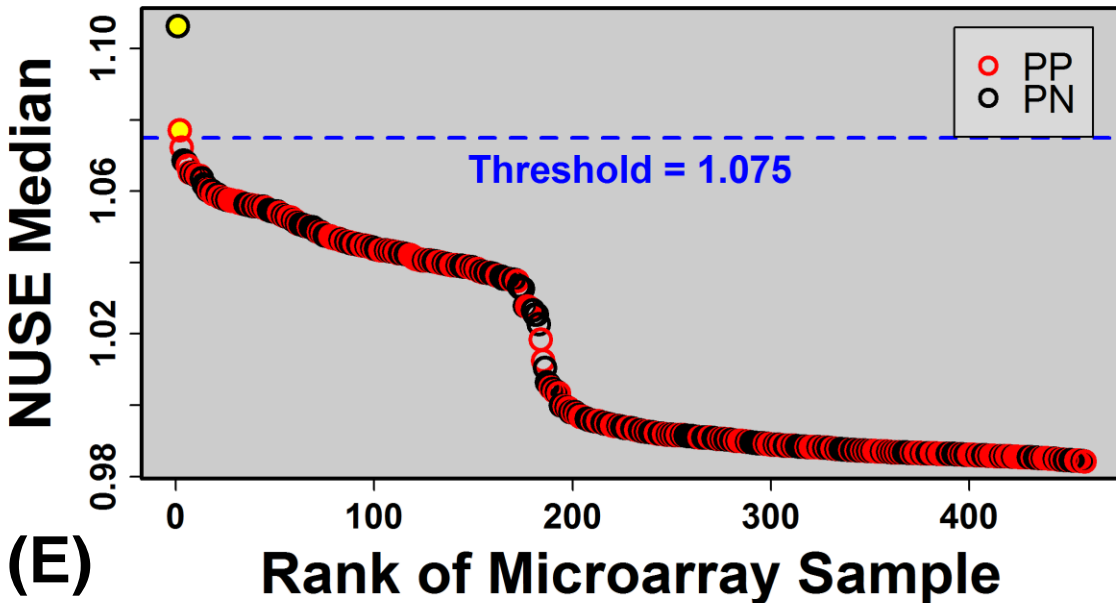

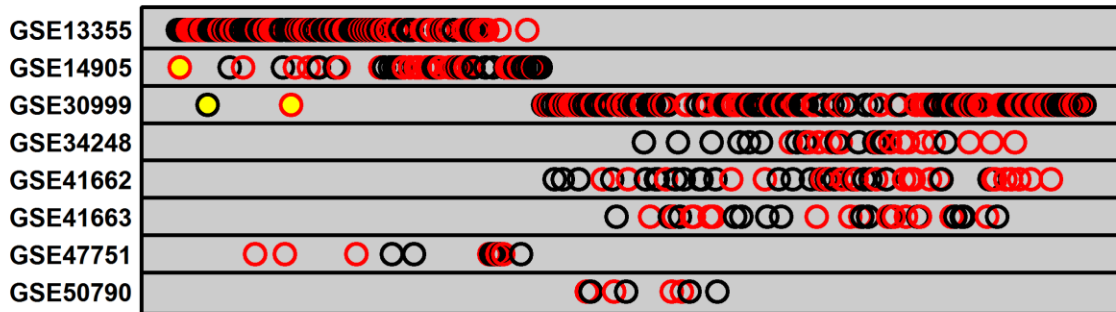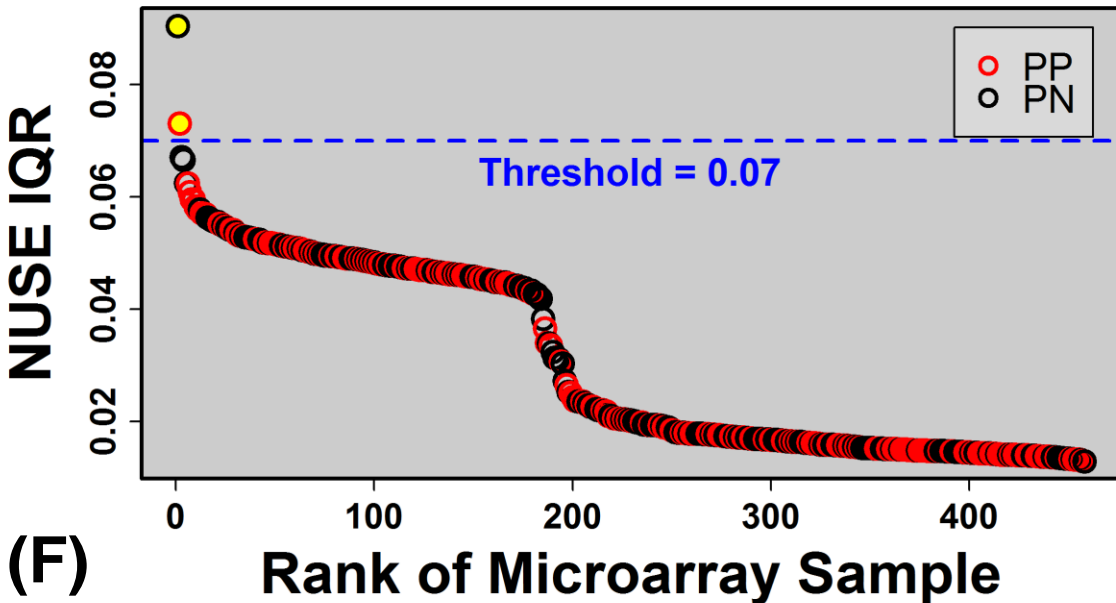

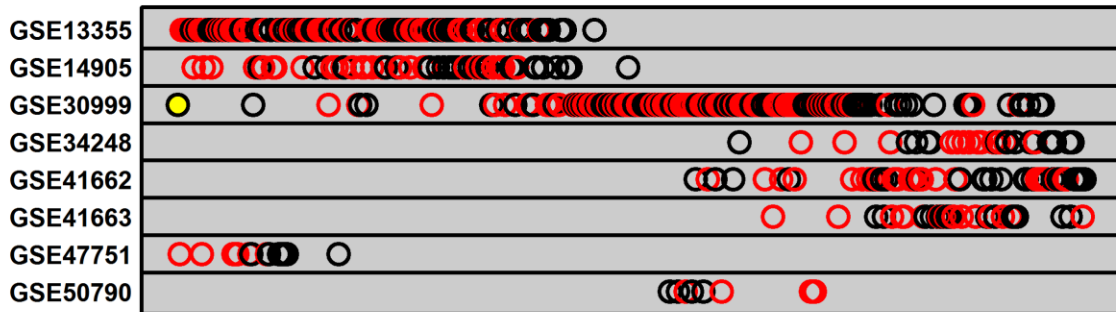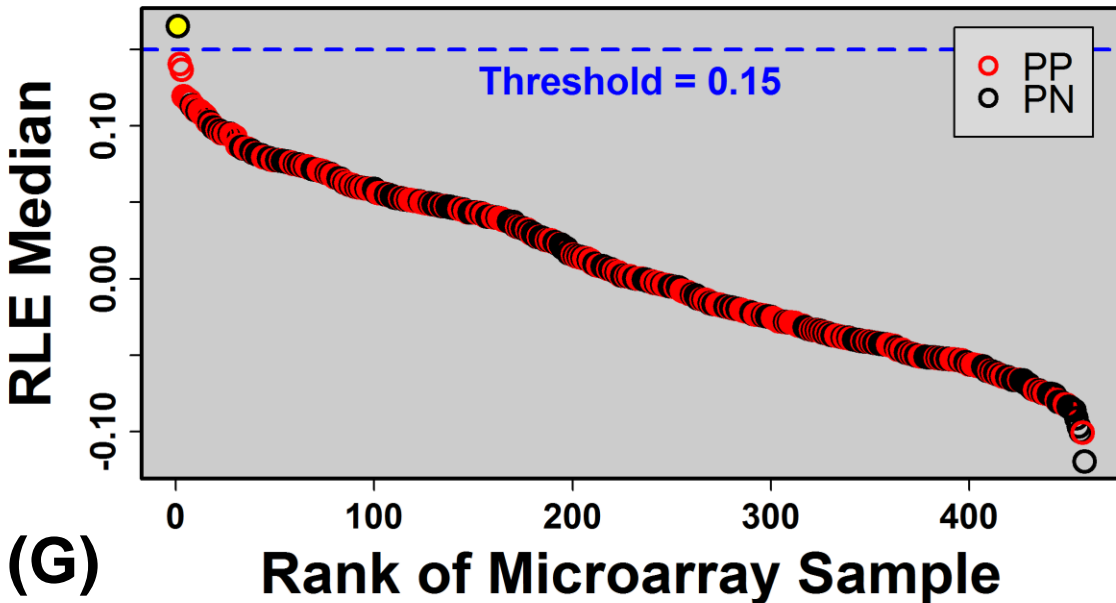

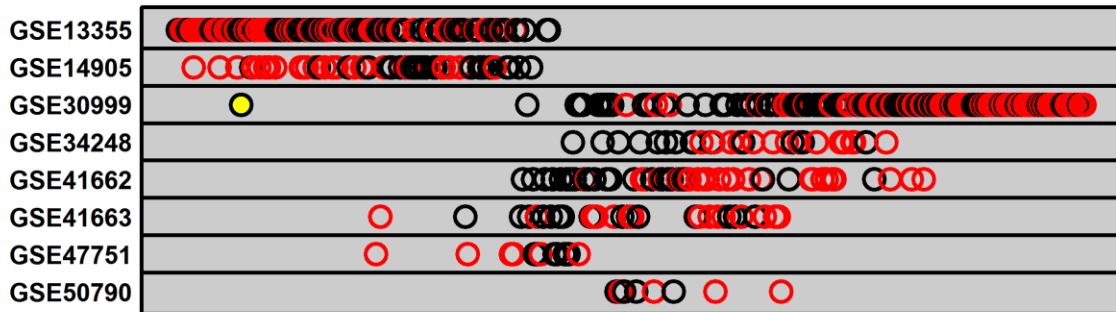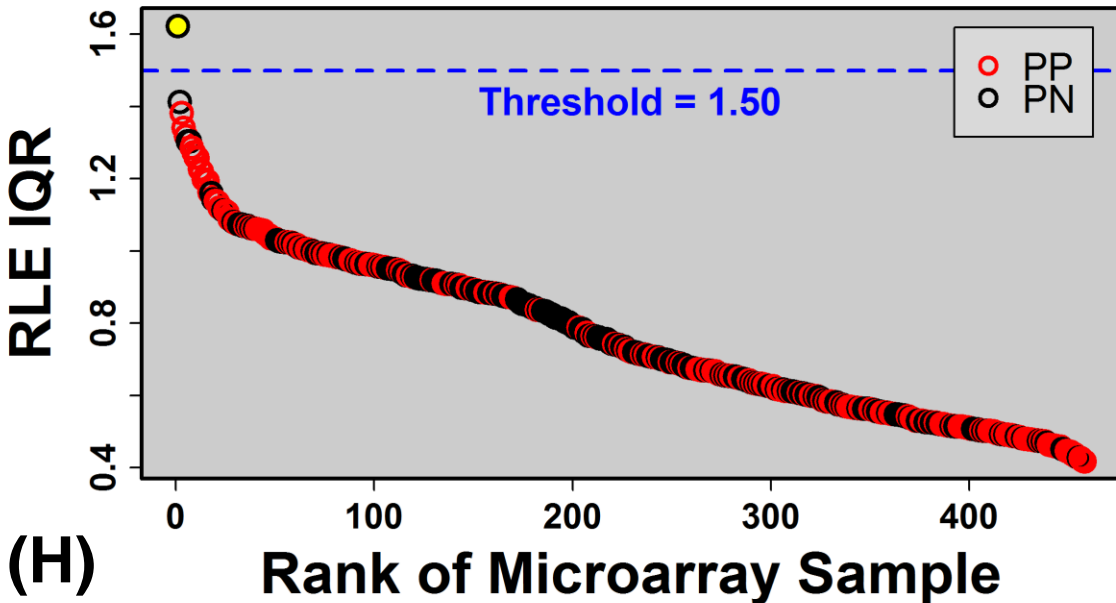

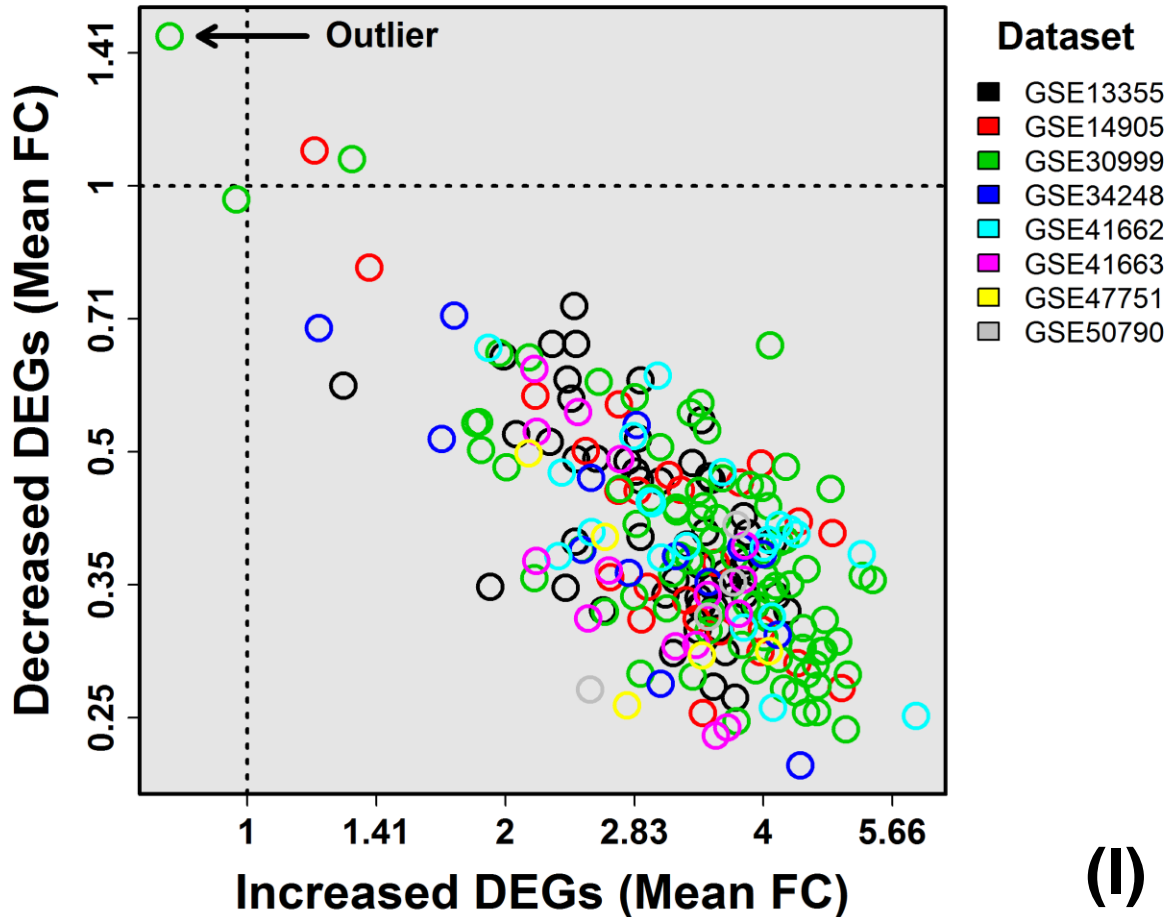

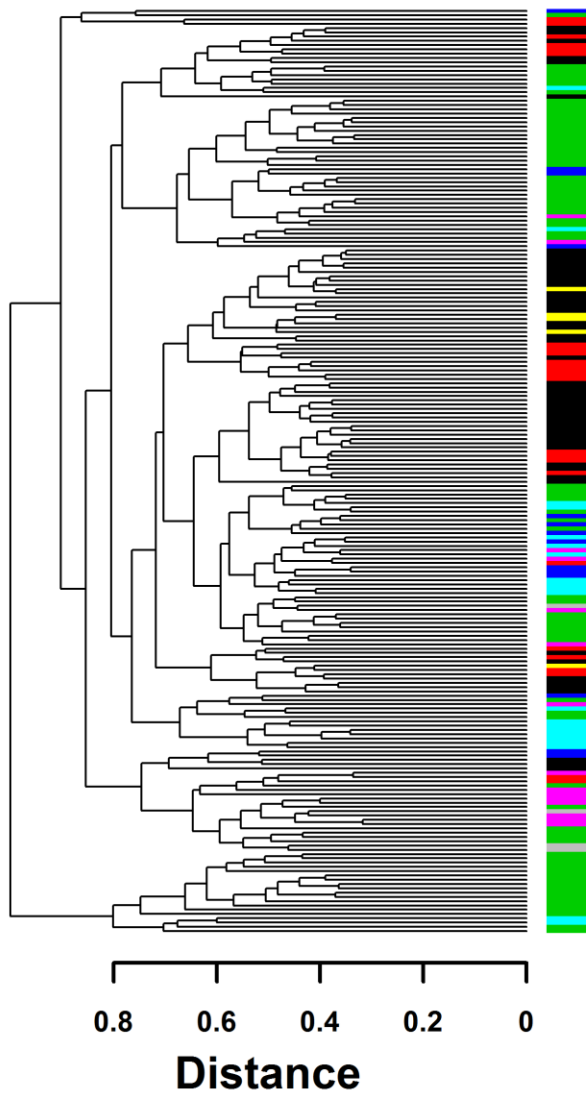

## Dataset

- GSE13355
- GSE14905
- GSE30999
- GSE34248
- GSE41662
- GSE41663
- GSE47751
- GSE50790

(J)

Supplement: Additional file 18 — Quality control (QC) processing of psoriasis microarray data. The dataset was generated by pooling samples across 8 prior studies (GSE13355, GSE14905, GSE30999, GSE34248, GSE41662, GSE41663, GSE47751 and GSE50790), which yielded an initial set of 458 paired PP and PN samples from 229 patients. All samples were generated using the same commercial microarray platform (Affymetrix Human Genome U133 Plus 2.0 array). The 458 samples were evaluated with respect to (A) Average background, (B) Scale factor, (C) RNA degradation score, (D) Percentage of probe sets called present, (E) NUSE median, (F) NUSE IQR, (G) RLE median and (H) RLE IQR. Parts (A) – (H) show QC results for each of these metrics with respect to each dataset individually (top) as well as the combination across all datasets (bottom). Yellow symbols represent microarray samples excluded on the basis of QC criteria. Following these steps, there remained 434 samples from 217 patients. (I) For the remaining patients, we calculated the average fold-change among 434 genes with strongly elevated expression (median FC > 2.0 and FDR < 0.05; n = 217), and the average fold-change among 194 genes with strongly repressed expression (median FC < 0.50 and FDR < 0.05; n = 217). This revealed one outlier patient with trends opposite to those expected. The patient was thus removed from the dataset, yielding 432 samples from 216 patients. (J) The 216 remaining patients were clustered based upon PP versus PN differences observed for 16358 skin-expressed genes (Euclidean distance metric with complete linkage). [file 1755-8794-7-27-S18.pdf]
