# Supplementary material for: Cellular dissection of psoriasis for transcriptome analyses and the post-GWAS era
Source: BMC Med Genomics. 2014 May 22;7:27. doi: 10.1186/1755-8794-7-27 (PMC4060870; doi:10.1186/1755-8794-7-27)
Supplement: Additional file 5 — Number of microarray samples analyzed for each cell type. The table lists the number of microarray samples initially considered for each cell type, as well as the number of microarray samples remaining following quality control checks (post-QC). All samples were generated using same oligonucleotide microarray platform (Affymetrix Human Genome U133 Plus 2.0 Array). Analyses are based only upon the post-QC samples that survived the quality control filtering (final column on right). GEO series accession ids associated with post-QC samples are listed below the table, with the number of samples from each GEO series listed in parentheses. [file 1755-8794-7-27-S5.pdf]

Additional File 5. Number of microarray samples analyzed for each cell type. The table lists the number of microarray samples initially considered for each cell type, as well as the number of microarray samples remaining following quality control checks (post-QC). All samples were generated using same oligonucleotide microarray platform (Affymetrix Human Genome U133 Plus 2.0 Array). Analyses are based only upon the post-QC samples that survived the quality control filtering (final column on right). GEO series accession ids associated with post-QC samples are listed below the table, with the number of samples from each GEO series listed in parentheses (see below).

Cell Type	No. Samples (initial)	No. Samples (post-QC)
Keratinocyte (KC)	137	118[a]
Fibroblast	174	148[b]
CD4+ T-cell	564	495[c]
NK cell	160	140[d]
CD8+ T-cell	151	132[e]
B-cell	385	341[f]
Macrophage	377	332[g]
Monocyte	514	454[h]
Dendritic cells (DC)	421	368[i]
Neutrophil	487	426[j]

[a]GSE30355(26), GSE7216(25), GSE36287(18), GSE33495(10), GSE21364(9), GSE37637(6), GSE32685(6), GSE21567(5), GSE36222(4), GSE34528(4), GSE27186(2), GSE18590(2), GSE33536(1)

[b]GSE17032(26), GSE27041(19), GSE20538(17), GSE11854(13), GSE16715(12), GSE9762(10), GSE16447(9), GSE34308(8), GSE23741(8), GSE16524(8), GSE41751(6), GSE13606(5), GSE9709(2), GSE9451(2), GSE27280(1), GSE18226(1), GSE17549(1)

[c]GSE13732(97), GSE11292(80), GSE23293(41), GSE10586(26), GSE12079(23), GSE14924(19), GSE27928(17), GSE14278(17), GSE24223(15), GSE31773(12), GSE13738(12), GSE25087(10), GSE22025(10), GSE15735(9), GSE12963(9), GSE42853(8), GSE28726(8), GSE26928(8), GSE16461(8), GSE14879(7), GSE9927(6), GSE14596(6), GSE6338(5), GSE28491(5), GSE19069(5), GSE15659(5), GSE42569(4), GSE28490(4), GSE22045(4), GSE12875(4), GSE10437(4), GSE7307(2), GSE42253(2), GSE17718(2), GSE16130(1)

[d]GSE27838(29), GSE41914(24), GSE35330(20), GSE12198(16), GSE30950(14), GSE21774(9), GSE20499(6), GSE28490(5), GSE8059(4), GSE22919(4), GSE15743(4), GSE28491(3), GSE42253(2)

[e]GSE24223(22), GSE31773(20), GSE14924(20), GSE26495(14), GSE33424(9), GSE16461(7), GSE26890(6), GSE39508(5), GSE28491(5), GSE28490(5), GSE19069(5), GSE6338(4), GSE14879(4), GSE6920(2), GSE10178(2), GSE7307(1), GSE16130(1)

[f]GSE39411(48), GSE15490(40), GSE36907(26), GSE30153(26), GSE31311(19), GSE45113(17), GSE17186(14), GSE24223(13), GSE12366(12), GSE17269(11), GSE12195(9), GSE38697(8), GSE15271(8), GSE13987(8), GSE24736(7), GSE46143(6), GSE27670(6), GSE25638(6), GSE24044(6), GSE13917(6), GSE10821(6), GSE46062(5), GSE28491(5), GSE28490(5), GSE26725(5), GSE13300(5), GSE29301(4), GSE9119(3), GSE12453(3), GSE10831(3), GSE19599(1)

[g]GSE2125(44), GSE13896(42), GSE16385(36), GSE11886(33), GSE13670(30), GSE7568(24), GSE8823(23), GSE22342(16), GSE11199(12), GSE24897(10), GSE30536(9), GSE4883(8), GSE16386(8), GSE14390(7), GSE16755(6), GSE8608(5), GSE11430(5), GSE20484(4), GSE10856(4), GSE9801(3), GSE41889(3)

[h]GSE9988(60), GSE6751(42), GSE34156(41), GSE38351(37), GSE24223(26), GSE7158(25), GSE6054(23), GSE12838(17), GSE34025(16), GSE35683(15), GSE11755(14), GSE8658(12), GSE12108(11), GSE28490(10), GSE24869(10), GSE11864(9), GSE7807(8), GSE34407(8), GSE17549(7), GSE16836(7), GSE32939(6), GSE18565(6), GSE13899(6), GSE7509(5), GSE28491(5), GSE11430(5), GSE21351(4), GSE9801(3), GSE9390(3), GSE41889(3), GSE22373(3), GSE13762(3), GSE7307(2), GSE45535(2)

[i]GSE8658(45), GSE18791(40), GSE14000(22), GSE42924(21), GSE23073(20), GSE7509(19), GSE42088(15), GSE10316(13), GSE5547(12), GSE4984(12), GSE13762(12), GSE11309(12), GSE5679(11), GSE23371(11), GSE7247(10), GSE12773(10), GSE23618(9), GSE24132(8), GSE22589(8), GSE6090(6), GSE39999(6), GSE22282(6), GSE10147(6), GSE6863(5), GSE40268(5), GSE28490(5), GSE6965(4), GSE10463(4), GSE34515(3), GSE11327(3), GSE35340(2), GSE13296(2), GSE42058(1)

[j]GSE16837(99), GSE8507(85), GSE37416(42), GSE11083(39), GSE33939(26), GSE8668(24), GSE22103(21), GSE16020(20), GSE39889(16), GSE35683(15), GSE12838(9), GSE18810(6), GSE39840(5), GSE14465(5), GSE28491(4), GSE26975(4), GSE12662(4), GSE28490(2)
