# Supplementary material for: Cellular dissection of psoriasis for transcriptome analyses and the post-GWAS era
Source: BMC Med Genomics. 2014 May 22;7:27. doi: 10.1186/1755-8794-7-27 (PMC4060870; doi:10.1186/1755-8794-7-27)
Supplement: Additional file 17 — Demographic data and washout protocols for psoriasis microarray cohorts. The table lists the number of patient samples included from each of the eight microarray studies (n). The table also lists known demographic data on each patient cohort and the disease severity of patients admitted into each study. The final two columns indicate reported washout protocols. This represents the period of time for which participants discontinued systemic or topical medications prior to biopsy collections. In some cases, information was not provided in original research reports and thus it was not possible to provide a complete description for all studies. [file 1755-8794-7-27-S17.pdf]

Additional File 17. Demographic data and washout protocols for psoriasis microarray cohorts. The table lists the number of patient samples included from each of the eight microarray studies (*n*). The table also lists known demographic data on each patient cohort and the disease severity of patients admitted into each study. The final two columns indicate reported washout protocols. This represents the period of time for which participants discontinued systemic or topical medications prior to biopsy collections. In some cases, information was not provided in original research reports (see below) and thus it was not possible to provide a complete description for all studies.

GEO Series	*n*	% Male	Age	Severity	Washout (systemic)	Washout (topical)
GSE13355[a]	54	49.1%	mean: 48.9, range: 21 - 69	mild - severe	2 weeks	1 week
GSE14905[b]	25	?	?	?	?	?
GSE30999[c]	78	77.5%	mean: 44.6	moderate - severe	4 weeks	2 weeks
GSE34248[d]	14	71.4%	range: 23 - 71	mild - moderate	no medications[†]	no medications[†]
GSE41662[d]	21	?	range: 19 - 55	moderate - severe	?	?
GSE41663[d]	15	?	range: 18 - 70	moderate - severe	4 weeks	4 weeks
GSE47751[e]	5	66.7%	mean: 45.3, range: 26 - 68	mild - severe	4 weeks	2 weeks
GSE50790[f]	4	50%	mean: 50, range: 44 - 64	mild - severe	2 weeks	1 week

[a]Nair et al. 2009, Nat Genet 41:199-204
[b]Yao et al. 2008, PLoS ONE 3:e2737
[c]Suárez-Fariñas et al. 2012, J Invest Dermatol 132:2552-2564
[d]Bigler et al. 2013, PLoS ONE 8:e52242
[e]Johnston et al. 2014, Br J Dermatol, In Press
[f]Swindell et al. 2012, PLoS ONE 7:e34594
[†]Patients enrolled in the GSE34248 cohort did not report using any medications upon entry into the study.
